# Geometric deep learning-enabled metal-binding site identification and grafting

**DOI:** 10.1016/j.fmre.2024.11.012

**Published:** 2024-11-29

**Authors:** Jun-Lin Yu, Yao-Geng Wang, Jian Peng, Jing-Wei Wu, Cong Zhou, Guo-Bo Li

**Affiliations:** Key Laboratory of Drug-Targeting and Drug Delivery System of the Education Ministry and Sichuan Province, Department of Medicinal Chemistry, West China School of Pharmacy, Sichuan University, Chengdu 610041, China

**Keywords:** Metal-binding site, Metalloenzyme, Geometric deep learning, Artificial metalloenzyme, Metalloprotein

## Abstract

Metal-binding sites can participate in enzymes’ catalytic reactions, as well as in protein folding, stability, and protein-protein interactions. In-depth research on metal-binding sites can aid in identifying new protein functions and designing new metalloproteins. We present MeSiteIG, a geometric deep learning-driven bifunctional tool with capabilities for both Metal-binding Site Identification and Grafting, established based on the geometric conservation of metal-binding sites. It comprises three main modules: residue triplet searcher (ResTriS), metal-binding site identifier (MeSI), and metal-binding site alignment (MeSA). As the core module, MeSI adopts E3-equivarient graph neural networks to predict potential metal-binding site residues, without the coordinating residue types and side-chain information. MeSI achieved superior performance on the independent test set, with an impressive speed of approximately 300 samples per second. Using MeSiteIG, we identified previously neglected protein metal-binding sites and potential protein bimetallic sites. Furthermore, it could graft metal sites onto antibody surfaces and protein pockets, creating new potentially valuable metalloproteins.

## Graphical abstract

**Figure:**
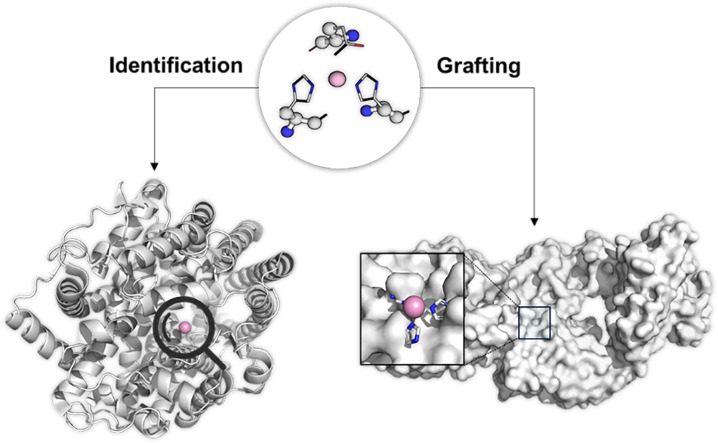

## 1 Introduction

Metalloenzymes constitute a ubiquitous ensemble of enzymes that rely on metal-binding sites to participate in catalytic reactions directly or indirectly [1]. Metal-binding sites with catalytic activity are typically formed by metal ions (most commonly transition metal ions) or cofactors (e.g., heme) coordinated with protein residues. Metal-binding sites may also exist within proteins without exhibiting catalytic activity, but they play crucial roles in protein folding and/or structural stability, partly similar to disulfide bonds. Additionally, metal-binding sites are also present on the surface of proteins, influencing protein-protein interactions, signal transduction, or other functions [2]. Studies on metal-binding sites are crucial for many socially important fields. For instance, in synthetic biology, the introduction of metal-binding sites to create new artificial metalloenzymes enables the achievement of novel biocatalytic transformations not found in nature [3].

Based on the PDB resources, Andreini et al. [[4], [5]] pioneered the data mining of metal-binding sites, including coordination geometries, equistructural and equivalent metal-binding sites, resulting in a pool of > 380,000 metal-binding sites. We previously concentrated on metalloenzymes, analyzing their metal-binding sites, structural features and ligands, thus forming a reservoir of metalloenzyme-specific knowledge [6]. To enhance the understanding of metal-binding sites, we developed a tool called MeCOM for comparing metalloenzymes by aligning metal-binding sites and catalytically associated residues [7], which is particularly useful for identifying and inferring the function of new metalloenzymes. Similarly, Lin et al. [8] proposed a series of tools, named MIB and MIB2 [9], for searching metal-binding sites by comparing regional structures between queried structures and collected templates from the PDB database, and these tools have been shown to provide reliable prediction results for most types of metal ions. Based on structure or sequence information, there are growing efforts devoted to exploring and identifying metal-binding sites with the aid of machine learning methods. Wang group innovatively developed MetalNet [10], a co-evolution-based machine learning method for predicting potential metal-binding sites in proteomes, which enables the interrogation of the hidden metalloproteome and facilitates the study of metal biology. Rothlisberger and co-authors [11] developed Metal3D to predict metal ion location using a 3D convolutional neural network and geometric criteria, which has been successfully applied for annotating AlphaFold2 structures and for metalloprotein engineering. The latest version of AlphaFold3 [12] and RFAA [13] models can predict the 3D structure of proteins complexed with metal ions directly from sequence information. Additionally, metal-binding site information has been used for the *de novo* design of artificial metalloenzymes [3].

We here present MeSiteIG, a bifunctional tool for metal-binding site identification and grafting, established based on the geometric conservation of metal-binding sites. To achieve both metal site identification and grafting, we focused on the classical three-residue metal sites, by specifically considering residue triplets consisting solely of the four main atoms ($C_{\alpha }$, $C_{\beta }$, amide N, and carbonyl C) of each involved residue. Generally, MeSiteIG comprises three key components: residue triplet searcher (ResTris), metal-binding site identifier (MeSI), and metal-binding site alignment (MeSA). ResTris functions to search for residue triplets in protein pockets or surfaces. By learning the geometric relationships between the main atoms of metal-binding site residues *via* an E3-equivarient graph neural network (GNN), MeSI enables the prediction of potential metal-binding site residues, without knowledge of the coordinating residue types and side-chain information. MeSI exhibited superior performance in distinguishing between metal-binding site and non-metal-binding site residues, achieving an impressive processing speed of approximately 300 samples per second. MeSA was established based on metal-binding site geometric alignment to determine the type and position of metal ions and coordination residues. Combining the complementary advantages of MeSI and MeSA, MeSiteIG can not only rapidly and accurately identify metal-binding sites for proteins of interest, but also accurately graft metal-binding sites without altering the protein's backbone, generating new potential metalloproteins. We applied MeSiteIG to explore the PDB database, and found that a batch of mono-nuclear metalloenzymes contain di-nuclear metal-binding sites, which may transform into di-nuclear metalloenzymes when the corresponding metal ions in the environment reach a certain concentration. By using MeSiteIG, we achieved metal-binding site grafting in the surface of given antibodies and the pockets of non-metalloenzymes. These case studies clearly illustrate MeSiteIG as a versatile tool for expanding research on metal-binding sites in the fields of chemistry, biology, and medicine.

## 2 Materials and methods

### 2.1 Dataset construction

**Fig. 1:**
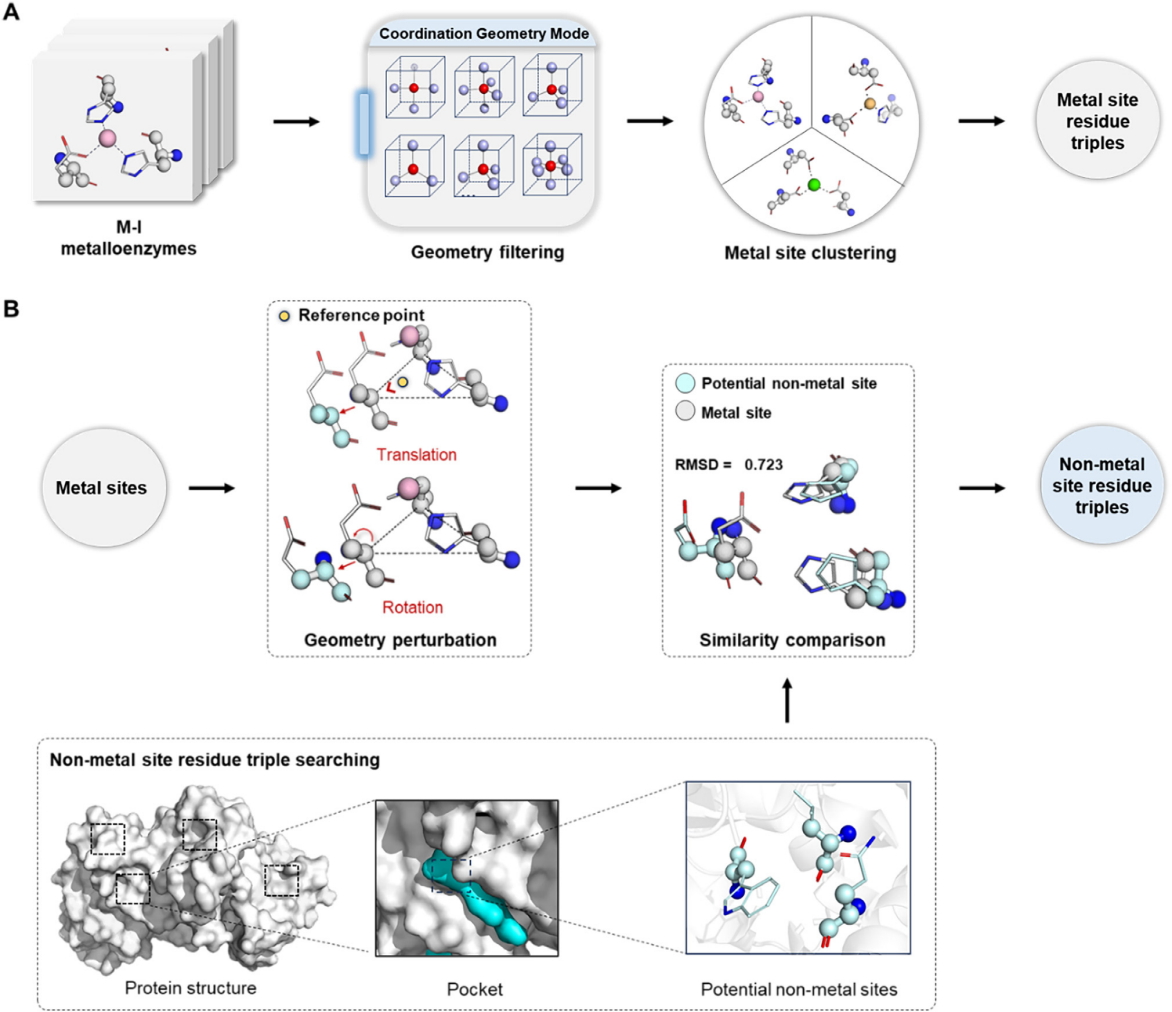
**Dataset construction procedures for (A) metal-binding site residue triplets and (B) non-metal-binding site residue triplets**.

We began with construction of a database containing metal-binding site and non-metal-binding site residue triplets for MeSI model training. We concentrated on three-residue metal sites as they accounted for the majority of the retrieved metal sites. Here, only main atoms including $C_{\alpha }$, $C_{\beta }$, amide N, and carbonyl C are considered in the residue triplets. We prepared metal-binding site residue triplets by the following procedures (Fig. 1A): (1) selecting category M-I metalloenzymes from our MeDBA database [6] to extract metal ion-(tightly)bound metal-binding sites; (2) only retaining metal-binding sites with three coordinating residues, while excluding the cases where coordinating atoms are located on backbone; (3) only retaining metal-binding sites with normal coordination geometries conforming to one of 36 types of ideal coordination geometry modes [[14], [15]]; (4) clustering metal-binding sites according to metal ion-coordinating residues and their geometry: classifying the above-filtered metal-binding sites according to the pattern of metal ion-coordinating residues (e.g., ‘Zn:His-His-His’), followed by 3D superimposition of the coordinates of metal ion and coordinating residues within the same class, through MeCOM [7] to calculate the similarity of metal-binding site geometry denoted as AScore in MeCOM; (5) selecting non-redundant metal-binding sites from each of the geometry similar clusters using an AScore cutoff of 2.9 to eliminate duplicate sites or extremely similar metal sites while preserving the diversity of the remaining metal-binding sites; (6) generating metal-binding site residue triplets by ignoring metal ions and non-main atoms. At this point, a total of 2,577 metal-binding site residue triplets were obtained.

The non-metal-binding site residue triplets (not conforming to geometric features of metal-binding site coordinating residues) were prepared from metal-binding site residue triplets as outlined in Fig. 1B. Here we introduced a perturbation algorithm to sample random transformation (rotation and translation) schemes to generate non-metal-binding site residue triplets. Translation involves randomly selecting a reference point (e.g., the geometric center of the backbone, the geometric center of all atoms in the residue, or the metal ion), and then translating each residue outward along the direction from the reference point to its $C_{\alpha }$ atom. Rotation refers to randomly rotating residues along the $C_{\alpha }$-metal ion (M) axis or increasing the $∠C_{\beta }C_{\alpha }M$ angle of each residue. The perturbation operations ensure that the generated non-metal-binding site residue triplets exhibit structural differences from the original metal-binding sites. To further improve the quality of non-metal-binding site samples, we compared the generated potential non-metal-binding sites with metal-binding sites by computing root-mean-square deviation (RMSD) of their main atoms using MeSA. The RMSD cutoff was determined by human inspection after testing different upper and lower limits to identify an appropriate range. This ensured that the negative samples were not significantly different from the original positive ones, allowing the model to learn the subtle discrepancies between positive and negative samples. The final threshold values of RMSD were set based on the number of perturbed residues (see threshold values in Supplementary Table S1). This strategy ensured that the final non-metal-binding site samples differed from metal-binding site samples while still retaining a certain degree of structural similarity to metal-binding sites (with the aim to increase discrimination capability of the MeSI model). Meanwhile, we randomly selected non-metal-binding sites from protein pockets to enrich the diversity of the dataset; the samples with RMSD values ranging from 0.4 to 0.8 were remained (Fig. 1B). Ultimately, we obtained 25,767 non-metal-binding site residue triplets.

The final dataset comprises a total of 28,344 samples, encompassing both metal and non-metal-binding sites. We randomly extracted 1,837 samples as an independent test set, while the remaining 26,507 samples were divided into training and validation sets at a ratio of 4:1. Furthermore, 20 proteins were selected from another independent test set, from which all possible residue triplets were extracted with ResTris. These triplets were then labelled by comparing with known metal-binding sites with a strict cutoff (RMSD < 0.4 Å) using MeSA, resulting in a second test set for further evaluation.

### 2.2 MeSI network architecture

*(1) Model framework*

In MeSI, we regard a metal-binding site or non-metal-binding site sample consisting only of main atoms of the residues ($C_{\alpha }$, $C_{\beta }$, amide N, and carbonyl C) as a graph G for prediction of potential metal-binding sites, without knowledge of the coordinating residue types and side-chain information. Within G, each node represents an individual residue in a residue triplet, and the edges represent the spatial relationships between residues. The node scale attributes include the angles (e.g., $∠C_{\beta }C_{\alpha }N$) and distances between main atoms of each residue, and the vector attributes consist of the directional vectors from $C_{\alpha }$ to the other main atoms ($\left[x_{C_{\alpha }\to C_{\beta }},\;x_{C_{\alpha }\to C},x_{C_{\alpha }\to N}\right]$) within each residue. The edge scalar attributes are derived from three main components, calculated using local frames: the relative orientation, which considers how the local orientations of residues align by comparing the orientations of their key structural axes; the relative position, determined by the differences in their spatial coordinates; and the distance between the $C_{\alpha }$ atoms of residues. The vector attributes consist of directional vectors between $C_{\alpha }$ of different residues.

Considering the significance of E3 equivariance, we used EGNN or GVP as the Embedding module. The aforementioned vector attributes of nodes or edges are not considered unless GVP is adopted as the feature updating module. Through EGNN or GVP, the acquired node features can integrate essential geometric and spatial information, thereby enhancing subsequent model learning [16].

We next employed a graph isomorphism network variant considering edge attributes in message passing phase (GINE) [17]. After updating by EGNN or GVP, G is fed into GINE, generating $v^{l}$ for different layers. After comparing three readout methods, mean, max, and sum, s is formed by summing up the outputs $v^{l}$ from each layer and is then processed through a softmax operation. The output y as a binary classification (metal-binding site or non-metal-binding site) probability is obtained.

Specifically, given an input graph G, $x_{i}^{l}\in R^{N\times d}$ represents *d*-dimensional feature of the i-th node in the l layer, and $e_{i,j}^{l-1}$ represents the *k*-dimensional edge feature connecting node *i* and node *j* in the l−1 layer. A GINE with *L* layers updates the node embeddings as follows:

(1) $$x_{i}^{l}=h_{\Theta }\left(\left(1+\epsilon \right)\cdot x_{i}^{l-1}+\sum_{j\epsilon N\left(i\right)}ReLU\left(x_{j}^{l-1}+e_{i,j}^{l-1}\right)\right)$$

where ϵ controls the relative weight of the node itself in information propagation, $h_{\Theta }\;$ is a multi-layer perceptron (MLP) layer altering the dimension of node embeddings. In each layer, the node embeddings $x^{l}=\left\{x_{i}^{l}\right\}$ are summed across all nodes to obtain the graph embedding $v^{l}$ of each layer as given by the following formula:

(2) $$v^{l}=\sum_{i}x_{i}^{l}$$

All the graph embeddings from different layers are added up to combine s. s is then fed into a MLP layer ϕ and a softmax layer [18] to get a normalized probability y.

(3) $$y=Softmax\left(\phi \left(\sum_{l=1}^{L}v^{l}\right)\right)$$

*(2) Model evaluation*

To evaluate MeSI, six metrics for binary classification task are adopted: accuracy, precision, recall, Matthews correlation coefficient (MCC), area under the receiver operating characteristic curve (ROC-AUC), and area under the precision-recall curve (PR-AUC), which are calculated using the following formulas:

$$Accuracy=\frac{TP+TN}{TP+TN+FP+FN}$$

$$Precision=\frac{TP}{TP+FP}$$

(4) $$Recall=\frac{TP}{TP+FN}$$

$$MCC=\frac{TP\cdot TN-FP\cdot FN}{\sqrt{\left(TP+FP\right)\cdot \left(TP+FN\right)\cdot \left(TN+FP\right)\cdot \left(TN+FN\right)}}$$

where TP, TN, FP, and FN represent the count of correctly predicted positive samples, correctly predicted negative samples, incorrectly predicted positive samples, and incorrectly predicted negative samples, respectively.

*(3) Model training*

With only 63,872 parameters, MeSI is a simple and efficient model to differentiate metal-binding site and non-metal-binding sites. Considering the class imbalance in the dataset, we adopted the focal loss [19] as the loss function to train the model for 3,000 epochs. We employed Adam as the optimizer, where $\beta_{1}$ = 0.9, $\beta_{2}$ = 0.99, weight decay = 0, and the batch size is set to 256, with the default learning rate at 5e^−5^. If the model's loss on the validation set does not decrease within 200 epochs, a learning rate scheduler will automatically reduce the learning rate. Additionally, to avoid overfitting, training will be stopped if the loss on the validation set does not decrease within 1,000 epochs. Detailed hyperparameters are provided in the Supplementary Table S2. We trained the MeSI model using an Intel Xeon CPU E5–2680 v4 @2.40 GHz and one NVIDIA GeForce RTX 2080 Ti.

### 2.3 The MeSA module

MeSA, implemented in Python, utilizes the quadruple-matching algorithm from MeCOM to achieve 3D metal-binding site superposition. This algorithm aligns a query metal-binding site to a reference site based on their main atoms ($C_{\alpha }$, $C_{\beta }$, amide N, and carbonyl C), offering the flexibility to consider or disregard residue type and metal ion. The process begins by generating all possible four-atom combinations, termed “quadruples”, for both the query and reference sites. Each query quadruple is then iteratively superimposed onto the reference quadruples, ensuring agreement in main atoms ($C_{\alpha }$, $C_{\beta }$, amide N, and carbonyl C). The RMSD of the aligned atoms is calculated *via* the least-squares method. Finally, the optimal rotation matrix and translation vector, derived from the best quadruple match, are applied to align the entire query site onto the reference site. To assess the alignment quality and geometric similarity of the two sites, both RMSD and the AScore metric from MeCOM are utilized. MeSA primarily functions to compare MeSI-filtered residue triplets with known metal-binding sites, applying an RMSD cutoff of 0.4 Å to determine the most likely metal ions and/or coordination residue types, thereby facilitating both metal-binding site identification and grafting.

## 3 Results and discussion

### 3.1 The overview of MeSiteIG

**Fig. 2:**
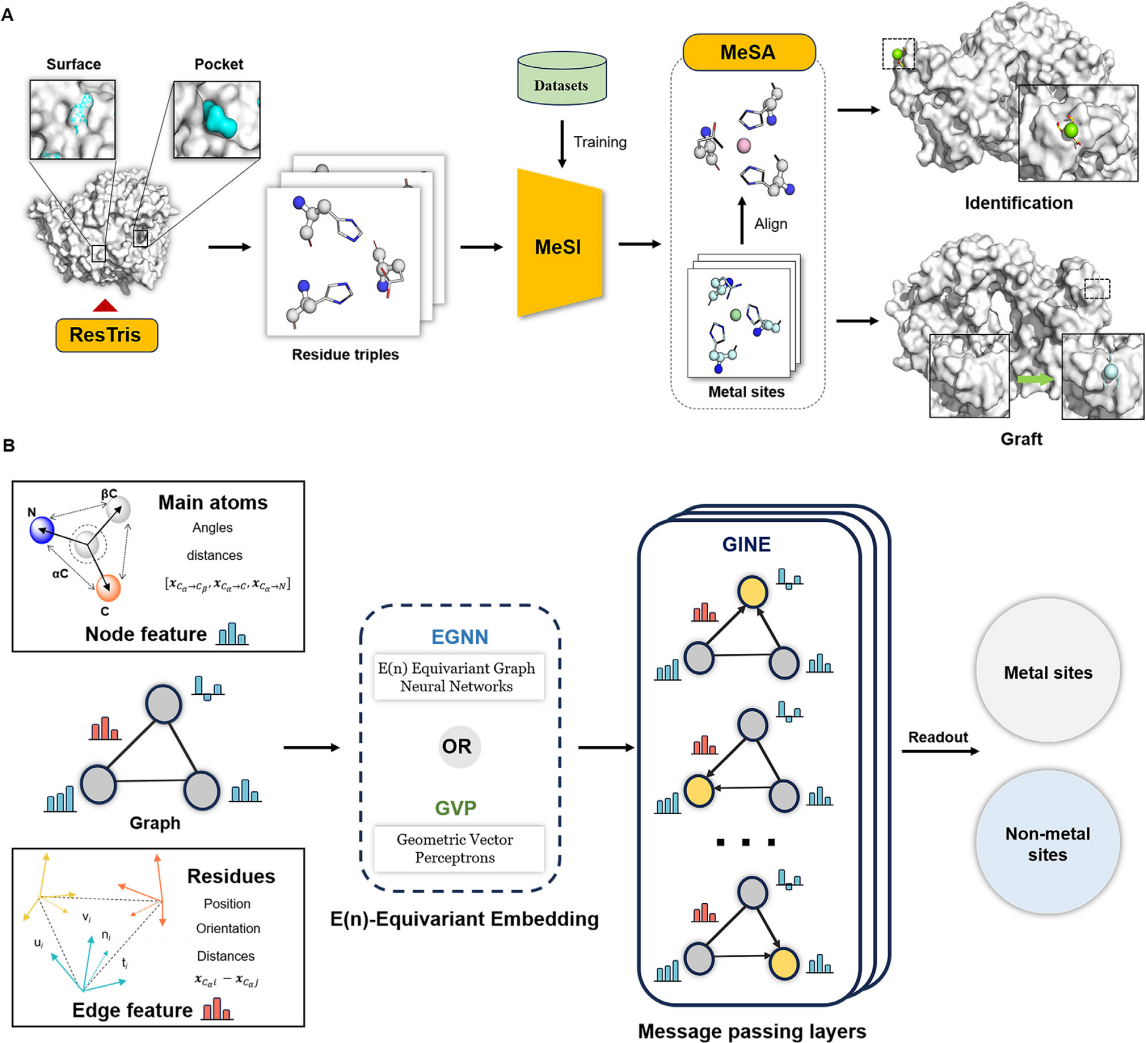
(A) MeSiteIG workflow. (B) MeSI network architecture.

MeSiteIG is mainly developed for both metal-binding site identification and grafting. It comprises three main modules: ResTriS, MeSI, and MeSA (Fig. 2A). Given a protein structure (PDB format), ResTriS searches for residue triplets within protein pockets (applying the fpocket algorithm [20]) or surfaces (applying the MSMS algorithm [21]), and passes them to the MeSI module for rapid identification of potential metal-binding site residue triplets based on their main atom information. The filtered residue triplets are then forwarded to the subsequent module, MeSA, which functions to complete metal ion(s) and/or metal ion-coordinating residue types by comparing them with known metal-binding sites. Since ResTriS typically generates thousands of residue triplets and the 3D superimposition in MeSA is relatively time-consuming, MeSI serves as the core engine of MeSiteIG, determining the overall efficiency and accuracy. MeSI is parameterized by an E3-equivariant graph neural network (GNN) to distinguish metal-binding site residue triplets from non-metal-binding site triplets, by learning on the spatial and geometric features of known metal-binding sites in the training dataset, relying solely on the information from their main atoms (including $C_{\alpha }$, $C_{\beta }$, amide N, and carbonyl C) (Fig. 2B).

To enrich the information of residue triplet represented by a graph G, we created informative node and edge features to enable the model to effectively capture the spatial and geometric properties (Fig. 1B). For each node, we utilized scalar features based on bond angles and distances between the main atoms within the residue, along with vector features representing $C_{\alpha }$-centered vectors pointing towards each remaining main atom. Edge features include scalar values encoding relative positions, orientations, and inter-residue distances, as well as vector features representing the $C_{\alpha }$ vectors between residues. This comprehensive representation compensates for the inherent information scarcity, providing the model with enough information for learning the discrepancy between metal-binding site and non-metal-binding site residue triplets. The MeSI module utilizes E3-equivariant graph neural networks (GNNs) to identify potential metal-binding site residue triplets in protein structures. By innovatively constructing node and edge features that incorporate rich spatial information, MeSI significantly enhances the model's accuracy in identifying metal-binding sites.

### 3.2 MeSI manifests superior performance in identifying metal-binding site residue triplets

We first evaluated the capabilities of MeSI model variants in identifying metal-binding site residue triplets on the independent test set. Three MeSI model variants: MeSI-base, MeSI-GVP, and MeSI-EGNN, were compared, which differ in whether they incorporate EGNN or GVP modules during message passing. Given the imbalanced positive-negative samples in this test set, we primarily focused on the recall metric, defined as the proportion of true positive samples correctly predicted as positive. We also considered the ROC-AUC, which provides a comprehensive assessment of the model's ability to discriminate between metal-binding site and non-metal-binding site residue triplets, and the PR-AUC metric, which can capture performance under imbalanced conditions, as it emphasizes the model's precision-recall balance.

As shown in Table 1, the MeSI-base model showed excellent predictive ability, with recall of 0.9781, ROC-AUC of 0.9851, and PR-AUC of 0.7864, suggesting that GINE is suitable for this task, likely due to its unique ability in merging information from nodes, their neighbors, and edges. Compared with MeSI-base, MeSI-EGNN manifested better performance with recall, ROC-AUC, and PR-AUC values of 0.9940, 0.9940, and 0.7997, respectively. MeSI-GVP also outperformed MeSI-base, with a recall of 0.9875 and ROC-AUC of 0.9919, but slightly lower PR-AUC of 0.6341, suggesting that while its overall discrimination ability remains strong, its precision-recall trade-off is less optimal compared to MeSI-EGNN. The superior performance of MeSI-EGNN may stem from EGNN's ability to explicitly model geometric relationships between nodes [22], allowing it to effectively identify the special spatial structures of residue triplets associated with metal-binding sites. In contrast, the GVP module, which considers vector features between nodes [16], may involve redundant or irrelevant information, preventing it from surpassing MeSI-EGNN which does not take vector features into account. In addition, EGNN's conciseness and focus on spatial information [23] allows it to effectively capture the graph information consisting solely of residue main atoms, leading to accurate prediction of metal-binding site residue triplets. Since MeSI is the first fundamental method for potential metal-binding site prediction based solely on the main atoms of the input residue triplets, we did not compare it with other reported metal-binding site prediction, which typically consider all atoms of the metal-coordination residues.

**Table 1:** **Performance of different MeSI variants on the independent test set**.

Model	Accuracy	Precision	Recall	MCC	ROC-AUC	PR-AUC
MeSI-base	0.9897	0.9609	0.9781	0.9633	0.9851	0.7864
MeSI-EGNN	0.9940	0.9432	0.9940	0.9650	0.9940	0.7997
MeSI-GVP	0.9949	0.9821	0.9875	0.9818	0.9919	0.6341

In terms of MeSI-EGNN's superior performance on the independent test set, we further evaluated its performance of retrieving potential metal sites in real-world application scenarios by combining it with two other modules, ResTriS and MeSA. We selected 20 different metalloenzymes for testing (Table 2), which were selected from the independent test set and possessed different metal ion-coordinating residue patterns. The residue triplets extracted from these 20 proteins by ResTriS were regarded as positive samples if the minimum RMSD was < 0.4 when aligning the known metal-binding site triplets to them with MeSA, and otherwise, treated as negative samples. MeSI exhibits outstanding performance in terms of both recall and ROC-AUC, suggesting that its predictions are highly consistent with those of MeSA. This indicates that MeSI can accurately and rapidly identify metal-binding sites in real-world scenarios. On this test set, MeSI shows rapid predictive speed with an average of 300 samples per second. With the comparable performance as MeSA, the speed and efficiency of MeSI make it a valuable pre-screening tool. Therefore, we propose utilizing MeSI to rapidly identify potential metal-binding sites, followed by a more precise evaluation with MeSA.

**Table 2:** **Performance of MeSI-EGNN on another test set consisting of 20 structurally and catalytically different metalloenzymes**.

Protein name	PDB code	Accuracy	Precision	Recall	MCC	ROC-AUC	PR-AUC	Positive sample number^a^	Sample number^b^
blaNDM-1	7UP1	0.9878	0.3478	0.9231	0.5626	0.9556	0.6289	26	3,837
ANCE	2X8Z	0.9913	0.5208	0.9368	0.6950	0.9643	0.7196	174	18,400
AVIN30970	6XB9	0.9884	0.4795	0.8750	0.6429	0.9323	0.6671	40	3707
PDE4D	7XAB	0.9895	0.5956	0.9759	0.7581	0.9828	0.7707	83	5,442
HDAC6	8A8Z	0.9908	0.5776	0.9710	0.7452	0.9811	0.7621	69	5,571
HDAC10	7SGK	0.9926	0.4670	0.9140	0.6505	0.9535	0.6842	93	14,180
MMP7	7WXX	0.9957	0.6667	0.8889	0.7678	0.9427	0.7705	18	2,337
ARG1	8E5M	0.9889	0.4337	0.8780	0.6128	0.9340	0.6477	41	4,700
ATU3453	4HCL	0.9900	0.3920	0.8846	0.5852	0.9376	0.6320	78	11,662
GLUL	2QC8	0.9911	0.3936	0.9024	0.5928	0.9470	0.6424	82	13,855
POL	3VQ5	0.9894	0.5806	0.9473	0.7372	0.9687	0.7500	38	2649
OPD	1JQM	0.9916	0.4032	0.9615	0.6198	0.9766	0.6768	52	9060
HYDA	4UCX	0.9928	0.4479	0.9203	0.6393	0.9568	0.6785	201	34,016
DEF	2AIE	0.9878	0.3444	0.8378	0.5327	0.9133	0.5848	37	5334
CDO1	4XFH	0.9915	0.2105	0.8000	0.4078	0.8960	0.5029	10	3784
HPD	5YWH	0.9899	0.3533	0.8030	0.5287	0.8970	0.5727	66	10,940
DR0930	2ZC1	0.9904	0.4237	0.9259	0.6228	0.9584	0.6679	54	7508
CA2	6lV1	0.9903	0.1326	0.8125	0.3259	0.9015	0.4710	16	9074
PHM	3MLL	0.9907	0.2330	0.7380	0.4116	0.8648	0.4826	42	12,229
PMOB1	3RGB	0.9909	0.4780	0.8445	0.6315	0.9183	0.6535	283	33,560

a The number of positive residue triplets labeled by MeSA.

b The number of all residue triplets (positive and negative residue triplets) labeled by MeSA.

### 3.3 MeSiteIG identifies the previously neglected metal-binding sites in many proteins

**Fig. 3:**
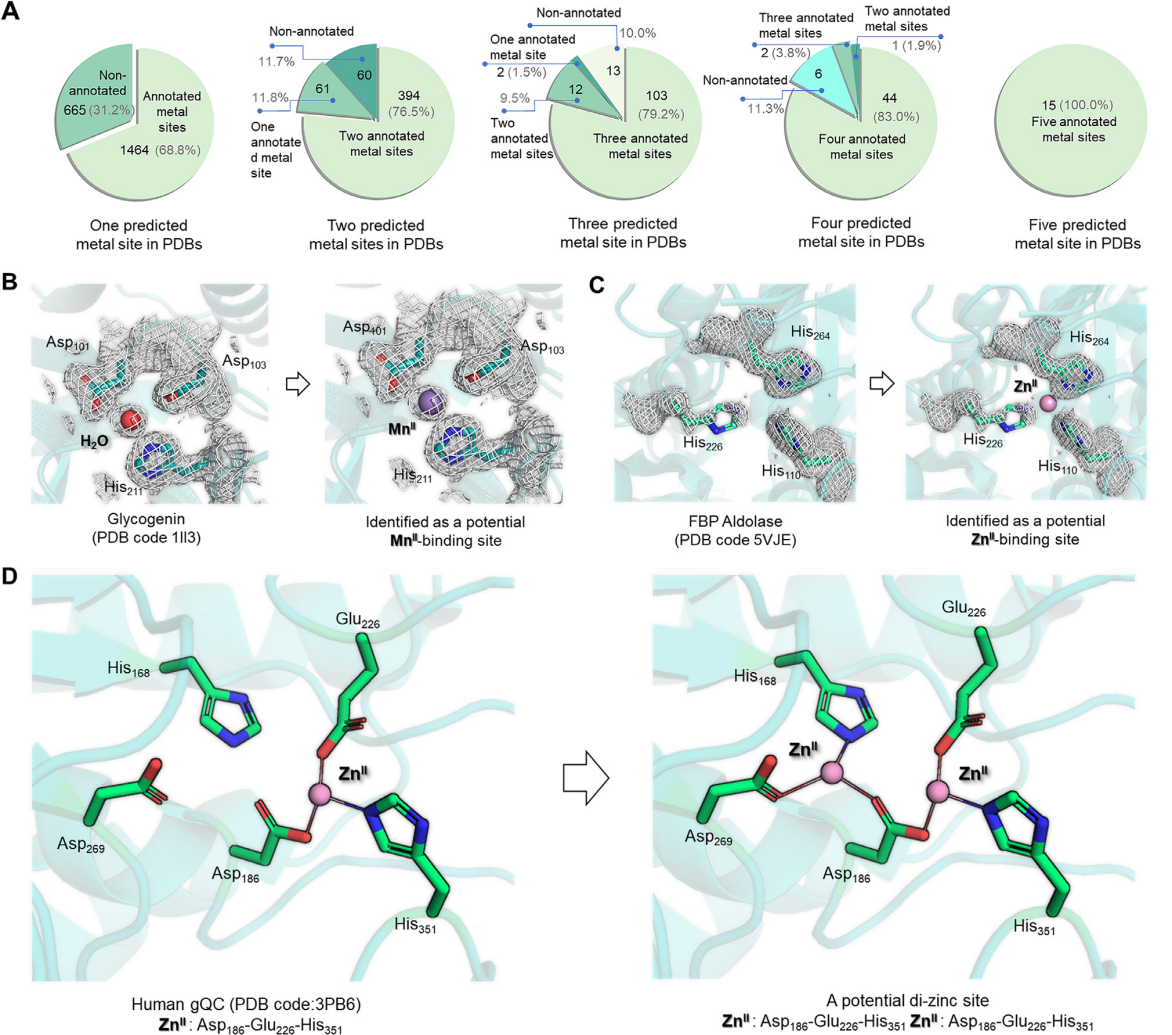
**MeSiteIG identifies neglected metal-binding sites.** (A) Pie chart showing the number of annotated/potential metal-binding sites. (B) The predicted Mn^II^ binding site occupied by a water molecule in the crystal structure. The left panel shows the original electron density maps of the predicted site in the Glycogenin structure (PDB code 1ll3) [28]. The right panel shows the refined electron density map of a Mn^II^ binding site. (C) The potential Zn^II^ binding site predicted by MeSiteIG, lacking corresponding electron density maps for Zn^II^. The left panel shows the original electron density map of the predicted site in the FBP Aldolase structure (PDB code 5VJE) [25]. The right panel shows the predicted Zn^II^ binding site with refined electron density maps. (D) Identification of additional Zn^II^ binding site in human gQC (PDB code 3PB6) [26].

To evaluate the capability of MeSiteIG to identify metal-binding sites, we analyzed 14,620 PDB files obtained from RCSB PDB [24], each corresponding to a protein with a molecular mass < 90 kDa. A total of 3,842 potential metal-binding sites across 2,843 analyzed PDB files were obtained by MeSiteIG prediction (Table S3). By querying the 2,129 (74.9%) PDB structures predicted to contain only one metal-binding site, we observed that in 1,464 of these structures, the predicted sites are indeed experimentally validated as metal-binding sites (Fig. 3A). A total of 515 PDB structures were identified by MeSiteIG to have two metal-binding sites (Fig. 3A). Notably, among all 3,842 predicted sites, 2,913 cases (75.8%) are annotated metal-binding sites, demonstrating MeSiteIG's proficiency in recognizing known metal-binding sites. Importantly, MeSiteIG identified additional 929 potential metal-binding sites that are not currently annotated metal-binding sites (Table S3).

To validate the predicted potential metal-binding sites, we examined the corresponding electron density maps in the respective PDB files (Table S4). In some cases where single metal-binding sites were predicted, we observed that the positions predicted as metal-binding sites were occupied by water molecules in the reported PDB structures. For example, the residue triplet Asp_101_-Asp_103—_His_211_ in the structure of glycogenin (PDB ID 1ll3) was predicted to potentially bind with Mn^II^ (Fig. 3B). By performing further refinements for this PDB structure using WinCoot and Phenix software, we found that the Asp_101_-Asp_103—_His_211_ site could bind with a Mn^II^ ion (Fig, 3B). This observation can be validated by other crystal structures of glycogenin (e.g. PDB code 1ZDG) [25].

We also observed some cases where there were no electron density maps for the predicted metal ion (Table S3). For example, in the structure of FBP Aldolase (PDB code 5VJE) [25], the His_110—_His_226—_His_264_ triplet was predicted to bind with Zn^II^, while no apparent electron density map was found (Fig. 3C). The absence of Zn^II^ in this crystal structure may due to the inappropriate protein production or crystallization conditions. In fact, within another structure of FBP Aldolase (PDB code 5VJD) [25], there has a clear electron density map for Zn^II^ binding, confirming the ability of MeSiteIG in identifying neglected metal-binding sites.

Beyond identifying mono-metal-binding sites, MeSiteIG also has ability to identify previously neglected bi-metal-binding sites (Table S5). By filtering out the predicted potential metal-binding sites that are spatially close to the known metal-binding sites, we identified several potential bi-metal-binding sites within the protein structures. One such instance was observed in human Golgi-resident glutaminyl cyclase (gQC) (PDB code 3PB6) [26]. In gQC, the Asp_186_-Asp_269—_His_168_ site, adjacent to the known Zn^II^: Asp_186_-Glu_226—_His_351_ site, was predicted to be a potential Zn^II^ binding site. This observation has been demonstrated that, under high zinc concentrations, human gQC can bind an additional Zn^II^ ion at this specific site, forming a bi-zinc metal site and altering gQC's activity [27]. These findings indicate that by discovering metal-binding sites, we can better understand the activity of metalloenzymes under different conditions, thereby gaining a comprehensive understanding of the profound implications of metal ion regulation of enzyme activity. In summary, MeSiteIG excels at identifying known and previously neglected metal-binding sites from experimentally determined or computationally predicted (e.g. AlphaFold) protein structures, deepening our understanding of metalloenzyme functions.

### 3.4 MeSiteIG enables metal-binding site grafting in different protein positions

**Fig. 4:**
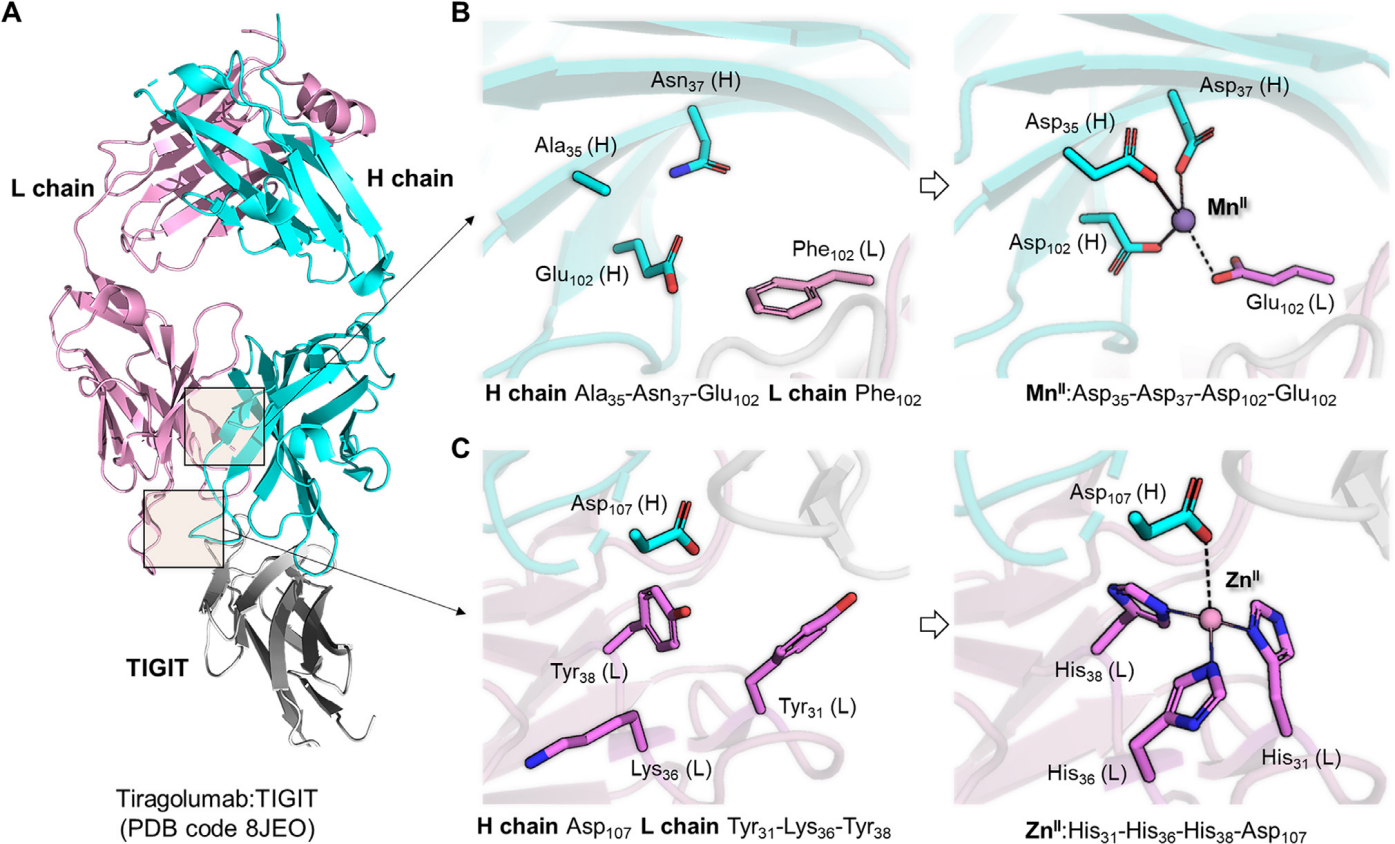
**MeSiteIG identifies two potential grafting sites on Tiragolumab.** (A) View of a crystal structure of Tiragolumab (PDB code 8JEO) [29], where the L chain is shown in purple, the H chain in cyan, and TIGIT in gray. (B) The first potential site in the H chain is predicted to enable grafting of a Mn^II^: Asp_35_-Asp_37_-Asp_102_ binding site. (C) The second potential grafting site in the L chain is predicted to enable grafting of a Zn^II^: His_31—_His_36—_His_38_ binding site.

MeSiteIG performs the metal-binding site grafting function by utilizing MeSI to predict potential metal-binding residue triplets, which are then compared with known metal-binding sites using MeSA. The highly matched residue triplets, along with their corresponding metal ions, are transferred to the target proteins, effectively achieving metal-binding site grafting. Since antibodies are central to a wide range of bio-therapeutic applications due to their high specificity and affinity for target molecules, we here used MeSiteIG to explore the possibility of engineering metal-binding sites, potentially enhancing their stability or introducing new functionalities. Tiragolumab, an antibody targeting TIGIT that can enhance antitumor activity by boosting the body's immune response against cancer cells, was selected because its high-quality crystal structure is available (PDB code 8JEO, Fig. 4A) [29]. By using MeSiteIG, we identified two potential sites for metal site grafting that may enhance the interactions between heavy (H) and light (L) chains.

The first potential grafting site, Ala_35_-Asn_37_-Glu_102_, is located in the H chain (Fig. 4B). This site can be replaced with a Mn^II^ binding site, Asp_35_-Asp_37_-Asp_102_. If Phe_102_ on the L chain is engineered to Glu_102_, the grafted Mn^II^ binding site may increase the affinity of H chain binding with L chain through additional coordination interactions (Fig. 4B). The second potential grafting site, Tyr_31_-Lys_36_-Tyr_38_, is in the L chain, which can be engineered with a Zn^II^: His_31—_His_36—_His_38_ site. Notably, the neighboring residue ASP_107_ on the H chain, with its carboxyl side chain, may electrostatically interact with or directly coordinate with the Zn^II^ ion (Fig. 4C), possibly increasing the stability of Tiragolumab through metal-binding site-mediated interactions. Additionally, this grafted site may also affect the binding of Tiragolumab with the target molecule TIGIT.

**Fig. 5:**
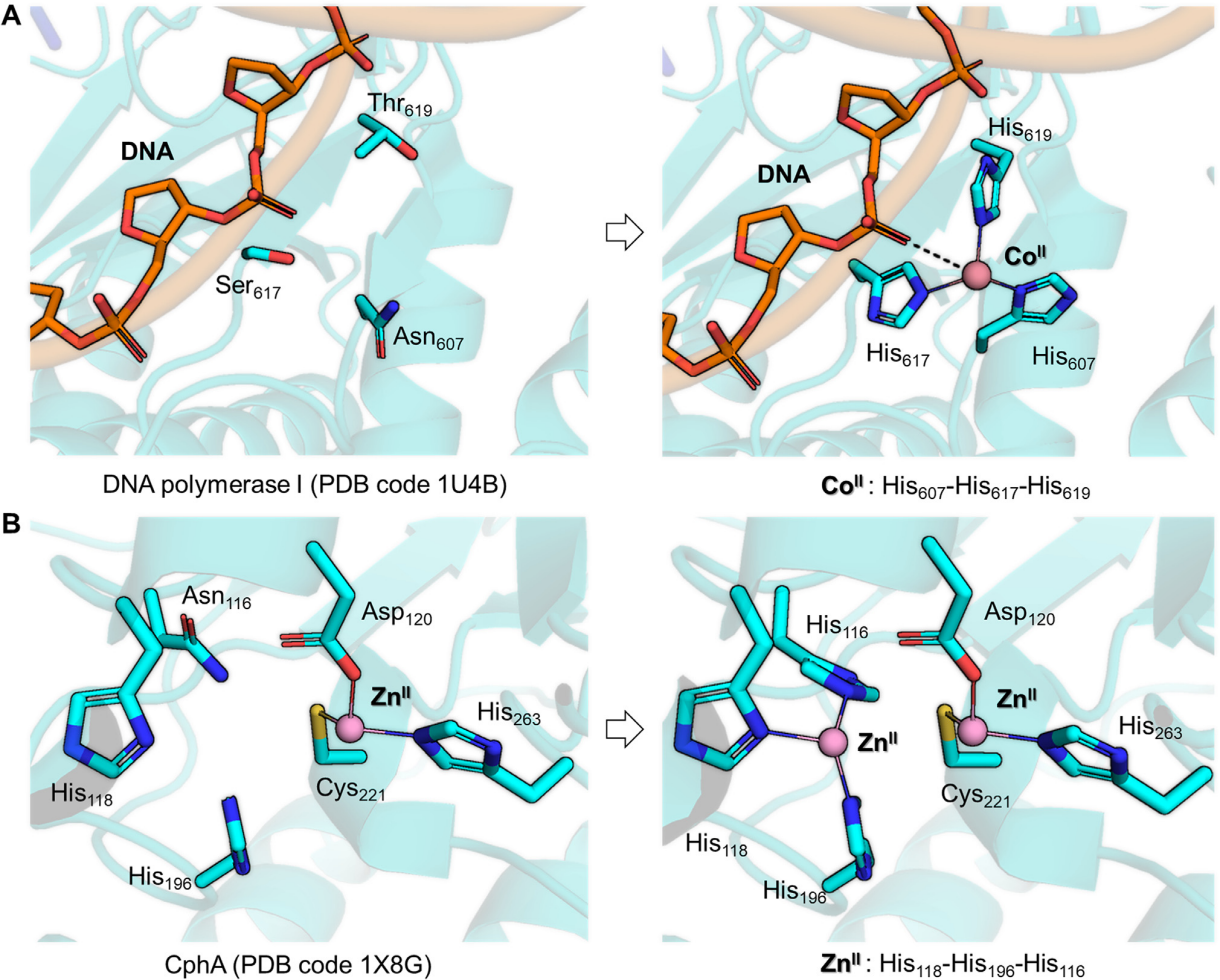
**MeSiteIG identifies potential functional sites for metal site grafting.** (A) MeSiteIG predicts a cobalt-binding site, His_617—_His_607—_His_619_, in DNA polymerase I (PDB ID 1U4B) [30] that can replace Ser_617_-Asn_607_-Thr_619_. (B) MeSiteIG predicts a zinc-binding site, His_116—_His_118—_His_196_, in CphA (PDB ID 1 × 8 G)[31] that only needs the Q116H mutation.

We next used MeSiteIG for metal site grafting in protein binding pockets. First, we selected DNA polymerase I, a crucial enzyme involved in DNA replication and repair, because it has a crystal structure of DNA polymerase I in complex with DNA (PDB ID 1U4B) [30]. MeSiteIG identified a potential grafting site, Ser_617_-Asn_607_-Thr_619_, on DNA polymerase I, which is adjacent to the DNA binding region (Fig. 5A). This site can be grafted with a Co^II^: His_617—_His_607—_His_619_ metal site, which lies spatially close to both the active site and the DNA backbone. The cobalt ion in the grafted site could potentially engage in coordination interactions with the DNA backbone (Fig. 5A), thereby influencing the binding stability of DNA polymerase I with DNA.

CphA, a mono-zinc metallo-β-lactamase (MBL) belonging to the B2 subclass, can hydrolyze β-lactam antibiotics, associated with antibiotic resistance. MeSiteIG identified a potential site, Asn_116—_His_118—_His_196_, which is near to a known Zn^II^ binding site (Fig. 5B). This site can be grafted with a new stable Zn^II^ binding site, His_116—_His_118—_His_196_ (Fig. 5B). Notably, although His_118_ and His_196_ residues were observed to bind with a second Zn^II^ ion that renders the enzyme inactive [31], this Zn^II^ site is unstable and requires a relatively high Zn^II^ concentration. In this case, the grafted site contains three histidine residues and has a stronger ability to bind with Zn^II^. The altered di-zinc B2 MBL has a similar di-zinc site with that of B1 di-zinc MBLs, possibly showing specific activity against β-lactam antibiotics.

These case studies reveal the ability of MeSiteIG to achieve metal site grafting for a given protein, whether on its surface or in a pocket. Coupled with protein engineering, MeSiteIG can offer several possibilities for metal site grafting, thereby expanding the potential applications of engineered metalloproteins.

## 4 Conclusion

MeSiteIG is the first tool for metal site identification and grafting that does not require the types of coordinating residues or side-chain information. It is established based on the geometric conservation of metal-binding sites, and integrates E3-equivarient GNN with a 3D superimposition algorithm. The metal-binding site identifier, MeSI, showed superior performance on the independent test set, with an impressive speed. MeSI determines the overall efficiency and accuracy of MeSiteIG. The case studies have demonstrated the power of MeSiteIG in metal site identification and grafting. The source codes and trained model of MeSiteIG are freely accessible at https://mesiteig.ddtmlab.org. It will be an important tool for expanding metal site informatics and their applications, especially in metalloenzyme-involved fields of chemistry, biology, and medicine.

## Code availability

The source codes of MeSiteIG are available at https://mesiteig.ddtmlab.org.

## Declaration of competing interests

The authors declare that they have no conflicts of interest in this work.

## Appendix Supplementary materials

Image, application 1
