# Supplementary material for: Geometric deep learning-enabled metal-binding site identification and grafting
Source: Fundam Res. 2024 Nov 29;6(4):2299–307. doi: 10.1016/j.fmre.2024.11.012 (PMC13424184; doi:10.1016/j.fmre.2024.11.012)
Supplement: Supplementary file 1 [file mmc1.docx]

***Supplementary Information***

**Geometric deep learning-enabled metal-binding site identification and grafting**

Jun-Lin Yu,^1,2^ Yao-Geng Wang,^1,2^ Jian Peng,^1^ Jing-Wei Wu,^1^ Cong Zhou,^1^ Guo-Bo Li^1,*^

^1^Key Laboratory of Drug-Targeting and Drug Delivery System of the Education Min-istry and Sichuan Province, Department of Medicinal Chemistry, West China School of Pharmacy, Sichuan University, Chengdu 610041, China

^2^J.-L.Y. and Y.-G.W. are co-first authors.

*Correspondence: [liguobo@scu.edu.cn](mailto:liguobo@scu.edu.cn) (G.-B.L.)

**Supplementary Tables**

**Table S1**. RMSD thresholds for non-metal site sample filtering.

| **Number of disturbed residues** | **Lower threshold(Å)** | **Upper threshold(Å)** |
| --- | --- | --- |
| **1** | 0.4 | 0.8 |
| **2** | 0.5 | 1 |
| **3** | 0.5 | 1.1 |

**Table S2**. MeSI model training hyperparameters

| **Hyperparameter** | **Value** |
| --- | --- |
| **Epochs** | 3000 |
| **Batch Size** | 256 |
| **Learning Rate (lr)** | 0.00005 |
| **Alpha** | 0.8 |
| **Gamma** | 2 |
| **Early stopping patience** | 1000 |
| **Hidden layer dimensions** | [128, 64, 32] |

**Table S3**. Identification of annotated and previously neglected metal sites by MeSiteIG.

| **PDB code** | **UniProt ID** | **Number of predicted metal sites** | **Number of annotated metal sites** |  | **PDB code** | **UniProt ID** | **Number of predicted metal sites** | **Number of annotated metal sites** |
| --- | --- | --- | --- | --- | --- | --- | --- | --- |
| 4NPL | Q08BA6 | 1 | 1 |  | 3PT1 | Q04371 | 1 | 1 |
| 3LKA | P39900 | 1 | 1 |  | 3PUA | O75151 | 1 | 1 |
| 5MEE | B7JX99 | 1 | 1 |  | 3PXL | B2L9C1 | 1 | 1 |
| 1MZC | P49354 | 1 | 0 |  | 3Q31 | Q2TWF5 | 1 | 1 |
| 4GKV | P39451 | 1 | 1 |  | 3Q46 | H2L2L6 | 1 | 1 |
| 3R5V | H0USY5 | 1 | 1 |  | 3Q5I | A0A509AHB6 | 1 | 1 |
| 3ERN | P62617 | 1 | 1 |  | 3Q8H | Q63T71 | 1 | 1 |
| 5NEL | Q81EJ6 | 1 | 1 |  | 3Q94 | A0A6H3AA11 | 1 | 1 |
| 5QKB | Q9UKK9 | 1 | 1 |  | 3LNP | D3KFX9 | 1 | 1 |
| 1JAP | P22894 | 1 | 1 |  | 3D4U | Q2KIG3 | 1 | 1 |
| 1MMR | P09237 | 1 | 1 |  | 3LLU | Q9HB90 | 1 | 1 |
| 7N0Z | Q9ULR3 | 1 | 1 |  | 3LLM | Q08211 | 1 | 1 |
| 2W1A | O53512 | 1 | 1 |  | 3EYX | P53615 | 1 | 1 |
| 2E6C | Q53W92 | 1 | 1 |  | 3FE2 | P17844 | 1 | 0 |
| 7CBG | V7II86 | 1 | 1 |  | 3FE4 | P23280 | 1 | 1 |
| 4UCG | Q9K169 | 1 | 1 |  | 3FHR | Q16644 | 1 | 0 |
| 6CZE | P9WPQ5 | 1 | 1 |  | 3FJU | P15085 | 1 | 1 |
| 5TUO | A0A0M3KL20 | 1 | 1 |  | 8A28 | B4F320 | 1 | 1 |
| 1IV2 | Q8RQP5 | 1 | 1 |  | 3EXM | Q9FBN7 | 1 | 1 |
| 7S77 | P36871 | 1 | 0 |  | 3G4E | Q15493 | 1 | 1 |
| 3NZP | Q0PAC6 | 1 | 0 |  | 3GC2 | P58486 | 1 | 0 |
| 8E4X | P78563 | 1 | 1 |  | 3GD6 | Q8EMJ9 | 1 | 0 |
| 8OU8 | P0A8M3 | 1 | 1 |  | 3GHZ | Q8ZMF7 | 1 | 1 |
| 5BKE | Q89UC4 | 1 | 1 |  | 3GQM | Q63KH5 | 1 | 0 |
| 4FIP | P50102 | 1 | 0 |  | 3GRN | Q8PYE2 | 1 | 0 |
| 6HQY | A5H660 | 1 | 1 |  | 3GVF | Q3JUV5 | 1 | 0 |
| 7MYJ | P54646 | 1 | 0 |  | 3GBO | P85314 | 1 | 1 |
| 4H2Y | Q89VT8 | 1 | 1 |  | 3GVY | Q3J696 | 1 | 0 |
| 1RV8 | Q9RHA2 | 1 | 1 |  | 3EN1 | A5W4F2 | 1 | 1 |
| 5FGJ | P04176 | 1 | 1 |  | 3EEF | Q9HKY9 | 1 | 1 |
| 6NB2 | Q5ZTX1 | 1 | 1 |  | 3DGB | Q4K9X1 | 1 | 1 |
| 5KX6 | Q9SWH5 | 1 | 0 |  | 3DH1 | Q7Z6V5 | 1 | 1 |
| 7KCQ | S5RZC2 | 1 | 1 |  | 3DIV | D0VWU3 | 1 | 1 |
| 4XZE | P27318 | 1 | 0 |  | 3DKQ | A3D8P6 | 1 | 1 |
| 8K6Y | Q5SJ79 | 1 | 1 |  | 3DMO | Q3JJN0 | 1 | 1 |
| 5MYV | P78362 | 1 | 0 |  | 3DTE | C1CZ84 | 1 | 0 |
| 1SJD | Q44244 | 1 | 0 |  | 3EGJ | O32445 | 1 | 1 |
| 2J59 | P84078 | 1 | 1 |  | 3DUW | Q739U3 | 1 | 0 |
| 5YGR | P40817 | 1 | 0 |  | 3DX5 | Q81RQ4 | 1 | 1 |
| 3C66 | P29468 | 1 | 0 |  | 3DZB | Q5M554 | 1 | 0 |
| 4FLY | P0CL77 | 1 | 1 |  | 3E2C | P0ABD3 | 1 | 1 |
| 1H1M | Q7SIC2 | 1 | 1 |  | 3EDH | P13497 | 1 | 1 |
| 1AYK | P03956 | 1 | 1 |  | 3EDI | O43897 | 1 | 1 |
| 2IHM | Q9JIW4 | 1 | 0 |  | 3EE4 | P9WH69 | 1 | 1 |
| 4EIW | Q5SI82 | 1 | 0 |  | 3DWB | P42892 | 1 | 1 |
| 5JGF | P14904 | 1 | 1 |  | 3GZX | Q46372 | 1 | 1 |
| 2QB6 | P38698 | 1 | 1 |  | 3H08 | Q93SU7 | 1 | 1 |
| 8A0B | Q92769 | 1 | 1 |  | 3H4X | B3A043 | 1 | 0 |
| 7CL5 | Q6L732 | 1 | 1 |  | 3K7N | D3TTC1 | 1 | 1 |
| 6S6D | Q7L523 | 1 | 1 |  | 3KT7 | P40032 | 1 | 1 |
| 1FM1 | P45452 | 1 | 1 |  | 3KTC | Q6D5T7 | 1 | 1 |
| 1DV1 | P24182 | 1 | 0 |  | 3KV9 | Q6ZMT4 | 1 | 1 |
| 3WQQ | O96693 | 1 | 1 |  | 3KZN | Q8P8J2 | 1 | 0 |
| 5QP3 | Q8IU60 | 1 | 0 |  | 3L5K | Q08623 | 1 | 0 |
| 8D9D | P49642 | 1 | 0 |  | 3K7I | Q14623 | 1 | 1 |
| 4YTU | O50580 | 1 | 1 |  | 3L6T | Q9X4G2 | 1 | 1 |
| 2C47 | P78368 | 1 | 0 |  | 3LAS | Q8DVY1 | 1 | 1 |
| 7YNH | P46881 | 1 | 1 |  | 3LAT | O33635 | 1 | 1 |
| 3V11 | Q980A5 | 1 | 0 |  | 3LD3 | Q2GJ02 | 1 | 0 |
| 6KAJ | Q9H9S5 | 1 | 1 |  | 3LFU | P03018 | 1 | 0 |
| 1KRQ | Q46106 | 1 | 0 |  | 3LHO | Q07XY2 | 1 | 1 |
| 6NBA | P32929 | 1 | 0 |  | 3LIJ | Q5CS01 | 1 | 1 |
| 3IVU | Q9Y823 | 1 | 1 |  | 3L87 | Q8DWC2 | 1 | 1 |
| 3UXI | Q9KCL9 | 1 | 1 |  | 3K5T | P19801 | 1 | 1 |
| 5NJA | P0AGG8 | 1 | 1 |  | 3K3O | Q9UPP1 | 1 | 1 |
| 1DE6 | P32170 | 1 | 1 |  | 3K2O | Q6NYC1 | 1 | 1 |
| 8P7N | P0A434 | 1 | 1 |  | 3H56 | P38501 | 1 | 1 |
| 6KNF | P25006 | 1 | 1 |  | 3H70 | Q53635 | 1 | 1 |
| 7E3U | Q9I676 | 1 | 1 |  | 3H7C | Q8GWW7 | 1 | 0 |
| 6D3M | Q8KSC8 | 1 | 1 |  | 3HJ4 | C7AJA4 | 1 | 0 |
| 4CVS | P0ABD3 | 1 | 1 |  | 3HMB | P39800 | 1 | 1 |
| 8E0Z | P0AB91 | 1 | 0 |  | 3HPO | D9N168 | 1 | 0 |
| 4M2E | G9MBV2 | 1 | 0 |  | 3HYG | Q9UNA0 | 1 | 1 |
| 7BBJ | P21589 | 1 | 1 |  | 3HYH | P06782 | 1 | 0 |
| 4PN1 | Q9P6Q6 | 1 | 0 |  | 3IFE | Q81WU4 | 1 | 1 |
| 6VK7 | P27353 | 1 | 1 |  | 3IIE | B1JQG4 | 1 | 1 |
| 5NA6 | Q8A6N1 | 1 | 1 |  | 3IOG | P26918 | 1 | 1 |
| 5NFO | P0C870 | 1 | 1 |  | 3ISQ | P32754 | 1 | 1 |
| 5NG0 | O43353 | 1 | 0 |  | 3IT5 | P14789 | 1 | 1 |
| 5NQ7 | D7F485 | 1 | 1 |  | 3IUZ | Q46PQ5 | 1 | 0 |
| 5NX7 | B5GMG2 | 1 | 1 |  | 3JRQ | P49597 | 1 | 0 |
| 5O9W | D4N500 | 1 | 1 |  | 3LLT | Q8IL19 | 1 | 0 |
| 5OGJ | Q8N1Q1 | 1 | 1 |  | 3SDB | P9WJJ3 | 1 | 0 |
| 5P9V | P22734 | 1 | 1 |  | 2NS6 | D0VWX2 | 1 | 1 |
| 5QHH | P0C024 | 1 | 0 |  | 2J43 | Q8KLP1 | 1 | 0 |
| 5QI5 | Q460N5 | 1 | 0 |  | 1NU5 | P27099 | 1 | 1 |
| 5R7X | Q7LBC6 | 1 | 1 |  | 1O1Z | Q9X1V6 | 1 | 0 |
| 5SYT | O75844 | 1 | 1 |  | 1O2D | Q9X022 | 1 | 1 |
| 5SZJ | P61026 | 1 | 1 |  | 1O3Y | P84078 | 1 | 1 |
| 5TC9 | G0RVK1 | 1 | 0 |  | 1O6L | P31751 | 1 | 1 |
| 5TD3 | A4JR51 | 1 | 1 |  | 1O7N | P0A110 | 1 | 1 |
| 5N3J | P25321 | 1 | 0 |  | 1NQZ | Q9RV46 | 1 | 1 |
| 5TJ3 | Q9KJX5 | 1 | 1 |  | 1ODM | P05326 | 1 | 1 |
| 5MZG | P53368 | 1 | 0 |  | 1OGL | O15923 | 1 | 0 |
| 5MRV | Q8IVL8 | 1 | 1 |  | 1OHT | Q8INK6 | 1 | 1 |
| 5JOH | Q92905 | 1 | 1 |  | 1OIK | Q9WWU5 | 1 | 1 |
| 5JZJ | O15075 | 1 | 1 |  | 1OIX | P62491 | 1 | 1 |
| 5K53 | A0A1D5B390 | 1 | 1 |  | 1ONW | P39377 | 1 | 1 |
| 5K8C | Q8EEB0 | 1 | 1 |  | 1OQ9 | P22337 | 1 | 1 |
| 5K8K | P44046 | 1 | 1 |  | 1OE1 | O68601 | 1 | 1 |
| 5KIA | Q2T9E1 | 1 | 0 |  | 1P6B | P0A433 | 1 | 1 |
| 5KIV | P67344 | 1 | 1 |  | 1NO5 | P43933 | 1 | 1 |
| 5KRY | Q8DBF5 | 1 | 0 |  | 1MZY | Q53239 | 1 | 1 |
| 5KSQ | Q9PF20 | 1 | 1 |  | 1L6S | P0ACB2 | 1 | 1 |
| 5L03 | A0A069B2G5 | 1 | 1 |  | 1LBU | P00733 | 1 | 1 |
| 5L12 | A3NWD9 | 1 | 1 |  | 1LFW | P45494 | 1 | 1 |
| 5LD9 | Q8U1Y4 | 1 | 1 |  | 1LGT | P47228 | 1 | 1 |
| 5LF8 | P0C025 | 1 | 0 |  | 1LL3 | P13280 | 1 | 0 |
| 5LN5 | O94580 | 1 | 1 |  | 1LM6 | Q9F2F0 | 1 | 1 |
| 5LS4 | Q5S581 | 1 | 1 |  | 1NJG | P06710 | 1 | 0 |
| 5MYI | A0A2D0TC88 | 1 | 1 |  | 1LME | P96113 | 1 | 0 |
| 2VES | P47205 | 1 | 1 |  | 1LOX | P12530 | 1 | 1 |
| 5U4N | Q5FAI4 | 1 | 0 |  | 1M4L | P00730 | 1 | 1 |
| 5UCQ | H0USY5 | 1 | 1 |  | 1MH1 | P63000 | 1 | 1 |
| 3KHL | Q97W02 | 1 | 1 |  | 1MQ0 | P32320 | 1 | 1 |
| 3UNY | Q818Z9 | 1 | 1 |  | 1MUC | P08310 | 1 | 1 |
| 5JBI | Q8IKG4 | 1 | 1 |  | 1MXR | P69924 | 1 | 1 |
| 2V4O | P66881 | 1 | 1 |  | 1LML | P08148 | 1 | 1 |
| 1XVD | P22869 | 1 | 1 |  | 1PHK | P00518 | 1 | 1 |
| 8FG4 | P01112 | 1 | 1 |  | 1PHZ | P04176 | 1 | 1 |
| 2WFR | O43323 | 1 | 1 |  | 1PIW | Q04894 | 1 | 1 |
| 3KAL | Q9M426 | 1 | 1 |  | 1R8G | P77213 | 1 | 0 |
| 2L47 | Q8LTE6 | 1 | 1 |  | 1RA0 | P25524 | 1 | 1 |
| 6W2K | Q72HW2 | 1 | 1 |  | 1RDQ | P05132 | 1 | 1 |
| 4MGG | A0NXQ8 | 1 | 1 |  | 1RJ5 | Q9WVT6 | 1 | 1 |
| 1D1S | P40394 | 1 | 1 |  | 1RQB | Q70AC7 | 1 | 1 |
| 6N1K | P00439 | 1 | 1 |  | 1RV9 | Q9K0A8 | 1 | 0 |
| 8HUG | P62826 | 1 | 1 |  | 1R6W | P29208 | 1 | 1 |
| 7E51 | P9WNL1 | 1 | 1 |  | 1RW0 | Q8Z4J1 | 1 | 0 |
| 4Q44 | Q47155 | 1 | 1 |  | 1SDY | P00445 | 1 | 1 |
| 5U55 | Q9JN69 | 1 | 1 |  | 1SG6 | P07547 | 1 | 1 |
| 1HFB | P32449 | 1 | 0 |  | 1SRP | P07268 | 1 | 1 |
| 5VXT | B1Z4S0 | 1 | 1 |  | 1SVS | P10824 | 1 | 1 |
| 5UEJ | Q9JYL2 | 1 | 1 |  | 1SYY | O84835 | 1 | 1 |
| 5UGR | C5B113 | 1 | 1 |  | 1T0A | Q8EBR3 | 1 | 1 |
| 5UH7 | P9WGY7 | 1 | 0 |  | 1RXQ | O31562 | 1 | 1 |
| 5UMH | A0A0H3KXJ8 | 1 | 1 |  | 1R5T | Q06549 | 1 | 1 |
| 5UQD | Q9GRZ3 | 1 | 1 |  | 1R55 | Q9BZ11 | 1 | 1 |
| 5UWZ | Q7NGM3 | 1 | 0 |  | 1R42 | Q9BYF1 | 1 | 1 |
| 5UX2 | A3Z6M0 | 1 | 1 |  | 1PJR | P56255 | 1 | 0 |
| 5UXI | B2J1M1 | 1 | 0 |  | 1PMI | P34948 | 1 | 1 |
| 5V1B | Q96KS0 | 1 | 1 |  | 1PZS | P9WGE9 | 1 | 1 |
| 5VE3 | B2TEQ2 | 1 | 1 |  | 1Q0H | P45568 | 1 | 0 |
| 5VGM | Q9KL24 | 1 | 1 |  | 1Q0O | Q45135 | 1 | 1 |
| 5VJE | P0AB71 | 1 | 0 |  | 1Q3A | P09238 | 1 | 1 |
| 5VM2 | P77280 | 1 | 1 |  | 1Q79 | P25500 | 1 | 1 |
| 5VN6 | B1YQF4 | 1 | 1 |  | 1Q8Y | Q03656 | 1 | 1 |
| 5VRK | Q97VT7 | 1 | 1 |  | 1QHW | P29288 | 1 | 1 |
| 5W45 | Q6NTF7 | 1 | 1 |  | 1QR0 | P39135 | 1 | 1 |
| 4KT2 | Q1QT89 | 1 | 1 |  | 1QUA | P60244 | 1 | 1 |
| 1JC5 | Q8VQN0 | 1 | 0 |  | 1QWR | P39841 | 1 | 1 |
| 6R4V | O95749 | 1 | 1 |  | 1QWY | O33599 | 1 | 1 |
| 6DWO | Q832K9 | 1 | 1 |  | 1R2F | P17424 | 1 | 1 |
| 4D8F | O84835 | 1 | 1 |  | 1R2Q | P20339 | 1 | 1 |
| 3WIA | Q5L1X8 | 1 | 1 |  | 1L5X | Q8ZU79 | 1 | 0 |
| 6IE2 | Q13686 | 1 | 1 |  | 1T47 | Q53586 | 1 | 1 |
| 2DFV | O58389 | 1 | 0 |  | 1KW6 | P17297 | 1 | 1 |
| 4MPO | A0A0H3MCJ9 | 1 | 1 |  | 1KTG | Q9U2M7 | 1 | 1 |
| 3GKE | Q5S3I3 | 1 | 1 |  | 1E4E | P25051 | 1 | 1 |
| 3R2R | Q9HWF9 | 1 | 1 |  | 1E5R | O09345 | 1 | 0 |
| 6BH8 | O75844 | 1 | 1 |  | 1E9G | P00817 | 1 | 1 |
| 3PVT | P76077 | 1 | 0 |  | 1EHI | Q03ZI1 | 1 | 1 |
| 5LP3 | P39377 | 1 | 0 |  | 1EKE | Q57599 | 1 | 0 |
| 5IN3 | P07902 | 1 | 1 |  | 1F1U | Q44048 | 1 | 1 |
| 6EVE | Q03034 | 1 | 1 |  | 1E4C | P0AB87 | 1 | 1 |
| 3E74 | P77671 | 1 | 1 |  | 1F5N | P32455 | 1 | 1 |
| 4TTV | P26639 | 1 | 1 |  | 1F9C | Q51958 | 1 | 1 |
| 6KI7 | A0A0A7XPH7 | 1 | 0 |  | 1FBL | P21692 | 1 | 1 |
| 4KEV | Q97VT7 | 1 | 1 |  | 1FLJ | P14141 | 1 | 1 |
| 7BMM | Q9RY20 | 1 | 0 |  | 1FNO | P26311 | 1 | 1 |
| 3PFO | Q6N7D3 | 1 | 1 |  | 1FSJ | P09883 | 1 | 1 |
| 1PJ1 | P69924 | 1 | 1 |  | 1FTH | P0A2W6 | 1 | 0 |
| 3A2C | P49137 | 1 | 0 |  | 1F8F | Q59096 | 1 | 1 |
| 2AES | P15291 | 1 | 1 |  | 1G0H | Q57573 | 1 | 1 |
| 3ZUS | O00161 | 1 | 1 |  | 1DS1 | Q05581 | 1 | 1 |
| 5IPZ | P22748 | 1 | 1 |  | 1DI1 | Q03471 | 1 | 0 |
| 5AEW | P37333 | 1 | 1 |  | 1AK0 | P24289 | 1 | 1 |
| 7CFW | Q9I3B1 | 1 | 1 |  | 1ATL | P15167 | 1 | 1 |
| 5MYD | A0A2D0TC86 | 1 | 1 |  | 1BG7 | P07229 | 1 | 0 |
| 4E4H | Q9H6W3 | 1 | 1 |  | 1BQG | P42206 | 1 | 0 |
| 4F4Z | Q4JB80 | 1 | 1 |  | 1BUD | Q9PW35 | 1 | 1 |
| 3CP8 | Q8KA85 | 1 | 0 |  | 1BYI | P13000 | 1 | 0 |
| 2WO0 | P46883 | 1 | 1 |  | 1DMH | P07773 | 1 | 1 |
| 2E46 | Q08J22 | 1 | 1 |  | 1C1Y | P62834 | 1 | 1 |
| 4E80 | O13833 | 1 | 0 |  | 1CDO | P26325 | 1 | 1 |
| 6ICO | H9N289 | 1 | 1 |  | 1CGF | P03956 | 1 | 1 |
| 3I3W | Q5NII8 | 1 | 1 |  | 1CTT | P0ABF6 | 1 | 1 |
| 7OGP | L7T138 | 1 | 0 |  | 1CXV | P33435 | 1 | 1 |
| 3WCE | Q4CWB4 | 1 | 0 |  | 1D5A | Q7SIG7 | 1 | 1 |
| 4D0Z | Q10471 | 1 | 1 |  | 1DCS | P18548 | 1 | 0 |
| 5DT5 | K0A8J9 | 1 | 0 |  | 1CDK | P36887 | 1 | 1 |
| 4C22 | Q97N97 | 1 | 1 |  | 1G9K | O69771 | 1 | 1 |
| 8ESV | O14672 | 1 | 1 |  | 1GA8 | Q93EK7 | 1 | 1 |
| 3BQ5 | Q9X112 | 1 | 1 |  | 1GE7 | P81054 | 1 | 1 |
| 8BTT | Q9Y3I0 | 1 | 1 |  | 1JLN | Q62132 | 1 | 0 |
| 4L0O | P56069 | 1 | 0 |  | 1JN1 | P44815 | 1 | 1 |
| 6G3U | Q9I0L4 | 1 | 0 |  | 1JP4 | Q9Z1N4 | 1 | 1 |
| 6ZN4 | Q6MM14 | 1 | 1 |  | 1JQ5 | P32816 | 1 | 1 |
| 5IUF | O34600 | 1 | 0 |  | 1JQG | O97389 | 1 | 1 |
| 1ULI | Q53122 | 1 | 1 |  | 1JVB | P39462 | 1 | 1 |
| 4O6H | P09992 | 1 | 0 |  | 1JK0 | P09938 | 1 | 1 |
| 2P0I | D0VX14 | 1 | 0 |  | 1K2F | P61092 | 1 | 1 |
| 8ECC | Q8C6L5 | 1 | 0 |  | 1K8T | P40136 | 1 | 1 |
| 5DUL | Q8ZH62 | 1 | 0 |  | 1KA2 | Q8U3L0 | 1 | 1 |
| 3T6C | D4GJ14 | 1 | 1 |  | 1KAP | Q03023 | 1 | 1 |
| 1MZZ | Q53239 | 1 | 1 |  | 1KEQ | P23589 | 1 | 1 |
| 4QDF | F1CMY8 | 1 | 1 |  | 1KOP | Q50940 | 1 | 1 |
| 7ZSC | O15460 | 1 | 0 |  | 1KQ3 | Q9WYQ4 | 1 | 1 |
| 3FVL | P00730 | 1 | 1 |  | 1K7I | P16317 | 1 | 1 |
| 4KJD | P15693 | 1 | 0 |  | 1JD4 | Q24306 | 1 | 1 |
| 3NIP | Q9I6K2 | 1 | 0 |  | 1JD0 | O43570 | 1 | 1 |
| 6LVE | I6NT79 | 1 | 0 |  | 1JC4 | Q8VQN0 | 1 | 0 |
| 4H2H | Q0FPQ4 | 1 | 1 |  | 1GP6 | Q96323 | 1 | 1 |
| 7WPY | A0A097ZPD5 | 1 | 1 |  | 1GVF | P0AB74 | 1 | 1 |
| 1XW4 | Q9BYN0 | 1 | 0 |  | 1GX1 | P62617 | 1 | 1 |
| 7K74 | B2FU10 | 1 | 0 |  | 1H8L | P83852 | 1 | 1 |
| 1OQ7 | P22337 | 1 | 0 |  | 1HXP | P09148 | 1 | 1 |
| 4OOE | P9WNS1 | 1 | 1 |  | 1HY7 | P08254 | 1 | 1 |
| 4BJR | Q8NQE0 | 1 | 1 |  | 1I39 | O29634 | 1 | 0 |
| 6G33 | P49759 | 1 | 0 |  | 1I6P | P61517 | 1 | 1 |
| 4X8D | G7CFI3 | 1 | 1 |  | 1I88 | P30074 | 1 | 0 |
| 2VPQ | A0A0H3JRR2 | 1 | 1 |  | 1IK6 | Q8ZUR7 | 1 | 0 |
| 1KKR | O66145 | 1 | 1 |  | 1IM4 | P96022 | 1 | 0 |
| 3I6E | Q5LM96 | 1 | 1 |  | 1IM5 | O58727 | 1 | 1 |
| 3SP1 | O51545 | 1 | 1 |  | 1J3Q | P84140 | 1 | 1 |
| 5JM9 | P14904 | 1 | 0 |  | 1J8U | P00439 | 1 | 1 |
| 6E4R | Q8MRC9 | 1 | 0 |  | 1J9L | P96112 | 1 | 1 |
| 4N71 | D0E8I5 | 1 | 1 |  | 1KUF | O57413 | 1 | 1 |
| 4YFU | E1C9K5 | 1 | 0 |  | 2JGN | O00571 | 1 | 0 |
| 2ZE8 | P58758 | 1 | 0 |  | 1TE2 | P77247 | 1 | 1 |
| 5FLJ | A2VA43 | 1 | 1 |  | 1TQ5 | P46852 | 1 | 1 |
| 3MP4 | P35914 | 1 | 0 |  | 2D30 | A0A6L7GZQ8 | 1 | 1 |
| 3E2W | P45148 | 1 | 1 |  | 2D5R | Q9UIV1 | 1 | 0 |
| 6Z6H | P53973 | 1 | 1 |  | 2D7I | Q86SR1 | 1 | 1 |
| 2YWB | Q5SI28 | 1 | 0 |  | 2D8A | O58389 | 1 | 1 |
| 8Q1A | Q16790 | 1 | 1 |  | 2DDF | P78536 | 1 | 1 |
| 2USN | P08254 | 1 | 1 |  | 2DDK | O14732 | 1 | 0 |
| 3SU8 | P63000 | 1 | 1 |  | 2D1F | P9WG59 | 1 | 0 |
| 3JYG | Q7MR56 | 1 | 1 |  | 2DI4 | O67077 | 1 | 0 |
| 5EBB | Q92484 | 1 | 1 |  | 2E3X | Q7LZ61 | 1 | 1 |
| 1U2D | Q2RSB2 | 1 | 0 |  | 2E47 | Q08J22 | 1 | 1 |
| 5CU9 | Q5AD07 | 1 | 0 |  | 2E6M | O09053 | 1 | 0 |
| 6NPA | A0A4V8H040 | 1 | 1 |  | 2E8V | Q12051 | 1 | 0 |
| 3RO6 | Q607C7 | 1 | 1 |  | 2E9S | Q9NRW1 | 1 | 1 |
| 3F0G | Q3JRA0 | 1 | 1 |  | 2EHZ | P0A108 | 1 | 1 |
| 1UVN | P11124 | 1 | 0 |  | 2E0L | P07998 | 1 | 0 |
| 5XVD | A0A3B6UEQ1 | 1 | 0 |  | 2ETJ | Q9X017 | 1 | 1 |
| 6IG2 | O74339 | 1 | 0 |  | 2D0B | Q6L6Q4 | 1 | 1 |
| 6SBI | Q8R0F8 | 1 | 1 |  | 2CMW | Q9HCP0 | 1 | 0 |
| 5W16 | A0A1J1ER74 | 1 | 0 |  | 2B4V | Q86MV5 | 1 | 0 |
| 3EG5 | P60766 | 1 | 1 |  | 2BCO | Q87Q40 | 1 | 1 |
| 7A8R | A0JN36 | 1 | 0 |  | 2BJI | P20456 | 1 | 1 |
| 3PWG | P0AES2 | 1 | 1 |  | 2BMX | P9WQB7 | 1 | 0 |
| 2FGY | O85042 | 1 | 1 |  | 2BNM | Q56185 | 1 | 1 |
| 5J92 | Q13Q05 | 1 | 1 |  | 2BZ1 | P0A7I7 | 1 | 1 |
| 8E9E | Q04631 | 1 | 0 |  | 2CWL | Q5SM21 | 1 | 0 |
| 2D40 | Q8X655 | 1 | 1 |  | 2C07 | Q8I2S7 | 1 | 0 |
| 4NBA | Q84II6 | 1 | 1 |  | 2C1I | Q8DP63 | 1 | 1 |
| 4IOU | Q8IUX4 | 1 | 1 |  | 2CC0 | Q54413 | 1 | 1 |
| 1WZO | Q5SJQ0 | 1 | 0 |  | 2CD9 | O93715 | 1 | 1 |
| 3C4E | P11309 | 1 | 0 |  | 2CF5 | O49482 | 1 | 1 |
| 3WCL | P37268 | 1 | 0 |  | 2CGL | P32171 | 1 | 0 |
| 2PGW | Q92YR6 | 1 | 0 |  | 2CHR | P05404 | 1 | 1 |
| 2OZ3 | C1DMY1 | 1 | 0 |  | 2C1C | Q3T905 | 1 | 1 |
| 7XJB | P22734 | 1 | 1 |  | 2F7V | Q8P8J5 | 1 | 0 |
| 2GLF | Q9WYJ9 | 1 | 1 |  | 2F8H | A0A0H2X6W0 | 1 | 0 |
| 4TXY | Q9KVG7 | 1 | 1 |  | 2F96 | Q9HY82 | 1 | 1 |
| 3B5Q | Q8A7C8 | 1 | 0 |  | 2HXV | Q9X2E8 | 1 | 1 |
| 2OOV | P12807 | 1 | 1 |  | 2HZH | Q8TG94 | 1 | 1 |
| 3PL1 | I6XD65 | 1 | 1 |  | 2I5E | Q8PU52 | 1 | 0 |
| 6PQH | X5KA67 | 1 | 0 |  | 2I5I | P59745 | 1 | 0 |
| 7Q2A | Q0S9X1 | 1 | 1 |  | 2I87 | Q5HEB7 | 1 | 0 |
| 2QYV | Q0I1B9 | 1 | 1 |  | 2ICS | Q837K0 | 1 | 1 |
| 4LHW | P61006 | 1 | 1 |  | 2HVW | Q8DSE5 | 1 | 1 |
| 1Z03 | O05935 | 1 | 1 |  | 2IGI | P0A784 | 1 | 1 |
| 2W2I | B2RXH2 | 1 | 1 |  | 2ISW | A8B2U2 | 1 | 1 |
| 5YY7 | P93836 | 1 | 1 |  | 2IUW | Q96Q83 | 1 | 1 |
| 4TQT | A0A0H3G9X2 | 1 | 1 |  | 2IVN | Q9UXT7 | 1 | 0 |
| 1GE5 | P81054 | 1 | 1 |  | 2IW0 | Q6DWK3 | 1 | 1 |
| 3OVB | O28126 | 1 | 0 |  | 2IXD | Q81FP2 | 1 | 1 |
| 1AQW | P09211 | 1 | 0 |  | 2IZR | Q9Y6M4 | 1 | 1 |
| 8CO3 | O43570 | 1 | 1 |  | 2IMR | Q9RW45 | 1 | 1 |
| 3HPY | Q7WYF5 | 1 | 1 |  | 2HHV | Q5KWC1 | 1 | 0 |
| 5G5T | Q58717 | 1 | 0 |  | 2HHS | Q45458 | 1 | 0 |
| 5F13 | Q04371 | 1 | 1 |  | 2HHP | P29468 | 1 | 0 |
| 8PP5 | P02794 | 1 | 1 |  | 2FFI | Q88M75 | 1 | 0 |
| 1OAJ | P00446 | 1 | 1 |  | 2FR5 | P56389 | 1 | 1 |
| 5YFD | B0S4Q0 | 1 | 1 |  | 2FTW | Q55DL0 | 1 | 1 |
| 7CBB | Q2G1N1 | 1 | 0 |  | 2G7N | Q45894 | 1 | 1 |
| 2PJ8 | P09955 | 1 | 1 |  | 2G8L | O59272 | 1 | 0 |
| 4P1C | Q00456 | 1 | 1 |  | 2GBZ | Q8P8S1 | 1 | 1 |
| 3M0Y | Q75WH8 | 1 | 1 |  | 2GJ8 | P25522 | 1 | 1 |
| 4XIW | Q39588 | 1 | 1 |  | 2GRU | Q9S5E2 | 1 | 1 |
| 6B67 | P35813 | 1 | 1 |  | 2GSR | P80031 | 1 | 0 |
| 3EWS | Q9UMR2 | 1 | 0 |  | 2GU1 | Q9KUL5 | 1 | 1 |
| 6O7D | B6YWB8 | 1 | 1 |  | 2GUI | P03007 | 1 | 1 |
| 1Z9X | Q9UIK4 | 1 | 0 |  | 2H6E | Q97YM2 | 1 | 1 |
| 4ZEL | P09172 | 1 | 1 |  | 2HAI | Q99AU2 | 1 | 0 |
| 1F1X | Q45135 | 1 | 1 |  | 2HCN | P14335 | 1 | 1 |
| 3RZU | O95630 | 1 | 1 |  | 2HGS | P48637 | 1 | 1 |
| 7C8Z | O52379 | 1 | 1 |  | 2AZQ | Q51433 | 1 | 1 |
| 5JF3 | Q8E378 | 1 | 1 |  | 1TOH | P04177 | 1 | 1 |
| 5J41 | P09211 | 1 | 0 |  | 2AU7 | P0A7A9 | 1 | 1 |
| 5IR4 | Q9I4G8 | 1 | 1 |  | 2AR3 | A0A6H3AMF3 | 1 | 1 |
| 4GN8 | Q64374 | 1 | 1 |  | 1W2Y | Q0P8G4 | 1 | 1 |
| 4GV1 | P31749 | 1 | 0 |  | 1W55 | Q9PM68 | 1 | 0 |
| 4GVE | P18140 | 1 | 1 |  | 1W6K | P48449 | 1 | 0 |
| 4GWN | Q16820 | 1 | 1 |  | 1W7C | Q96X16 | 1 | 1 |
| 4HM8 | P0A111 | 1 | 1 |  | 1W9Y | Q08506 | 1 | 0 |
| 4HUZ | Q9ZBB0 | 1 | 1 |  | 1WKQ | O34598 | 1 | 1 |
| 4HY4 | Q13490 | 1 | 1 |  | 1VQ2 | P16006 | 1 | 1 |
| 4I2A | P09838 | 1 | 0 |  | 1WNI | P20165 | 1 | 1 |
| 4IE5 | Q9C0B1 | 1 | 1 |  | 1WQL | Q51743 | 1 | 1 |
| 4IG8 | P00973 | 1 | 1 |  | 1WRA | Q8DQ62 | 1 | 1 |
| 4IGQ | Q53WJ1 | 1 | 1 |  | 1WW9 | Q84II6 | 1 | 1 |
| 4IHM | P29068 | 1 | 1 |  | 1WWR | O67050 | 1 | 1 |
| 4IIK | Q5ZSQ2 | 1 | 0 |  | 1X6M | Q51669 | 1 | 1 |
| 4IIT | A6T8I0 | 1 | 0 |  | 1XAF | A0A384KG77 | 1 | 1 |
| 4IKA | I3UIB4 | 1 | 1 |  | 1WPN | P37487 | 1 | 1 |
| 4GJZ | Q8N371 | 1 | 1 |  | 1XAH | Q6GGU4 | 1 | 1 |
| 4J0N | A0NLY7 | 1 | 1 |  | 1VK6 | P32664 | 1 | 0 |
| 4G9B | P77366 | 1 | 1 |  | 1VD6 | Q53W25 | 1 | 0 |
| 4G2D | C4KKZ9 | 1 | 1 |  | 1TT4 | Q8ZR41 | 1 | 1 |
| 4DXK | Q7D1T6 | 1 | 0 |  | 1TXO | P9WHW5 | 1 | 1 |
| 4DYO | Q9ULA0 | 1 | 1 |  | 1TZP | P0C0T5 | 1 | 0 |
| 4EFZ | Q3JRV4 | 1 | 1 |  | 1TZZ | Q89FH0 | 1 | 1 |
| 4EKD | P21279 | 1 | 1 |  | 1U0B | P21888 | 1 | 1 |
| 4EKN | Q58976 | 1 | 0 |  | 1U4B | P52026 | 1 | 0 |
| 4EUK | Q3S4A7 | 1 | 1 |  | 1VHE | P94521 | 1 | 0 |
| 4EWL | P9WJN3 | 1 | 1 |  | 1U8B | P06134 | 1 | 1 |
| 4F3W | B2HDU6 | 1 | 1 |  | 1ULZ | O67483 | 1 | 0 |
| 4F4R | Q1QT89 | 1 | 0 |  | 1UWY | P14384 | 1 | 1 |
| 4F50 | Q4JB80 | 1 | 0 |  | 1UX0 | P19079 | 1 | 1 |
| 4F6H | Q79MP6 | 1 | 1 |  | 1V10 | Q6H9H7 | 1 | 1 |
| 4F9U | Q9VRQ9 | 1 | 1 |  | 1V3Y | P43522 | 1 | 0 |
| 4FGM | Q5QTY1 | 1 | 1 |  | 1V4A | P30870 | 1 | 0 |
| 4FK9 | G2NHM6 | 1 | 0 |  | 1UJN | P83703 | 1 | 0 |
| 4FU0 | Q6WRY5 | 1 | 0 |  | 1XCR | Q9H0W9 | 1 | 1 |
| 4G7A | B2V8E3 | 1 | 1 |  | 1XFI | Q949P3 | 1 | 1 |
| 4DXA | P61224 | 1 | 1 |  | 1XFJ | Q9AAV3 | 1 | 0 |
| 4J1O | A1B198 | 1 | 1 |  | 1ZA0 | P9WNZ5 | 1 | 1 |
| 4JD0 | G4FFF4 | 1 | 0 |  | 1ZLI | P15086 | 1 | 1 |
| 4LD2 | Q82Y41 | 1 | 1 |  | 1ZPS | O26347 | 1 | 1 |
| 4LFY | B4EEU0 | 1 | 1 |  | 1ZX5 | O30200 | 1 | 0 |
| 4LIT | P27431 | 1 | 1 |  | 2A1X | O14832 | 1 | 1 |
| 4LRS | D1A3K8 | 1 | 1 |  | 2A3L | O80452 | 1 | 1 |
| 4LUK | P83194 | 1 | 1 |  | 1Z9T | P33644 | 1 | 0 |
| 4LXL | O94953 | 1 | 1 |  | 2A5D | P62330 | 1 | 1 |
| 4M51 | A6Q234 | 1 | 1 |  | 2A8B | Q15256 | 1 | 0 |
| 4M9Q | A8INQ0 | 1 | 1 |  | 2A8N | A9CK16 | 1 | 1 |
| 4MHR | Q1GNW5 | 1 | 0 |  | 2A8T | Q6TEC1 | 1 | 1 |
| 4MQY | P0A725 | 1 | 1 |  | 2A9F | Q99ZS1 | 1 | 1 |
| 4MV4 | P43873 | 1 | 1 |  | 2AQP | P57005 | 1 | 1 |
| 4N81 | Q5NMB8 | 1 | 0 |  | 2AQS | Q59623 | 1 | 1 |
| 4NAO | G8GV69 | 1 | 1 |  | 2A8A | P30996 | 1 | 1 |
| 4NHX | Q8N543 | 1 | 1 |  | 1Z3A | P68398 | 1 | 1 |
| 4NMI | Q2TDY4 | 1 | 0 |  | 1Z1W | O93655 | 1 | 1 |
| 4KRD | P17157 | 1 | 0 |  | 1Z1E | Q9SLV5 | 1 | 0 |
| 4J4M | U3KRG1 | 1 | 1 |  | 1XHB | O08912 | 1 | 1 |
| 4KNK | Q2FZK7 | 1 | 1 |  | 1XI6 | Q8TZH9 | 1 | 0 |
| 4KLI | P06746 | 1 | 0 |  | 1XM5 | P0A898 | 1 | 1 |
| 4JHT | P05050 | 1 | 1 |  | 1XMB | P54970 | 1 | 0 |
| 4JHV | M1GME7 | 1 | 1 |  | 1XR5 | P03303 | 1 | 0 |
| 4JID | A0A6L8PDI9 | 1 | 0 |  | 1Y0Y | O59196 | 1 | 1 |
| 4JND | P49594 | 1 | 1 |  | 1Y97 | Q9BQ50 | 1 | 0 |
| 4JO0 | F2RB80 | 1 | 1 |  | 1YAI | P00446 | 1 | 1 |
| 4JS0 | P60953 | 1 | 1 |  | 1YDY | P09394 | 1 | 1 |
| 4JXE | Q9P371 | 1 | 1 |  | 1YLK | P9WPJ7 | 1 | 1 |
| 4JZU | O35013 | 1 | 0 |  | 1YSJ | P54955 | 1 | 1 |
| 4K3Z | Q8YCV0 | 1 | 0 |  | 1YT3 | P09155 | 1 | 1 |
| 4K7E | Q6IVU2 | 1 | 0 |  | 1YVF | O93077 | 1 | 0 |
| 4K8G | A4XF23 | 1 | 1 |  | 1YVW | Q81G00 | 1 | 0 |
| 4K90 | P46075 | 1 | 1 |  | 1YW4 | Q7NU26 | 1 | 1 |
| 4KA7 | Q94AM1 | 1 | 1 |  | 2ATF | P60334 | 1 | 1 |
| 4KAV | Q7DD94 | 1 | 1 |  | 3SNG | Q0KFV0 | 1 | 1 |
| 4KB1 | P30014 | 1 | 1 |  | 3FW3 | P22748 | 1 | 1 |
| 4KM3 | B2IQ22 | 1 | 0 |  | 7CXZ | Q8LGJ5 | 1 | 1 |
| 4NNB | C5AJX5 | 1 | 1 |  | 6FBC | P19821 | 1 | 1 |
| 4DV8 | P15917 | 1 | 1 |  | 6F6M | Q5KW80 | 1 | 1 |
| 4DO7 | A0A0H3KNC4 | 1 | 1 |  | 6F47 | A0A384E149 | 1 | 1 |
| 8F5D | A0A0T7CQ89 | 1 | 0 |  | 7AGM | A0R5R2 | 1 | 1 |
| 8F8O | A3M8H2 | 1 | 1 |  | 6EDH | P37610 | 1 | 1 |
| 8F9Y | Q42546 | 1 | 1 |  | 6E0N | A0A4V8GZR7 | 1 | 0 |
| 8FIK | P31941 | 1 | 1 |  | 6E0M | A0A4V8GZR7 | 1 | 0 |
| 8G6P | G7CMG5 | 1 | 1 |  | 6E0K | G2SLH8 | 1 | 0 |
| 8GHB | P18054 | 1 | 1 |  | 7BGM | A0A072U2X9 | 1 | 1 |
| 8GOB | P0A9S5 | 1 | 1 |  | 6DZD | A5A677 | 1 | 0 |
| 8GXO | A0A7J7GXF2 | 1 | 1 |  | 7AFU | Q96SD1 | 1 | 0 |
| 8GY1 | A0A8F4Y4C2 | 1 | 0 |  | 6DXE | P13114 | 1 | 0 |
| 8H4P | I1S104 | 1 | 1 |  | 7BJ9 | Q9RMI1 | 1 | 1 |
| 8HCE | Q91XB0 | 1 | 0 |  | 7BLY | A2QZC8 | 1 | 1 |
| 8HE2 | A0A2S4W2W0 | 1 | 1 |  | 6DX9 | Q9MBB1 | 1 | 0 |
| 8HFN | A0QZY0 | 1 | 1 |  | 7BM4 | A0A3Q0KSG2 | 1 | 1 |
| 8I82 | D2B3F1 | 1 | 0 |  | 6DSW | E1C9K5 | 1 | 0 |
| 8IJ9 | P61294 | 1 | 1 |  | 7BU2 | P27250 | 1 | 1 |
| 8EJM | O43143 | 1 | 1 |  | 7C8K | E2DHI3 | 1 | 1 |
| 8JD8 | Q71S31 | 1 | 1 |  | 7CJ7 | A0A172U6X0 | 1 | 1 |
| 8E5D | P0DUH5 | 1 | 1 |  | 7CKF | Q96PP8 | 1 | 1 |
| 8CWP | Q06282 | 1 | 0 |  | 6DA7 | C6ZCR8 | 1 | 0 |
| 8ALK | Q5ZXN5 | 1 | 1 |  | 6DXA | P30079 | 1 | 0 |
| 8AW3 | Q57W17 | 1 | 1 |  | 7CLK | P9WNL1 | 1 | 1 |
| 8B2Q | D0EM77 | 1 | 1 |  | 7A7E | Q9I4L5 | 1 | 0 |
| 8B31 | A0A2W4LV58 | 1 | 0 |  | 7A19 | P80402 | 1 | 1 |
| 8B3Y | A0A286RCT9 | 1 | 0 |  | 6H40 | K5B7F3 | 1 | 1 |
| 8C0J | A0A482PQR2 | 1 | 1 |  | 6Y9C | D0C9N6 | 1 | 1 |
| 8C2O | P36548 | 1 | 1 |  | 6YA1 | P21347 | 1 | 1 |
| 8C2Z | Q9Y463 | 1 | 0 |  | 6H10 | Q6B0I6 | 1 | 1 |
| 8C46 | A0A8F7I7M8 | 1 | 1 |  | 6YL7 | A0A069AXA0 | 1 | 1 |
| 8C5D | P19157 | 1 | 0 |  | 6GU2 | P06493 | 1 | 0 |
| 8CD8 | Q8TL28 | 1 | 1 |  | 6GQD | P07902 | 1 | 1 |
| 8CDF | P76621 | 1 | 1 |  | 6G8U | P0DPK1 | 1 | 1 |
| 8CI9 | A0A0N9HQ36 | 1 | 1 |  | 6G5X | O75164 | 1 | 1 |
| 8CJK | P17752 | 1 | 1 |  | 6FYV | Q9HAZ1 | 1 | 0 |
| 8CVB | Q9XJ43 | 1 | 1 |  | 7A1Q | Q9NWT6 | 1 | 1 |
| 8DQB | A0A0H3FX83 | 1 | 1 |  | 6FYR | P49761 | 1 | 0 |
| 4DR0 | P50621 | 1 | 1 |  | 6YXH | Q9NUN7 | 1 | 1 |
| 8JUG | P09237 | 1 | 1 |  | 6FUL | O15550 | 1 | 1 |
| 8P0S | Q13464 | 1 | 0 |  | 6ZD1 | C5MCQ7 | 1 | 0 |
| 4C12 | Q2FZP6 | 1 | 1 |  | 6FT8 | P49759 | 1 | 0 |
| 4C24 | A0A0H2US48 | 1 | 1 |  | 6FPC | K4ZRC1 | 1 | 1 |
| 4C5W | O75936 | 1 | 1 |  | 6FOH | Q6P587 | 1 | 1 |
| 4C81 | P62368 | 1 | 1 |  | 6ZJE | Q9UIJ7 | 1 | 0 |
| 4C8E | B4EC22 | 1 | 1 |  | 6FJW | A0A0A7HF73 | 1 | 0 |
| 4C9B | P38919 | 1 | 0 |  | 6ZJF | O94768 | 1 | 0 |
| 4CE5 | Q0C8G1 | 1 | 0 |  | 6ZRN | P11234 | 1 | 1 |
| 4CHL | O95571 | 1 | 1 |  | 6FYL | P49760 | 1 | 0 |
| 4CNX | P00921 | 1 | 1 |  | 6Y2N | A4F980 | 1 | 1 |
| 4COQ | E8T502 | 1 | 1 |  | 6D5A | P9WKV3 | 1 | 0 |
| 4CXP | Q9C9G4 | 1 | 1 |  | 7CPH | P21335 | 1 | 1 |
| 4D0Y | A0A0H2ZP28 | 1 | 1 |  | 6AX7 | Q5UQC3 | 1 | 1 |
| 4DBF | Q8NQY2 | 1 | 1 |  | 7EKD | Q9FU53 | 1 | 0 |
| 4DEZ | A0QR77 | 1 | 0 |  | 6AW6 | A0A452CSQ2 | 1 | 0 |
| 4DL9 | D2Y3F4 | 1 | 1 |  | 6AGS | P26616 | 1 | 0 |
| 4BUP | Q3U8K7 | 1 | 0 |  | 7ETK | Q4WAW9 | 1 | 1 |
| 8OW8 | A0A242DI27 | 1 | 1 |  | 6AE9 | Q942P9 | 1 | 1 |
| 4BT2 | P23616 | 1 | 1 |  | 6ACI | B7UI21 | 1 | 1 |
| 4BJ4 | Q9HT86 | 1 | 0 |  | 7EUU | A0A2G5I8W0 | 1 | 1 |
| 8PNK | P06634 | 1 | 0 |  | 5ZW4 | O32036 | 1 | 0 |
| 8Q3U | P43166 | 1 | 1 |  | 5ZTP | A0A384E115 | 1 | 0 |
| 8Q57 | A0A0T9TPS2 | 1 | 1 |  | 6AYU | P9WN21 | 1 | 1 |
| 8SSF | D3DIV8 | 1 | 0 |  | 7FBG | Q812W6 | 1 | 1 |
| 8SU3 | Q9K499 | 1 | 1 |  | 5ZSX | A0A4V8GZK8 | 1 | 1 |
| 8TXY | Q7KZI7 | 1 | 0 |  | 7KSF | A0A1Q1N9V8 | 1 | 0 |
| 4AIG | P34179 | 1 | 1 |  | 7KVY | J3KJC6 | 1 | 0 |
| 4ARZ | Q00582 | 1 | 1 |  | 5ZNT | H9J9M0 | 1 | 1 |
| 4AU8 | Q00535 | 1 | 0 |  | 7LC5 | Q9WZJ0 | 1 | 1 |
| 4AXV | A7N805 | 1 | 1 |  | 5ZHF | Q47746 | 1 | 1 |
| 4AY7 | Q8PXZ6 | 1 | 1 |  | 5Z9X | A3KPE8 | 1 | 1 |
| 4B3F | P38935 | 1 | 0 |  | 5Z4A | Q9VI58 | 1 | 0 |
| 4B52 | E3E6L0 | 1 | 1 |  | 7LGP | P0AED8 | 1 | 0 |
| 4BFM | Q61846 | 1 | 0 |  | 5Z2M | P51150 | 1 | 1 |
| 4BIN | P63883 | 1 | 1 |  | 7FC3 | F6V9L3 | 1 | 0 |
| 4BMO | Q81G55 | 1 | 1 |  | 6D3J | Q8KSC8 | 1 | 1 |
| 7L3O | Q5CYN0 | 1 | 1 |  | 7E8J | A0A218P4J1 | 1 | 0 |
| 8THM | B5ILN4 | 1 | 1 |  | 6B8F | P02794 | 1 | 1 |
| 4O6I | P09992 | 1 | 1 |  | 6D0G | V5VHS3 | 1 | 1 |
| 5CA8 | Q9C0L9 | 1 | 0 |  | 6CWO | A5FCJ5 | 1 | 1 |
| 5CEE | Q2NK75 | 1 | 1 |  | 6CQS | E1R245 | 1 | 1 |
| 5CG9 | D2W6T1 | 1 | 1 |  | 7CVW | G8T6H8 | 1 | 1 |
| 5CGZ | Q88JX8 | 1 | 1 |  | 6CF3 | P32021 | 1 | 1 |
| 5CI3 | P69925 | 1 | 0 |  | 6C9C | Q02H34 | 1 | 1 |
| 5CJF | Q9ULX7 | 1 | 1 |  | 7D17 | A0A267GXB9 | 1 | 0 |
| 5CQI | Q9UH17 | 1 | 1 |  | 6C56 | O95749 | 1 | 0 |
| 5CUO | Q21A54 | 1 | 1 |  | 6C49 | A0A1E3MC83 | 1 | 1 |
| 5CUX | Q4JH30 | 1 | 0 |  | 6C0D | B2JR29 | 1 | 1 |
| 5CW6 | A0A8M3B525 | 1 | 1 |  | 6B00 | P00918 | 1 | 1 |
| 5D04 | Q9K169 | 1 | 1 |  | 7D7O | Q818A3 | 1 | 0 |
| 5D7W | P23694 | 1 | 1 |  | 6BSU | Q9LZJ3 | 1 | 1 |
| 5DEU | Q6N021 | 1 | 1 |  | 7DA2 | E1BSW7 | 1 | 0 |
| 5DK5 | Q10905 | 1 | 1 |  | 6BRK | Q60710 | 1 | 0 |
| 5DKT | Q8ILY1 | 1 | 0 |  | 6BPU | Q16878 | 1 | 1 |
| 5C7Q | Q6MIH8 | 1 | 0 |  | 7DC9 | Q94BU8 | 1 | 1 |
| 5DMM | Q47690 | 1 | 1 |  | 6BNZ | B6TPH0 | 1 | 1 |
| 5C5V | Q384W5 | 1 | 1 |  | 7DE2 | A0A2I1BSX0 | 1 | 1 |
| 5BQP | Q97W22 | 1 | 0 |  | 6BMC | G3XCJ9 | 1 | 1 |
| 4ZFQ | P9WKV2 | 1 | 0 |  | 7DLB | Q70MM3 | 1 | 1 |
| 4ZN6 | B7H1U5 | 1 | 0 |  | 6BGY | P29375 | 1 | 1 |
| 4ZRL | O17087 | 1 | 0 |  | 6BWG | P9WP83 | 1 | 0 |
| 4ZTY | Q8X1E4 | 1 | 1 |  | 6XOJ | A0A3B6UEU3 | 1 | 1 |
| 4ZUR | Q48935 | 1 | 1 |  | 6H56 | Q9I6M7 | 1 | 1 |
| 4ZVF | P0AA89 | 1 | 1 |  | 6XLR | P0CS93 | 1 | 1 |
| 5A5L | Q8DJE9 | 1 | 1 |  | 6SBP | Q9LXT4 | 1 | 1 |
| 5A61 | P30871 | 1 | 1 |  | 6SNQ | P36871 | 1 | 1 |
| 5A67 | Q9SIY3 | 1 | 0 |  | 6LSG | E5RPG3 | 1 | 1 |
| 5A7E | Q1W6B1 | 1 | 1 |  | 6SQ2 | P61006 | 1 | 1 |
| 5AJO | Q10471 | 1 | 0 |  | 6LPE | B3TFG2 | 1 | 1 |
| 5B5O | P45452 | 1 | 1 |  | 6SQW | Q9QY93 | 1 | 1 |
| 5B7Y | Q9WYP7 | 1 | 1 |  | 6LJH | A0A2U1Q018 | 1 | 1 |
| 5B8F | P57708 | 1 | 1 |  | 6STX | E3Q9X3 | 1 | 0 |
| 5BK9 | P83310 | 1 | 1 |  | 6LBK | Q5U315 | 1 | 0 |
| 5BXX | Q1GNW6 | 1 | 0 |  | 6LBD | T2B7E1 | 1 | 1 |
| 4Z8I | A0A0R4I979 | 1 | 1 |  | 6SAR | P66948 | 1 | 1 |
| 5DZ2 | Q9X839 | 1 | 1 |  | 6L5O | Q9NR30 | 1 | 1 |
| 5E3X | A0A140UHP3 | 1 | 1 |  | 6L46 | A0A1W6VP04 | 1 | 1 |
| 5GJ9 | Q41931 | 1 | 1 |  | 6T0Y | P84138 | 1 | 1 |
| 5GJB | A0A0U4DG08 | 1 | 0 |  | 6T6L | P19239 | 1 | 1 |
| 5GJU | P0A9P6 | 1 | 0 |  | 6T8M | Q86KR9 | 1 | 1 |
| 5H62 | Q8ZNP4 | 1 | 1 |  | 6L08 | O65896 | 1 | 0 |
| 5HPJ | Q6LM17 | 1 | 1 |  | 6KUN | Q01IX6 | 1 | 0 |
| 5HU3 | Q00168 | 1 | 1 |  | 6U2A | Q9KN86 | 1 | 1 |
| 5HVN | Q5NFS1 | 1 | 0 |  | 6KSF | P0CB42 | 1 | 0 |
| 5HX4 | Q8IUX4 | 1 | 0 |  | 6KPL | G3JPF7 | 1 | 0 |
| 5HXD | P0ACV7 | 1 | 1 |  | 6ULL | P42981 | 1 | 1 |
| 5HZX | Q7ZWC3 | 1 | 0 |  | 6SUK | P08473 | 1 | 1 |
| 5I0N | D9IEF7 | 1 | 0 |  | 6UN8 | P05057 | 1 | 1 |
| 5I1F | A4JT02 | 1 | 1 |  | 6LSV | Q9FFF6 | 1 | 1 |
| 5I8T | Q99JT9 | 1 | 1 |  | 6MDW | Q9H040 | 1 | 1 |
| 5IG1 | F2UPG5 | 1 | 0 |  | 6QRZ | Q4J6V7 | 1 | 1 |
| 5IKK | P56523 | 1 | 1 |  | 6R4Z | Q183R7 | 1 | 1 |
| 5GGB | A0QUZ2 | 1 | 0 |  | 6QO9 | Q81TB4 | 1 | 1 |
| 5E33 | Q9NY33 | 1 | 1 |  | 6P80 | D7Y2H2 | 1 | 0 |
| 5G5S | Q58717 | 1 | 0 |  | 6P5S | Q9H2X6 | 1 | 0 |
| 5G2U | Q8A7C8 | 1 | 0 |  | 6RA7 | Q9UKE5 | 1 | 0 |
| 5E68 | Q8Z4D7 | 1 | 1 |  | 6RB4 | P49642 | 1 | 0 |
| 5EEI | F8W4B7 | 1 | 1 |  | 6RCX | B1MD73 | 1 | 1 |
| 5EHF | A0A0M3U1T9 | 1 | 1 |  | 6RI6 | I1VE66 | 1 | 1 |
| 5EKS | V5V8R5 | 1 | 1 |  | 6RQQ | Q16790 | 1 | 1 |
| 5EMI | B2J2S4 | 1 | 1 |  | 6M37 | P17904 | 1 | 0 |
| 5ERM | A2PZA5 | 1 | 1 |  | 6OH6 | A0A158RFK9 | 1 | 1 |
| 5ES1 | Q96L34 | 1 | 0 |  | 6NPC | A0A4V8H042 | 1 | 1 |
| 5ESR | Q9A919 | 1 | 0 |  | 6NOZ | W8QLX4 | 1 | 0 |
| 5F8H | E5RPG2 | 1 | 1 |  | 6NIB | G7JT50 | 1 | 0 |
| 5FJ6 | P11124 | 1 | 0 |  | 6NDS | A0A0D5YK08 | 1 | 1 |
| 5FJK | Q9H3R0 | 1 | 1 |  | 6NBO | A0A0H3KPJ4 | 1 | 1 |
| 5FYZ | Q9UGL1 | 1 | 1 |  | 6S0P | Q9SJI9 | 1 | 1 |
| 5FZO | Q15652 | 1 | 1 |  | 6N6J | Q9Y3B8 | 1 | 0 |
| 5G0X | Q9HXM1 | 1 | 1 |  | 6S2T | Q5SHL3 | 1 | 1 |
| 5G1A | Q70I53 | 1 | 1 |  | 6N0K | O00625 | 1 | 1 |
| 5G3C | I7AL37 | 1 | 1 |  | 6MRF | V5VCW7 | 1 | 0 |
| 4NRE | O15296 | 1 | 1 |  | 6O3P | Q9DCN1 | 1 | 1 |
| 4Z84 | P00517 | 1 | 0 |  | 6USO | Q0QHL8 | 1 | 1 |
| 4Z5S | Q55688 | 1 | 0 |  | 6V0T | P35816 | 1 | 1 |
| 4Q0Q | Q93UQ5 | 1 | 1 |  | 6VDC | A0QYZ2 | 1 | 0 |
| 4Q1L | P84907 | 1 | 1 |  | 6IQZ | Q12737 | 1 | 1 |
| 4Q3X | P30967 | 1 | 1 |  | 6IP0 | F4KIX0 | 1 | 1 |
| 4Q6X | A0A0D4WV12 | 1 | 1 |  | 6XCF | P0DPI0 | 1 | 1 |
| 4QGN | Q9BV57 | 1 | 1 |  | 6INC | W9BHF3 | 1 | 1 |
| 4QGS | Q46856 | 1 | 1 |  | 6II7 | Q8IJA9 | 1 | 1 |
| 4QJL | A0PQD8 | 1 | 1 |  | 6IHR | Q9RL81 | 1 | 1 |
| 4QKD | Q9BT30 | 1 | 1 |  | 6IH0 | O67648 | 1 | 1 |
| 4QLZ | Q5DE13 | 1 | 1 |  | 6XHK | Q2G1B9 | 1 | 0 |
| 4QP5 | P10547 | 1 | 1 |  | 6IG4 | O74339 | 1 | 0 |
| 4QU2 | P0C872 | 1 | 1 |  | 6XI7 | P01116-2 | 1 | 1 |
| 4QUP | Q92ZP9 | 1 | 1 |  | 6XBU | O75417 | 1 | 0 |
| 4R8Z | Q9HV27 | 1 | 1 |  | 6IE3 | Q13686 | 1 | 1 |
| 4RAG | P35813 | 1 | 1 |  | 6IB8 | P0ADG4 | 1 | 0 |
| 4RD4 | Q980A5 | 1 | 1 |  | 6I03 | P71447 | 1 | 1 |
| 4PUT | Q949P2 | 1 | 1 |  | 6HPH | Q9H611 | 1 | 1 |
| 4RG8 | A0A0A0R5X6 | 1 | 1 |  | 6HHM | T2KPK5 | 1 | 1 |
| 4PP4 | P03134 | 1 | 0 |  | 6HFQ | P27708 | 1 | 1 |
| 4PF4 | P53355 | 1 | 0 |  | 6HEI | Q96RU2 | 1 | 0 |
| 4O7I | C4M4T9 | 1 | 0 |  | 6H8O | O39828 | 1 | 0 |
| 4O7X | Q6P6C2 | 1 | 1 |  | 6XJJ | B8M9K5 | 1 | 1 |
| 4OA8 | Q2GKC7 | 1 | 0 |  | 6H8N | O34798 | 1 | 1 |
| 4OH1 | B0NC68 | 1 | 1 |  | 6H5W | P12821 | 1 | 1 |
| 4OKE | P9WJ73 | 1 | 0 |  | 6ICL | H9N290 | 1 | 1 |
| 4ONW | Q9KQ52 | 1 | 0 |  | 6X6Z | P12689 | 1 | 1 |
| 4OV4 | B0SN40 | 1 | 1 |  | 6IYB | P51149 | 1 | 1 |
| 4OX3 | O34866 | 1 | 1 |  | 6X5X | P0DQT6 | 1 | 1 |
| 4P0P | Q96NY9 | 1 | 1 |  | 6VGO | Q9H4B8 | 1 | 0 |
| 4P10 | Q96IY4 | 1 | 1 |  | 6VL1 | A0A386KZ50 | 1 | 1 |
| 4P53 | H2K887 | 1 | 1 |  | 6JYV | Q9HWJ0 | 1 | 0 |
| 4P7X | Q989T9 | 1 | 1 |  | 6JY1 | Q60358 | 1 | 0 |
| 4P9G | Q9REI7 | 1 | 1 |  | 6JTZ | P46063 | 1 | 0 |
| 4PBE | A0A060GPP4 | 1 | 1 |  | 6VOP | Q46890 | 1 | 0 |
| 4PCP | A0A060GYS1 | 1 | 1 |  | 6VOQ | Q0QC76 | 1 | 1 |
| 4PFH | O50580 | 1 | 1 |  | 6JQW | P0CT50 | 1 | 1 |
| 4Z71 | P9WI55 | 1 | 1 |  | 6VPM | O14965 | 1 | 0 |
| 4RGV | Q58122 | 1 | 1 |  | 6VU9 | B2FN79 | 1 | 1 |
| 4RN7 | Q183J9 | 1 | 1 |  | 6VVC | Q5ZSB6 | 1 | 0 |
| 4X2P | P11444 | 1 | 1 |  | 6JQD | Q4WPJ0 | 1 | 1 |
| 4X7Y | Q49492 | 1 | 1 |  | 6VWQ | I0CBY7 | 1 | 1 |
| 4XAE | Q9LHN8 | 1 | 0 |  | 6W04 | C4LXK0 | 1 | 1 |
| 4XCH | B2CMA5 | 1 | 1 |  | 6W1G | Q88CC1 | 1 | 1 |
| 4XEP | P66881 | 1 | 1 |  | 6WBQ | F1QCV2 | 1 | 1 |
| 4XEZ | P21816 | 1 | 1 |  | 6WE5 | O84777 | 1 | 0 |
| 4XJ5 | Q9KVG7 | 1 | 1 |  | 6WKC | Q55012 | 1 | 0 |
| 4XJ6 | Q6XGD8 | 1 | 1 |  | 6J5X | Q8NLC0 | 1 | 1 |
| 4XXF | A0A0J9X279 | 1 | 1 |  | 6J4M | Q94IC4 | 1 | 1 |
| 4Y0X | P9WI73 | 1 | 1 |  | 6WNU | U5CJP3 | 1 | 0 |
| 4Y7E | F5HR99 | 1 | 0 |  | 6X5G | Q9UQM7 | 1 | 0 |
| 4YF4 | P9WPJ6 | 1 | 1 |  | 6J3D | E0U503 | 1 | 1 |
| 4YLM | Q9I1L4 | 1 | 0 |  | 5YKN | Q8GUI6 | 1 | 1 |
| 4YZG | P49599 | 1 | 1 |  | 5YHT | P95189 | 1 | 1 |
| 4Z1O | D0KMY9 | 1 | 0 |  | 6R33 | A2QHE5 | 1 | 1 |
| 4WQK | P0AE05 | 1 | 1 |  | 4A15 | Q9HM14 | 1 | 0 |
| 4RKE | O18333 | 1 | 1 |  | 7QP3 | A0A1B8GTG7 | 1 | 1 |
| 4WNO | O75385 | 1 | 0 |  | 3UKO | Q96533 | 1 | 1 |
| 4WK7 | O75173 | 1 | 1 |  | 3USS | Q9I0N5 | 1 | 1 |
| 4RPE | Q8RNT4 | 1 | 1 |  | 3TY2 | Q9KI21 | 1 | 0 |
| 4RT5 | D5SVJ2 | 1 | 1 |  | 7QOD | Q8NQV9 | 1 | 1 |
| 4RW5 | P0CE82 | 1 | 1 |  | 7WX0 | Q8WNN6 | 1 | 1 |
| 4TMX | G0S8G9 | 1 | 1 |  | 7X4Q | P0DSP3 | 1 | 1 |
| 4TND | P34947 | 1 | 1 |  | 3TX8 | Q59284 | 1 | 0 |
| 4TQR | Q97W02 | 1 | 1 |  | 7QFY | M1UZ70 | 1 | 1 |
| 4U4I | G5CS11 | 1 | 0 |  | 5WDR | F2UBE5 | 1 | 1 |
| 4UAS | P95649 | 1 | 1 |  | 7QBP | A4F7B2 | 1 | 1 |
| 4UAV | Q94K71 | 1 | 1 |  | 7Q73 | Q10295 | 1 | 0 |
| 4UD4 | O13833 | 1 | 0 |  | 7Q5V | Q9GZT9 | 1 | 1 |
| 4UEG | O15488 | 1 | 1 |  | 3TWZ | Q818Z9 | 1 | 1 |
| 4V06 | Q8IWU9 | 1 | 1 |  | 3UJK | O04719 | 1 | 1 |
| 4W9W | Q9NSY1 | 1 | 0 |  | 7Q0D | P00915 | 1 | 1 |
| 4WB8 | P17612 | 1 | 1 |  | 3WKQ | A4IL26 | 1 | 1 |
| 4WIG | P9WPH3 | 1 | 1 |  | 3WI9 | Q5L1X8 | 1 | 1 |
| 4WMA | Q3U4G3 | 1 | 1 |  | 7PLD | Q9A9Z1 | 1 | 1 |
| 8A5X | P00720 | 1 | 0 |  | 3TWO | D0ITF8 | 1 | 1 |
| 2R8K | Q04049 | 1 | 1 |  | 3TTE | A4YVM8 | 1 | 1 |
| 3FPI | Q8ZBP7 | 1 | 0 |  | 7X5Y | P93836 | 1 | 1 |
| 5SWW | P31941 | 1 | 1 |  | 7T7N | O94725 | 1 | 0 |
| 4EG2 | Q9KSM5 | 1 | 1 |  | 3TTC | A0A0H3JHT3 | 1 | 1 |
| 5EUF | I9VHL9 | 1 | 1 |  | 7XS4 | O64642 | 1 | 0 |
| 3VI1 | Q03023 | 1 | 1 |  | 7SEZ | P04311 | 1 | 0 |
| 2BOY | Q8G9L3 | 1 | 1 |  | 7XWY | O42861 | 1 | 0 |
| 5GJA | Q41931 | 1 | 1 |  | 7XZ4 | K3ZJM0 | 1 | 0 |
| 3R2J | A4HRG8 | 1 | 1 |  | 7PFH | Q0I9X8 | 1 | 1 |
| 5NA8 | Q8A6N1 | 1 | 1 |  | 7PCR | P56185 | 1 | 0 |
| 6G80 | K5B7F3 | 1 | 0 |  | 7PP7 | Q41510 | 1 | 1 |
| 3CE1 | A0ZPR9 | 1 | 1 |  | 5WPN | A0A075ML49 | 1 | 1 |
| 2HK0 | A9CH28 | 1 | 0 |  | 5WDQ | P01112 | 1 | 1 |
| 1T91 | P51149 | 1 | 1 |  | 3VN5 | O67644 | 1 | 0 |
| 1UZR | P9WH71 | 1 | 1 |  | 7VJV | Q3KRA9 | 1 | 1 |
| 7WSB | Q4WAW9 | 1 | 1 |  | 7VLZ | A0A8B3DGT3 | 1 | 1 |
| 3COS | P08319 | 1 | 1 |  | 7RSF | P23908 | 1 | 0 |
| 4FXY | P42676 | 1 | 1 |  | 3ZPG | D0CB74 | 1 | 1 |
| 4XH9 | Q7Z628 | 1 | 0 |  | 3U9W | P09960 | 1 | 1 |
| 4IL3 | M4GGS2 | 1 | 1 |  | 3ZGJ | Q9Z4X7 | 1 | 1 |
| 6VGR | Q9H4B8 | 1 | 0 |  | 3ZFK | Q47112 | 1 | 1 |
| 1WA5 | P62825 | 1 | 1 |  | 3ZT9 | Q2RIF7 | 1 | 1 |
| 2GCU | Q9C8L4 | 1 | 1 |  | 7W6Y | C7R5Z1 | 1 | 1 |
| 6DLI | Q5SHT7 | 1 | 0 |  | 3U7K | Q5HGZ3 | 1 | 1 |
| 5WRU | O77392 | 1 | 0 |  | 7REI | Q96SZ5 | 1 | 1 |
| 4NHY | Q8N543 | 1 | 1 |  | 7WGH | A0A364LX79 | 1 | 0 |
| 3NR9 | P49760 | 1 | 0 |  | 3UAR | Q60CN1 | 1 | 0 |
| 6MJP | O30650 | 1 | 0 |  | 7VI8 | A6T7U7 | 1 | 1 |
| 4FN6 | Q6GEY1 | 1 | 0 |  | 7SXF | P49840 | 1 | 0 |
| 4C5Z | G3XP38 | 1 | 0 |  | 7R97 | P0A7Y0 | 1 | 1 |
| 4DKI | A0A0H2WXF8 | 1 | 0 |  | 7SJ3 | P11802 | 1 | 0 |
| 4N0G | P49598 | 1 | 1 |  | 3TYP | Q82UI9 | 1 | 0 |
| 4W4U | N1P0J5 | 1 | 0 |  | 7V4Q | D7RF80 | 1 | 0 |
| 6U7B | D7Y2H2 | 1 | 1 |  | 3ZVS | O29917 | 1 | 1 |
| 4OKM | B5HDJ6 | 1 | 1 |  | 3ZDU | Q8IVW4 | 1 | 0 |
| 4CZ1 | Q81PP9 | 1 | 1 |  | 3X1Z | Q62636 | 1 | 1 |
| 6R58 | Q183R7 | 1 | 1 |  | 7WJL | Q02959 | 1 | 1 |
| 3G8Q | Q8TWU6 | 1 | 1 |  | 3ZX1 | A0A0H3PBA4 | 1 | 1 |
| 6IQD | P42327 | 1 | 1 |  | 3UH0 | P07236 | 1 | 1 |
| 5J72 | Q183L9 | 1 | 1 |  | 3UHM | P47205 | 1 | 1 |
| 1FUI | P69922 | 1 | 1 |  | 3WQS | O96693 | 1 | 1 |
| 7NWC | P54687 | 1 | 0 |  | 7QUE | Q9UEE5 | 1 | 0 |
| 5OLK | A3XHF9 | 1 | 1 |  | 4A03 | P9WNS1 | 1 | 1 |
| 1U0W | P30074 | 1 | 0 |  | 7QTB | P24021 | 1 | 1 |
| 4IL0 | Q46915 | 1 | 0 |  | 3U2U | P46976 | 1 | 1 |
| 2I5Q | P77215 | 1 | 0 |  | 7P5T | P9WPL5 | 1 | 0 |
| 4GU7 | Q9FBY9 | 1 | 0 |  | 3U9I | A5UXJ3 | 1 | 0 |
| 2PK0 | Q8VQA1 | 1 | 1 |  | 7LPZ | O43414 | 1 | 0 |
| 4IRQ | Q9UBV7 | 1 | 1 |  | 3VTG | P31580 | 1 | 1 |
| 4CJN | A0A0J9X1X5 | 1 | 0 |  | 7ZKX | P78362 | 1 | 0 |
| 4X8I | Q8TZW4 | 1 | 1 |  | 3VQJ | H1AAP2 | 1 | 1 |
| 3TZQ | B2HKN3 | 1 | 0 |  | 5XNE | Q04777 | 1 | 1 |
| 4WVZ | A0A140UH61 | 1 | 1 |  | 3V4K | Q9HC16 | 1 | 1 |
| 6NIC | G7JT50 | 1 | 0 |  | 5WRT | Q4VUZ3 | 1 | 1 |
| 1NVM | P51016 | 1 | 1 |  | 7TQO | Q9NSU2 | 1 | 0 |
| 2JLE | Q72547 | 1 | 0 |  | 7NUU | Q9Y303 | 1 | 1 |
| 3OVG | Q4A724 | 1 | 1 |  | 3THP | Q96BT7 | 1 | 1 |
| 3V4F | P20171 | 1 | 1 |  | 3TH1 | P11451 | 1 | 1 |
| 5VE4 | B2TEQ2 | 1 | 1 |  | 7U1Y | Q9UT55 | 1 | 1 |
| 2E67 | Q53WD3 | 1 | 1 |  | 3TC3 | Q4J9T1 | 1 | 0 |
| 2GOK | Q8U8Z6 | 1 | 1 |  | 5XWI | A1YYW7 | 1 | 1 |
| 6HUF | P51159 | 1 | 1 |  | 7NWA | P54687 | 1 | 0 |
| 1LLU | Q9HTD9 | 1 | 1 |  | 7U56 | A0A0H3GSI5 | 1 | 1 |
| 3HIY | C7AJA4 | 1 | 0 |  | 7NA9 | P10844 | 1 | 1 |
| 6S2V | Q5SHL3 | 1 | 1 |  | 3VAT | Q2HJH1 | 1 | 1 |
| 3IAC | Q8ZM23 | 1 | 0 |  | 3VNX | H7CGH2 | 1 | 1 |
| 3JVI | C4LSE7 | 1 | 0 |  | 7M7N | Q9Y253 | 1 | 1 |
| 4XVZ | Q49492 | 1 | 1 |  | 3T34 | P42697 | 1 | 1 |
| 6W1K | Q337M4 | 1 | 1 |  | 7M07 | Q9UGP5 | 1 | 1 |
| 7KWS | Q0P8H3 | 1 | 0 |  | 3VEN | Q70IY1 | 1 | 1 |
| 5OES | M1CSC4 | 1 | 1 |  | 5Y86 | O43781 | 1 | 0 |
| 5HNM | Q47746 | 1 | 1 |  | 3VJ8 | P37268 | 1 | 0 |
| 6PJV | Q15465 | 1 | 1 |  | 7UAE | Q16819 | 1 | 1 |
| 3QTP | P51555 | 1 | 1 |  | 5YBN | A0A1E1FFL0 | 1 | 1 |
| 7WL0 | Q6P6C2 | 1 | 1 |  | 7LT2 | D6WI29 | 1 | 0 |
| 3SFW | Q846U5 | 1 | 1 |  | 5YHO | P05361 | 1 | 1 |
| 7NF4 | A2QHE5 | 1 | 1 |  | 5XZG | Q8C6L5 | 1 | 0 |
| 7DIE | Q8EWB1 | 1 | 1 |  | 7NZ9 | Q6P6J0 | 1 | 1 |
| 3TBB | J9PBR2 | 1 | 1 |  | 3VOW | Q9NRW3 | 1 | 1 |
| 6LNH | E2GIN1 | 1 | 1 |  | 3WA2 | P46881 | 1 | 1 |
| 6UOK | P06746 | 1 | 0 |  | 5X0J | Q5JD03 | 1 | 0 |
| 5K52 | A0A193CDY9 | 1 | 0 |  | 7YH4 | Q8IYS1 | 1 | 1 |
| 4OAV | Q05823 | 1 | 1 |  | 7UOF | Q58885 | 1 | 1 |
| 3TO3 | Q81RQ8 | 1 | 0 |  | 7OLT | Q5AR53 | 1 | 1 |
| 4F9D | P75906 | 1 | 1 |  | 7OFL | A0A5M3MXY8 | 1 | 1 |
| 1NYQ | Q8NW68 | 1 | 1 |  | 7UOI | A0A1S8KJG1 | 1 | 0 |
| 7SKB | V5VGT0 | 1 | 0 |  | 3UYQ | P07451 | 1 | 1 |
| 3CIA | Q7WVY1 | 1 | 1 |  | 5WZ3 | A0A109PRQ3 | 1 | 1 |
| 3V15 | Q88RA3 | 1 | 0 |  | 3V1V | Q9F1Y6 | 1 | 1 |
| 2PNC | Q29437 | 1 | 1 |  | 7YLL | P58965 | 1 | 0 |
| 2OGJ | Q7CS13 | 1 | 1 |  | 3W6P | O00429 | 1 | 1 |
| 4COG | B4E9I9 | 1 | 1 |  | 7YVV | A0A7H8HIR4 | 1 | 1 |
| 7RHW | Q96X30 | 1 | 1 |  | 7O7I | Q9H422 | 1 | 0 |
| 3SSM | Q83WF2 | 1 | 1 |  | 7Y1U | A0A7M1LFL5 | 1 | 0 |
| 4IOB | Q9I4L5 | 1 | 0 |  | 3W42 | P17906 | 1 | 1 |
| 5C05 | A0A0M3Q1Q3 | 1 | 0 |  | 5WWD | Q9CA40 | 1 | 1 |
| 1HI9 | P26902 | 1 | 1 |  | 7ODY | A0A7U3TBV3 | 1 | 1 |
| 4KIK | O14920 | 1 | 0 |  | 3V4B | B3PDB1 | 1 | 1 |
| 6ZJB | Q9UIJ7 | 1 | 0 |  | 7TA5 | B7KEP8 | 1 | 0 |
| 5CUU | Q4JH30 | 1 | 0 |  | 7OPQ | P51159 | 1 | 1 |
| 1N8P | P31373 | 1 | 0 |  | 3TR6 | Q83D22 | 1 | 1 |
| 1SQI | P32755 | 1 | 1 |  | 1YQD | Q94G59 | 2 | 2 |
| 1Z5H | O93655 | 1 | 1 |  | 8HFB | P42106 | 2 | 2 |
| 3S2S | Q8DSG2 | 1 | 1 |  | 7V4H | A0A0R0EVM7 | 2 | 0 |
| 6J4J | Q94IC4 | 1 | 1 |  | 1YNB | O28840 | 2 | 0 |
| 2HWT | O39828 | 1 | 0 |  | 2AFW | Q16769 | 2 | 1 |
| 4HN8 | A4XRL3 | 1 | 0 |  | 2AEB | P05089 | 2 | 2 |
| 6B9S | A9A1T2 | 1 | 1 |  | 6ZS1 | G0S1F8 | 2 | 1 |
| 2H1J | Q5L1D2 | 1 | 1 |  | 8CR7 | P14756 | 2 | 2 |
| 2AE8 | P64373 | 1 | 1 |  | 7PJC | A0A1D8PSA9 | 2 | 0 |
| 5HMQ | Q88JU3 | 1 | 1 |  | 7U6O | A0A090M4C4 | 2 | 0 |
| 6DVV | Q9AGA6 | 1 | 1 |  | 1ZJC | A0A0H3K3S3 | 2 | 2 |
| 3DWC | Q6ZXC0 | 1 | 1 |  | 4NU1 | Q9WV60 | 2 | 1 |
| 6S21 | Q8A2F6 | 1 | 1 |  | 4NTK | P65870 | 2 | 2 |
| 6D0P | A0A0C0C2K2 | 1 | 1 |  | 7UY4 | P0AG05 | 2 | 2 |
| 1J1W | P16100 | 1 | 0 |  | 4MHP | B7QK46 | 2 | 0 |
| 6IM3 | C0QRB5 | 1 | 1 |  | 7UG9 | P77766 | 2 | 2 |
| 6TP5 | Q9NXG6 | 1 | 1 |  | 1G71 | Q9P9H1 | 2 | 0 |
| 6UMQ | Q9H993 | 1 | 1 |  | 4MCW | C0QQ26 | 2 | 2 |
| 5H63 | Q8ZNP4 | 1 | 1 |  | 6XFR | A0A0E3ZJD7 | 2 | 2 |
| 3O6X | Q5LGP1 | 1 | 1 |  | 1X8G | P26918 | 2 | 1 |
| 3DC8 | Q0PQZ5 | 1 | 1 |  | 7TDP | A0A0F0G8G2 | 2 | 2 |
| 1M0W | Q08220 | 1 | 1 |  | 4UOP | Q8Y989 | 2 | 2 |
| 2PMP | Q9CAK8 | 1 | 1 |  | 1ZKL | Q13946 | 2 | 2 |
| 5WVU | Q5SLM3 | 1 | 1 |  | 6U0Y | B2FTM1 | 2 | 2 |
| 5BPX | Q9REI7 | 1 | 1 |  | 7UU4 | M1GSK9 | 2 | 2 |
| 1Q74 | P9WJN3 | 1 | 1 |  | 1WS0 | Q819U0 | 2 | 2 |
| 5TPR | Q3M6C3 | 1 | 1 |  | 4OJV | P22434 | 2 | 2 |
| 1T75 | P61517 | 1 | 1 |  | 2OBA | Q9I0H2 | 2 | 2 |
| 1GKQ | Q7SIE9 | 1 | 1 |  | 4TWE | Q9UQQ1 | 2 | 2 |
| 2CLB | P95855 | 1 | 1 |  | 2DQ4 | Q5SKS4 | 2 | 2 |
| 4AC8 | P9WH69 | 1 | 1 |  | 3RR1 | B2UCA8 | 2 | 0 |
| 2XTZ | P18064 | 1 | 1 |  | 6S1Y | A0NFU8 | 2 | 1 |
| 6BBO | Q6NTF7 | 1 | 1 |  | 6RZR | Q7WYA8 | 2 | 2 |
| 2Y7J | P15735 | 1 | 0 |  | 5THW | A0A0H3KRF1 | 2 | 2 |
| 5YBL | Q5AR34 | 1 | 1 |  | 1CA1 | P0C216 | 2 | 2 |
| 1CF2 | P10618 | 1 | 0 |  | 7Z68 | E3ULB4 | 2 | 2 |
| 2PK9 | P17157 | 1 | 0 |  | 4QXD | C4M633 | 2 | 2 |
| 6PK4 | Q9NRF8 | 1 | 0 |  | 4R60 | Q8P839 | 2 | 2 |
| 5GIV | Q9RRR3 | 1 | 1 |  | 4ICQ | A0A0H2UN95 | 2 | 2 |
| 4I6V | D5SMT1 | 1 | 1 |  | 2EER | Q96XE0 | 2 | 0 |
| 2IVO | Q9UXT7 | 1 | 0 |  | 5CNX | P76524 | 2 | 2 |
| 1OZ9 | O67367 | 1 | 0 |  | 7Y9P | P22144 | 2 | 2 |
| 5DMX | B2I1J3 | 1 | 0 |  | 8AXY | Q6GGE2 | 2 | 2 |
| 5OTN | Q7ZWC3 | 1 | 1 |  | 6QRO | G8UMP8 | 2 | 1 |
| 7CVU | G8T6H8 | 1 | 0 |  | 2ET1 | P45850 | 2 | 2 |
| 6J66 | A0A0C5AQI9 | 1 | 1 |  | 4RC8 | Q54764 | 2 | 2 |
| 4C3O | Q8ZPH0 | 1 | 0 |  | 8ARV | Q9HW35 | 2 | 1 |
| 6DRH | A8GG79 | 1 | 0 |  | 1BF6 | P45548 | 2 | 2 |
| 6OJM | A0A077EJG6 | 1 | 0 |  | 1B66 | P27213 | 2 | 2 |
| 5FB3 | A3MTM6 | 1 | 1 |  | 6R78 | G8B4G1 | 2 | 2 |
| 4G9I | Q5JII4 | 1 | 1 |  | 4ROP | Q5N3P4 | 2 | 2 |
| 3DG7 | A0QTN8 | 1 | 1 |  | 7ZO4 | P52700 | 2 | 2 |
| 1WMW | Q5SMD0 | 1 | 0 |  | 8IQ1 | P00442 | 2 | 2 |
| 2AMJ | P0AEY7 | 1 | 0 |  | 4QKU | B4ECX4 | 2 | 0 |
| 1MPY | P06622 | 1 | 1 |  | 8ACR | Q9HZQ8 | 2 | 2 |
| 4AEE | A3DM60 | 1 | 0 |  | 8SQT | Q2YKI4 | 2 | 2 |
| 7DZ6 | A0A249Q1V1 | 1 | 1 |  | 4RQT | P06525 | 2 | 2 |
| 7MU5 | Q9H773 | 1 | 1 |  | 7ZN6 | G2QFD0 | 2 | 2 |
| 7Q74 | Q10295 | 1 | 0 |  | 3SY8 | Q9HX69 | 2 | 1 |
| 7QX8 | Q84WV0 | 1 | 0 |  | 4ZNG | A8WBX8 | 2 | 1 |
| 7UM1 | A0A172JIC8 | 1 | 0 |  | 1D3V | P07824 | 2 | 2 |
| 7SEK | Q8BP48 | 1 | 1 |  | 7VSD | P0A7Y4 | 2 | 2 |
| 7TQQ | Q9NSU2 | 1 | 0 |  | 7AV7 | A0A2K3D6R4 | 2 | 2 |
| 7UPI | O14807 | 1 | 1 |  | 7WNU | P9WGZ9 | 2 | 2 |
| 8DG4 | P69949 | 1 | 0 |  | 6V71 | C6XID6 | 2 | 2 |
| 7VRD | P30575 | 1 | 1 |  | 2B5W | Q977U7 | 2 | 2 |
| 7XJG | P23070 | 1 | 0 |  | 7WW2 | A0A4Q9D6T1 | 2 | 2 |
| 3JZE | P06204 | 1 | 1 |  | 7L9F | Q9NX46 | 2 | 2 |
| 7W5J | A0A5S9I252 | 1 | 1 |  | 4P4M | Q9U6N3 | 2 | 0 |
| 7A03 | A0A822ZZF9 | 1 | 0 |  | 7XHX | K4PWX3 | 2 | 2 |
| 6O64 | O48661 | 1 | 0 |  | 7XHW | K0ITE2 | 2 | 2 |
| 1OJ7 | Q46856 | 1 | 0 |  | 5ZLP | P94845 | 2 | 0 |
| 2W8S | Q45087 | 1 | 1 |  | 8EDJ | Q7YR23 | 2 | 2 |
| 1KWM | P15086 | 1 | 1 |  | 6U10 | B2FTM1 | 2 | 2 |
| 2OTD | A0A0H2V2B5 | 1 | 0 |  | 1DDZ | Q43060 | 2 | 2 |
| 2W4L | P32321 | 1 | 1 |  | 2C77 | P60339 | 2 | 2 |
| 1NFG | Q8VTT5 | 1 | 1 |  | 2C78 | Q5SHN6 | 2 | 2 |
| 7D0V | A0A2I4HXH5 | 1 | 1 |  | 6TVE | P21589 | 2 | 2 |
| 3HM7 | Q9KAH8 | 1 | 1 |  | 5BN4 | Q74MJ7 | 2 | 2 |
| 3MLE | O24872 | 1 | 1 |  | 7XJO | P08253 | 2 | 2 |
| 3M1R | P42068 | 1 | 1 |  | 4QGL | Q81MI9 | 2 | 2 |
| 4XZ5 | Q31FD6 | 1 | 1 |  | 4QAX | Q2G029 | 2 | 2 |
| 4BUB | Q9WY79 | 1 | 1 |  | 1E3I | Q9QYY9 | 2 | 2 |
| 5OAW | A0A0S7E9S6 | 1 | 1 |  | 8ACK | Q9HZQ8 | 2 | 2 |
| 1J5S | Q9WXR9 | 1 | 0 |  | 4QNK | O23346 | 2 | 2 |
| 3IEC | Q7KZI7 | 1 | 0 |  | 2ZOG | Q9D1A2 | 2 | 2 |
| 6K63 | A6TBN1 | 1 | 1 |  | 8CR4 | P14756 | 2 | 2 |
| 4CYM | Q13637 | 1 | 1 |  | 6T5K | L0FY79 | 2 | 2 |
| 7WIK | A0R1E6 | 1 | 0 |  | 7YPR | A0A1D1VU85 | 2 | 2 |
| 7WKL | P80402 | 1 | 1 |  | 2P3N | O33832 | 2 | 1 |
| 8DYQ | Q50940 | 1 | 1 |  | 1EE2 | P00328 | 2 | 2 |
| 8DQ3 | A0A142G3G1 | 1 | 1 |  | 7YHA | Q79MP6 | 2 | 2 |
| 8E2S | P68398 | 1 | 1 |  | 8CQM | P33378 | 2 | 2 |
| 8E40 | Q7YR23 | 1 | 1 |  | 2WOE | P14300 | 2 | 1 |
| 8EYO | Q16798 | 1 | 0 |  | 6YJH | P9WML9 | 2 | 2 |
| 8H2A | T2KM87 | 1 | 1 |  | 2I9U | Q97MB6 | 2 | 2 |
| 8H2B | G0L712 | 1 | 1 |  | 2UU7 | Q8HZM5 | 2 | 1 |
| 8HFD | A0A1V3VVF3 | 1 | 1 |  | 2GDQ | O06741 | 2 | 0 |
| 8IFZ | A0A6J0Z472 | 1 | 0 |  | 1RL4 | Q8I372 | 2 | 2 |
| 8HMO | A0A0H4P0S7 | 1 | 1 |  | 4C1D | Q9K2N0 | 2 | 2 |
| 8HZ4 | A9W9X0 | 1 | 0 |  | 4JN6 | P9WMK5 | 2 | 1 |
| 8HZ5 | A9WKH8 | 1 | 0 |  | 1RK6 | Q9AGH8 | 2 | 2 |
| 8IM3 | P32754 | 1 | 1 |  | 1RTQ | Q01693 | 2 | 2 |
| 8QHP | Q8U227 | 1 | 1 |  | 4C1H | P04190 | 2 | 2 |
| 8SSG | D3DIV8 | 1 | 0 |  | 4G7F | Q4DZ98 | 2 | 2 |
| 8DI0 | G8UJW8 | 1 | 0 |  | 7OML | O34824 | 2 | 2 |
| 7WIY | Q8IWU9 | 1 | 1 |  | 1LQY | O31410 | 2 | 2 |
| 8CH4 | D3WZ86 | 1 | 1 |  | 4FZX | P0AEK0 | 2 | 0 |
| 8BCW | A0A3G1AUL2 | 1 | 0 |  | 4FUK | Q4FKC0 | 2 | 2 |
| 7XEB | F7IZI6 | 1 | 1 |  | 3LUB | Q5LE76 | 2 | 2 |
| 7XTJ | A0A023J5W7 | 1 | 0 |  | 7D7P | F2TZN0 | 2 | 2 |
| 7Y7O | P67136 | 1 | 1 |  | 1QXY | P0A078 | 2 | 2 |
| 7YH5 | P96379 | 1 | 1 |  | 4LEF | P45548 | 2 | 2 |
| 7YIX | P05186 | 1 | 1 |  | 4GXW | Q0B6Q2 | 2 | 2 |
| 7Z5H | Q68EN5 | 1 | 1 |  | 5XVB | A0A3B6UEP7 | 2 | 0 |
| 7ZBH | O51729 | 1 | 1 |  | 4B28 | Q166H0 | 2 | 2 |
| 8A6W | P9WPL5 | 1 | 0 |  | 6DKH | P39346 | 2 | 0 |
| 8AC5 | Q6CGY9 | 1 | 0 |  | 7CDH | A0A1B0VPV0 | 2 | 2 |
| 8ACU | O54408 | 1 | 1 |  | 1IYX | Q8GR70 | 2 | 2 |
| 8ANP | Q5ZXN5 | 1 | 1 |  | 3M6R | Q9FUZ2 | 2 | 2 |
| 8AQ0 | Q6DTN4 | 1 | 1 |  | 7CWE | P09467 | 2 | 2 |
| 8ARP | P24783 | 1 | 1 |  | 3ZU0 | P44569 | 2 | 2 |
| 8B3C | Q96562 | 1 | 0 |  | 5LWZ | Q4WMJ8 | 2 | 2 |
| 8B4M | D5SK09 | 1 | 0 |  | 1T5J | Q58588 | 2 | 1 |
| 8BGO | A0A160VQZ8 | 1 | 1 |  | 7E2P | A0A7D5V839 | 2 | 0 |
| 6LP5 | D2JIV0 | 1 | 1 |  | 4HPN | A9CEQ8 | 2 | 2 |
| 1VG8 | P09527 | 1 | 1 |  | 4H9G | P60338 | 2 | 2 |
| 1GTI | P19157 | 1 | 0 |  | 2AYI | P42778 | 2 | 2 |
| 3ENO | Q9HLA5 | 1 | 1 |  | 1TAZ | Q01064 | 2 | 2 |
| 5CXK | A0A086SLX8 | 1 | 1 |  | 4CA7 | Q10714 | 2 | 1 |
| 4Z6K | Q8GIX7 | 1 | 1 |  | 4QGM | Q81MI9 | 2 | 2 |
| 2OOG | A0A0H3K7S1 | 1 | 1 |  | 5GUE | C9K1X5 | 2 | 1 |
| 6CTY | Q8ZFU4 | 1 | 1 |  | 1PV9 | P81535 | 2 | 2 |
| 1KFI | P47244 | 1 | 1 |  | 7NLM | Q02RJ6 | 2 | 2 |
| 6AE3 | Q9WV60 | 1 | 0 |  | 4ENL | P00924 | 2 | 2 |
| 3T80 | Q8ZMF7 | 1 | 1 |  | 4EJ6 | Q92PZ3 | 2 | 0 |
| 2PNQ | O88483 | 1 | 1 |  | 1PDZ | P56252 | 2 | 2 |
| 3AMI | F2Z284 | 1 | 0 |  | 1W6T | Q97QS2 | 2 | 2 |
| 2QJS | P52700 | 1 | 1 |  | 2PZI | P9WI73 | 2 | 0 |
| 2P0M | P12530 | 1 | 1 |  | 1P6O | Q12178 | 2 | 1 |
| 8E8W | A0A411MR89 | 1 | 1 |  | 1P0F | O57380 | 2 | 2 |
| 6VLC | A0A0U1RGY0 | 1 | 0 |  | 4EEZ | D2BLA0 | 2 | 2 |
| 2Q01 | Q9A874 | 1 | 0 |  | 4E9A | Q672W7 | 2 | 2 |
| 5KVP | O54308 | 1 | 1 |  | 4E19 | Q9HSF6 | 2 | 2 |
| 4C8R | O75936 | 1 | 1 |  | 1O98 | Q9X519 | 2 | 2 |
| 6U8J | A0A2H0ZWN3 | 1 | 0 |  | 4DR8 | A0A0H3JZJ4 | 2 | 2 |
| 1XV2 | A0A0H3JS15 | 1 | 1 |  | 3OTR | B9PH47 | 2 | 0 |
| 5N1Q | A0A247D6X3 | 1 | 0 |  | 1Q0E | P00442 | 2 | 2 |
| 2VUN | Q0QLE9 | 1 | 1 |  | 4F0R | Q7NZ90 | 2 | 2 |
| 5N5F | D0LZ73 | 1 | 0 |  | 1O0X | Q9X1I7 | 2 | 0 |
| 3VHX | P62331 | 1 | 1 |  | 7CF6 | A0A1B0VPV0 | 2 | 2 |
| 2OWO | P15042 | 1 | 1 |  | 4QR8 | P21165 | 2 | 2 |
| 8G5X | Q5NEJ8 | 1 | 0 |  | 5TI1 | Q144Z1 | 2 | 2 |
| 7VB3 | P93243 | 1 | 1 |  | 4COB | Q9I234 | 2 | 2 |
| 5MDN | A3MV07 | 1 | 1 |  | 6Z42 | E1V3M3 | 2 | 2 |
| 6SLF | Q8GGD4 | 1 | 1 |  | 4FAI | Q86PD7 | 2 | 1 |
| 1UMY | O09171 | 1 | 1 |  | 1N21 | O81192 | 2 | 2 |
| 3UHJ | Q92MR2 | 1 | 1 |  | 1N5N | Q9I7A8 | 2 | 2 |
| 2IMA | Q84GJ4 | 1 | 1 |  | 7ESR | P73270 | 2 | 2 |
| 1U10 | P0C0T5 | 1 | 1 |  | 1NPC | P05806 | 2 | 1 |
| 3BQB | Q97UA0 | 1 | 1 |  | 6J36 | A0A223MA21 | 2 | 0 |
| 4BXF | Q8IUF8 | 1 | 1 |  | 4D1T | Q840P9 | 2 | 2 |
| 5UAM | A0A1W2VMZ5 | 1 | 0 |  | 7W1F | Q9I4L1 | 2 | 2 |
| 4GME | Q9AAR4 | 1 | 1 |  | 7EUN | D2Z025 | 2 | 2 |
| 1GKR | P81006 | 1 | 1 |  | 4D4Z | Q9BU89 | 2 | 2 |
| 4D28 | Q9LDI3 | 1 | 0 |  | 1U3T | P07327 | 2 | 2 |
| 2PHD | Q67FT0 | 1 | 1 |  | 1Y6H | Q93LE9 | 2 | 2 |
| 1NTO | P39462 | 1 | 1 |  | 1U3U | P00325 | 2 | 2 |
| 6C45 | Q15181 | 1 | 0 |  | 3TQP | Q83B44 | 2 | 2 |
| 8CR2 | O43681 | 1 | 0 |  | 2DKD | Q9P4V2 | 2 | 2 |
| 4CNR | P00921 | 1 | 1 |  | 8OFE | C3SRA2 | 2 | 2 |
| 5SWC | Q54735 | 1 | 1 |  | 5NQ9 | A0A2H4A2Q2 | 2 | 2 |
| 3C8Z | A0QZY0 | 1 | 1 |  | 2Y9W | C7FF04 | 2 | 2 |
| 1OB5 | Q01698 | 1 | 1 |  | 2CQZ | O58085 | 2 | 2 |
| 6FAD | Q96SB4 | 1 | 0 |  | 7SXJ | P49841 | 2 | 0 |
| 3ZUK | O53649 | 1 | 1 |  | 4MKS | Q042F4 | 2 | 2 |
| 4F7K | E1ACR6 | 1 | 0 |  | 3OLP | Q8ZQW9 | 2 | 2 |
| 5C0Y | Q12149 | 1 | 0 |  | 8ILI | P42494 | 2 | 2 |
| 4EWT | A0A0H2WZV8 | 1 | 1 |  | 1VJ0 | Q9WYR7 | 2 | 2 |
| 4ZDA | A0QSZ3 | 1 | 1 |  | 1ITU | P16444 | 2 | 2 |
| 2C00 | P37798 | 1 | 0 |  | 6TZ8 | O42773 | 2 | 2 |
| 5NFN | P0C870 | 1 | 1 |  | 6YWN | G2R014 | 2 | 1 |
| 6CSJ | A0A150JSL8 | 1 | 0 |  | 1J6O | Q9WZD5 | 2 | 0 |
| 3I6T | Q28SI7 | 1 | 1 |  | 1WY2 | O58885 | 2 | 2 |
| 2RJB | A0A0H2UZX2 | 1 | 1 |  | 6AH8 | A0A1I7CHQ2 | 2 | 2 |
| 3IV8 | O32445 | 1 | 1 |  | 8K5Y | P14780 | 2 | 2 |
| 4I1O | Q9H0U4 | 1 | 1 |  | 6XMR | A8WBX8 | 2 | 2 |
| 7P2F | Q92Z29 | 1 | 1 |  | 1GYC | Q12718 | 2 | 2 |
| 3MEN | Q3JUN4 | 1 | 1 |  | 4AS5 | O55023 | 2 | 2 |
| 6DEF | G0SFF0 | 1 | 1 |  | 1Y13 | C6KTB6 | 2 | 2 |
| 4CHG | P9WF97 | 1 | 1 |  | 4LIM | P10363 | 2 | 0 |
| 4D7K | K4RFM2 | 1 | 0 |  | 1XX7 | Q8U3R1 | 2 | 2 |
| 1LL0 | P13280 | 1 | 0 |  | 7BGN | A0A072U2X9 | 2 | 2 |
| 7BV5 | P47058 | 1 | 1 |  | 5OEZ | O97193 | 2 | 0 |
| 2F7L | Q976E4 | 1 | 0 |  | 1XWY | P27859 | 2 | 1 |
| 6GWU | Q5AJ71 | 1 | 1 |  | 1H2B | Q9Y9P9 | 2 | 2 |
| 8EVO | P9WKZ1 | 1 | 1 |  | 1HP1 | P07024 | 2 | 2 |
| 5OM9 | P15085 | 1 | 1 |  | 4S17 | A1A1Z1 | 2 | 1 |
| 3GYR | Q53692 | 1 | 1 |  | 7TL8 | Q5HHP2 | 2 | 2 |
| 8GHE | P18054 | 1 | 1 |  | 1XP3 | Q81LV1 | 2 | 2 |
| 6TV6 | P37570 | 1 | 0 |  | 4LA2 | D0CY60 | 2 | 2 |
| 6Q2D | A5UMY5 | 1 | 0 |  | 1XSO | P15107 | 2 | 2 |
| 4BPT | Q5ZS72 | 1 | 0 |  | 4K89 | A7LI11 | 2 | 1 |
| 2P88 | Q81IL5 | 1 | 1 |  | 6ZAT | H0SLX7 | 2 | 2 |
| 8D19 | O43813 | 1 | 1 |  | 1YEY | Q8P3K2 | 2 | 2 |
| 6EUO | J3BZS6 | 1 | 1 |  | 4A7U | P00441 | 2 | 2 |
| 3RT0 | Q9CAJ0 | 1 | 1 |  | 4J3R | Q2UNF9 | 2 | 2 |
| 6PXU | Q8IXK2 | 1 | 1 |  | 3ZK4 | Q8VX11 | 2 | 2 |
| 6J6T | Q8GXJ1 | 1 | 1 |  | 7AO3 | C9K1X5 | 2 | 1 |
| 2BB0 | P42084 | 1 | 1 |  | 7ARW | Q9NX46 | 2 | 2 |
| 5KSR | Q9PF20 | 1 | 1 |  | 4IR8 | B9PW60 | 2 | 0 |
| 6MM7 | P05132 | 1 | 0 |  | 1V33 | O57934 | 2 | 0 |
| 6QPZ | I6NAW4 | 1 | 1 |  | 1U8X | P54716 | 2 | 1 |
| 5M4X | Q9HK01 | 1 | 0 |  | 3TOY | A4YVM8 | 2 | 2 |
| 5KVU | O53611 | 1 | 0 |  | 1SZZ | Q93LE9 | 2 | 2 |
| 3I12 | P0A1F0 | 1 | 0 |  | 5X49 | Q9NQH7 | 2 | 2 |
| 4OKO | A0Q494 | 1 | 1 |  | 4ILK | A0A0H2V9Q5 | 2 | 2 |
| 1NKQ | P53889 | 1 | 1 |  | 4AQL | Q9Y2T3 | 2 | 2 |
| 3OID | P71079 | 1 | 0 |  | 2XSX | P13929 | 2 | 2 |
| 6LJG | Q70MM3 | 1 | 1 |  | 5DM3 | Q1QZR8 | 2 | 0 |
| 1O0S | P27443 | 1 | 0 |  | 4J6O | A3DJ38 | 2 | 1 |
| 6JWP | Q00582 | 1 | 1 |  | 4BP0 | Q8G9Q0 | 2 | 2 |
| 5ET6 | O00757 | 1 | 0 |  | 4J6V | B2ZB02 | 2 | 1 |
| 1IAX | P18485 | 1 | 0 |  | 7SKL | P81177 | 2 | 1 |
| 6JUR | A0QR77 | 1 | 1 |  | 4K25 | P43122 | 2 | 1 |
| 3IJF | P9WPH3 | 1 | 1 |  | 7E15 | Q5JGL0 | 2 | 0 |
| 7DWQ | B0C474 | 1 | 0 |  | 1WN1 | O58691 | 2 | 2 |
| 4RFL | Q58122 | 1 | 1 |  | 6ZEP | Q2U1F3 | 2 | 2 |
| 6YVT | Q9GZT9 | 1 | 1 |  | 8SQR | Q2YKI4 | 2 | 2 |
| 1W2Z | Q43077 | 1 | 1 |  | 7PK5 | P21514 | 2 | 0 |
| 5YLN | A0A0H2ZRI0 | 1 | 1 |  | 3CB3 | Q12GE3 | 2 | 2 |
| 4LWZ | P62491 | 1 | 1 |  | 1LRY | Q9I7A8 | 2 | 2 |
| 4A69 | O15379 | 1 | 1 |  | 3AUO | Q5SJ64 | 2 | 2 |
| 3NXL | Q39KL8 | 1 | 1 |  | 4CPD | B2ZRE3 | 2 | 2 |
| 4C5Y | A2R2V4 | 1 | 1 |  | 1W0H | Q8IV48 | 2 | 2 |
| 3OWO | P0DJA2 | 1 | 1 |  | 4JE6 | Q9FV53 | 2 | 2 |
| 2JKA | G8JZS4 | 1 | 1 |  | 1VFL | P56658 | 2 | 2 |
| 5FHH | Q9H611 | 1 | 0 |  | 4S2T | O44750 | 2 | 2 |
| 5OUW | Q0I9X8 | 1 | 0 |  | 1U3W | P00326 | 2 | 2 |
| 4PXB | Q8VXY9 | 1 | 1 |  | 3SW8 | Q939R9 | 2 | 2 |
| 1JWQ | Q9LCR3 | 1 | 1 |  | 2JHF | P00327 | 2 | 2 |
| 6BWY | O13988 | 1 | 1 |  | 5BOF | O69174 | 2 | 2 |
| 4NUR | F2WP51 | 1 | 1 |  | 3H7J | P39639 | 2 | 2 |
| 7CY8 | A0A2K3D5Z7 | 1 | 1 |  | 5VKT | C5XC49 | 2 | 2 |
| 4KIR | Q5DLU2 | 1 | 1 |  | 5VI6 | Q9BY41 | 2 | 1 |
| 3GM5 | Q8RCQ6 | 1 | 0 |  | 5VEO | Q9EQG7 | 2 | 2 |
| 4C4O | O42703 | 1 | 1 |  | 5ZFS | A0A1L7NQ96 | 2 | 1 |
| 3KYH | O13297 | 1 | 0 |  | 5ZGZ | C7C422 | 2 | 2 |
| 3HI7 | P19801 | 1 | 1 |  | 3GB0 | Q731F0 | 2 | 0 |
| 1VKL | P00949 | 1 | 1 |  | 3G6N | Q842S4 | 2 | 2 |
| 3Q1G | Q9AIX7 | 1 | 1 |  | 5VPU | A0A059ZPG7 | 2 | 2 |
| 7TRM | Q13490 | 1 | 1 |  | 3G5K | Q9HBH1 | 2 | 2 |
| 4UOR | Q8Y8H6 | 1 | 1 |  | 1Q3K | P83772 | 2 | 2 |
| 5DCX | P03132 | 1 | 1 |  | 6NBK | W8YSI5 | 2 | 2 |
| 1G5C | Q50565 | 1 | 1 |  | 3FWX | Q9KVU3 | 2 | 2 |
| 3ANM | P45568 | 1 | 0 |  | 3FM3 | Q8SR45 | 2 | 2 |
| 6Q3O | P00918 | 1 | 1 |  | 3E0L | Q9Y2T3 | 2 | 2 |
| 3RAW | P49761 | 1 | 0 |  | 3FED | Q9Y3Q0 | 2 | 2 |
| 3NV9 | Q9NH04 | 1 | 0 |  | 6A47 | Q9R1A9 | 2 | 2 |
| 6Y9D | A0A059ZPP5 | 1 | 1 |  | 6HC7 | P25152 | 2 | 2 |
| 7YBU | P05165 | 1 | 0 |  | 3G1P | P16692 | 2 | 2 |
| 1CJX | P80064 | 1 | 1 |  | 6AEK | P06802 | 2 | 2 |
| 1RQC | Q8I372 | 1 | 1 |  | 7CQL | A0A369R1N0 | 2 | 0 |
| 8GOA | P0A9S5 | 1 | 1 |  | 2QVW | A8BQJ3 | 2 | 2 |
| 1JDI | P08203 | 1 | 1 |  | 3KMH | Q8X5Q7 | 2 | 2 |
| 4WW0 | O67077 | 1 | 1 |  | 3KI9 | Q5HF23 | 2 | 2 |
| 3DLA | P9WJJ3 | 1 | 0 |  | 5WRO | P15007 | 2 | 2 |
| 4CZT | Q93VD3 | 1 | 0 |  | 3DKH | Q70KY3 | 2 | 2 |
| 3ZVH | Q05514 | 1 | 1 |  | 6ODB | Q9BY41 | 2 | 1 |
| 1QPR | P9WJJ7 | 1 | 0 |  | 2WYM | P39300 | 2 | 2 |
| 4N7T | Q8DTU0 | 1 | 1 |  | 5EQA | G7J7Q5 | 2 | 0 |
| 1KUH | P56406 | 1 | 1 |  | 5XEV | Q9RX37 | 2 | 2 |
| 3NH2 | P30014 | 1 | 0 |  | 3HR1 | Q9QYJ6 | 2 | 2 |
| 2YHE | F8KAY7 | 1 | 1 |  | 4BAX | Q9X958 | 2 | 0 |
| 2IS1 | P03018 | 1 | 0 |  | 5XGW | Q484B6 | 2 | 2 |
| 2FL0 | P22759 | 1 | 1 |  | 1F1G | P00445 | 2 | 2 |
| 4TLF | Q9I0N5 | 1 | 1 |  | 3IP1 | Q9WYP3 | 2 | 2 |
| 6KU3 | Q8S0S6 | 1 | 0 |  | 5IN2 | Q07449 | 2 | 2 |
| 2UZ3 | Q9PPP5 | 1 | 0 |  | 4IS4 | O04998 | 2 | 0 |
| 6JCM | O07431 | 1 | 0 |  | 3IGZ | Q86N96 | 2 | 2 |
| 2Y74 | Q16853 | 1 | 1 |  | 5XSP | A0A0U1MUE2 | 2 | 2 |
| 2B3J | Q99W51 | 1 | 1 |  | 4OJA | I3V7W8 | 2 | 2 |
| 3D47 | Q8ZNF9 | 1 | 1 |  | 6LL7 | L8AXY8 | 2 | 2 |
| 3MDW | Q9HU77 | 1 | 1 |  | 5T8Z | A0A0H3KPJ9 | 2 | 2 |
| 1J38 | Q10714 | 1 | 1 |  | 3GRI | P65907 | 2 | 1 |
| 3AEU | P26164 | 1 | 0 |  | 1QCN | P35505 | 2 | 2 |
| 3GZE | A8J7D3 | 1 | 1 |  | 6DRE | A8GG79 | 2 | 1 |
| 3RPL | P73922 | 1 | 1 |  | 1N1Z | O81192 | 2 | 2 |
| 4UOV | E8T502 | 1 | 1 |  | 4ZWP | Q44238 | 2 | 2 |
| 6STY | Q9Y3B8 | 1 | 0 |  | 5JVI | P00800 | 2 | 2 |
| 4OJ8 | Q9XB59 | 1 | 1 |  | 4PXD | P77425 | 2 | 1 |
| 4EME | Q8I2J3 | 1 | 1 |  | 3B0X | Q5SJ64 | 2 | 2 |
| 6DXS | G2IQQ5 | 1 | 1 |  | 3AV0 | Q58719 | 2 | 0 |
| 1IS7 | P22288 | 1 | 0 |  | 3QN0 | P65870 | 2 | 2 |
| 2XH0 | P00924 | 1 | 1 |  | 2D3C | P38561 | 2 | 2 |
| 2FVM | Q9P903 | 1 | 1 |  | 5J46 | A0A0H3KB98 | 2 | 2 |
| 4XOI | P61586 | 1 | 1 |  | 5IL3 | Q40577 | 2 | 2 |
| 3HHS | Q25519 | 1 | 1 |  | 2ZSG | Q9WXP9 | 2 | 2 |
| 1EAK | P08253 | 1 | 1 |  | 5I2B | B1FPK4 | 2 | 2 |
| 2NX9 | Q6A1F6 | 1 | 1 |  | 8BAH | P49959 | 2 | 2 |
| 1VLJ | Q9WZS7 | 1 | 1 |  | 6ESL | Q5ZRR6 | 2 | 2 |
| 7B6N | P22188 | 1 | 0 |  | 6EVG | A6X5N0 | 2 | 1 |
| 3NVL | Q38AH1 | 1 | 1 |  | 5HRB | P42494 | 2 | 1 |
| 7PLC | Q9A9Z1 | 1 | 1 |  | 4TUI | Q58719 | 2 | 0 |
| 5I4M | A4JQA0 | 1 | 1 |  | 5IQK | A0A059Q5E8 | 2 | 2 |
| 1OIH | Q9WWU5 | 1 | 0 |  | 6DJA | P14488 | 2 | 2 |
| 6K1G | A0A443VNT1 | 1 | 1 |  | 5KOB | Q13XB1 | 2 | 2 |
| 3Q3L | H2L2L6 | 1 | 1 |  | 3BU7 | Q5LLB1 | 2 | 2 |
| 6WC8 | F2WR52 | 1 | 0 |  | 3E3U | P9WIJ3 | 2 | 2 |
| 3UXL | P11444 | 1 | 1 |  | 5OVO | A7XNI2 | 2 | 1 |
| 2PCR | O67791 | 1 | 0 |  | 5O9X | A0A0S7E9S6 | 2 | 0 |
| 3MWP | P13699 | 1 | 0 |  | 5O5T | Q04609 | 2 | 2 |
| 1INO | P0A7A9 | 1 | 1 |  | 3UJ2 | B0MAG5 | 2 | 2 |
| 1N6O | P20339 | 1 | 1 |  | 5NRY | Q4WMJ8 | 2 | 2 |
| 3RDR | P39800 | 1 | 1 |  | 5GWO | Q6L5H6 | 2 | 1 |
| 4WQ5 | P05852 | 1 | 1 |  | 5NDE | Q8G9Q0 | 2 | 2 |
| 1FRF | P18188 | 1 | 1 |  | 6BU2 | L7N6B1 | 2 | 0 |
| 3TEN | G0WXL9 | 1 | 1 |  | 5LWX | A2QS62 | 2 | 2 |
| 1K1D | Q45515 | 1 | 1 |  | 6FOL | O14618 | 2 | 2 |
| 3GT7 | Q2LQE8 | 1 | 0 |  | 6C5C | Q5AME2 | 2 | 0 |
| 3OSL | Q2KIG3 | 1 | 1 |  | 5J04 | Q5N3P4 | 2 | 2 |
| 3TWA | B5R541 | 1 | 1 |  | 6CAZ | Q5ZSC4 | 2 | 2 |
| 5M6B | C7FF05 | 1 | 1 |  | 1ESO | P0AGD1 | 2 | 2 |
| 1HV5 | Q02853 | 1 | 1 |  | 3LN7 | Q9CM00 | 2 | 0 |
| 3RDE | P16469 | 1 | 1 |  | 2GMN | Q89GW5 | 2 | 2 |
| 4KVY | Q9UR08 | 1 | 1 |  | 3CAW | Q6MQC7 | 2 | 2 |
| 6WN6 | P76044 | 1 | 1 |  | 6CPU | Q8NJP9 | 2 | 2 |
| 6AR1 | E2GM63 | 1 | 0 |  | 1TK0 | P00581 | 2 | 2 |
| 3VNI | B8I944 | 1 | 1 |  | 2Z00 | Q5SK67 | 2 | 2 |
| 5ZIG | E0RU15 | 1 | 0 |  | 1F52 | P0A1P6 | 2 | 2 |
| 4QJ4 | P21279 | 1 | 1 |  | 5WLY | P43341 | 2 | 2 |
| 7X12 | P48163 | 1 | 1 |  | 3QU1 | Q9KN16 | 2 | 2 |
| 3RNF | Q6IV66 | 1 | 1 |  | 3QE3 | P07846 | 2 | 2 |
| 5O6Y | Q81EK9 | 1 | 0 |  | 1WX5 | Q83WS2 | 2 | 0 |
| 4YYE | P07236 | 1 | 1 |  | 3QBE | P9WPX9 | 2 | 1 |
| 2P76 | A0A0H3JTK7 | 1 | 0 |  | 8GSQ | P00441 | 2 | 1 |
| 2CDB | O93715 | 1 | 1 |  | 3U04 | Q2GI30 | 2 | 2 |
| 4G3M | P17618 | 1 | 1 |  | 8TDW | Q9Y3Z3 | 2 | 2 |
| 8FEC | P17612 | 1 | 0 |  | 1WQA | O58651 | 2 | 2 |
| 1OS7 | P37610 | 1 | 1 |  | 3GIQ | A0A0H3LXD5 | 2 | 2 |
| 4KYI | Q5ZRP9 | 1 | 0 |  | 5VG3 | O34714 | 2 | 2 |
| 5T8Y | P97030 | 1 | 0 |  | 4YU9 | Q16769 | 2 | 1 |
| 3T81 | Q7CUX4 | 1 | 1 |  | 3PU7 | P14831 | 2 | 2 |
| 3HOA | Q72GY3 | 1 | 0 |  | 8HCH | Q91XB0 | 2 | 1 |
| 4OEC | Q5JGZ3 | 1 | 1 |  | 4CI0 | D9PYF9 | 2 | 2 |
| 5OU5 | P16243 | 1 | 0 |  | 3PKA | P9WK19 | 2 | 2 |
| 1MWU | Q93IC2 | 1 | 0 |  | 2V8V | Q96W94 | 2 | 2 |
| 1YDO | O34873 | 1 | 0 |  | 3PB6 | Q9NXS2 | 2 | 1 |
| 2UYV | P32169 | 1 | 1 |  | 3PAO | Q9I6Y4 | 2 | 2 |
| 5OET | A0A183C5H8 | 1 | 0 |  | 7U9N | P00327 | 2 | 2 |
| 3CF4 | Q46G04 | 1 | 0 |  | 5YN3 | Q9P8C9 | 2 | 2 |
| 3T6B | Q9NY33 | 1 | 0 |  | 3QY7 | P96717 | 2 | 2 |
| 4QRO | Q12BV1 | 1 | 1 |  | 4KNT | Q82VX5 | 2 | 2 |
| 6DA6 | C6ZCR8 | 1 | 1 |  | 3SQR | H8ZRU2 | 2 | 2 |
| 3QYR | Q73VV7 | 1 | 0 |  | 5GN9 | Q26710 | 2 | 2 |
| 4HCB | P04995 | 1 | 0 |  | 3SI2 | Q9CYK2 | 2 | 1 |
| 2DVM | O59029 | 1 | 0 |  | 3T2C | B1YAL1 | 2 | 2 |
| 6I3J | Q12737 | 1 | 1 |  | 3TAV | B1MGB2 | 2 | 2 |
| 1N9E | Q96X16 | 1 | 1 |  | 3RYS | A1R3U3 | 2 | 2 |
| 2JSD | O60882 | 1 | 1 |  | 4RUH | Q96KP4 | 2 | 2 |
| 5ZMD | Q9C0B1 | 1 | 1 |  | 3RVA | F8UVJ4 | 2 | 2 |
| 4FLU | P0CL76 | 1 | 1 |  | 3T6W | I1SB14 | 2 | 2 |
| 7CV1 | Q9NWX6 | 1 | 0 |  | 3TN4 | Q5KZU5 | 2 | 2 |
| 5VJA | O43293 | 1 | 0 |  | 4IXV | P78540 | 2 | 2 |
| 3N1R | Q62226 | 1 | 1 |  | 1DJI | P10688 | 2 | 2 |
| 3NYN | P43250 | 1 | 0 |  | 3RMU | Q96PE7 | 2 | 2 |
| 4ZG5 | B2S5B9 | 1 | 1 |  | 3TR8 | Q83C93 | 2 | 2 |
| 7TC8 | P18798 | 1 | 0 |  | 1PL7 | Q00796 | 2 | 2 |
| 2GLJ | Q97K30 | 1 | 1 |  | 2DQB | Q5SL81 | 2 | 2 |
| 8D45 | Q93088 | 1 | 0 |  | 3RCM | Q88KH9 | 2 | 1 |
| 5CY4 | V5VGJ9 | 1 | 1 |  | 3HDP | Q97H22 | 2 | 2 |
| 3OUU | Q0P8W7 | 1 | 1 |  | 4PV4 | A0A2S9PFE9 | 2 | 2 |
| 6MEV | Q6NYC1 | 1 | 1 |  | 2PP1 | Q8ZL58 | 2 | 2 |
| 2P50 | P0AF18 | 1 | 1 |  | 3UVC | P39900 | 2 | 2 |
| 6NS4 | I1REW2 | 1 | 1 |  | 4RVP | A0A0A0QA66 | 2 | 1 |
| 5O7B | Q55535 | 1 | 0 |  | 4H1Z | Q92ZS5 | 2 | 2 |
| 4U7D | P46063 | 1 | 0 |  | 3LY0 | Q3IZQ3 | 2 | 2 |
| 2JFC | O68601 | 1 | 1 |  | 3LX3 | A5K2B2 | 2 | 2 |
| 7CJ8 | A0A172U6X0 | 1 | 1 |  | 4YWS | A9WCM4 | 2 | 2 |
| 5MLK | P96890 | 1 | 0 |  | 1HO5 | P07024 | 2 | 2 |
| 7NEY | G9NLP8 | 1 | 1 |  | 3ZTV | P44569 | 2 | 2 |
| 2RGW | Q58976 | 1 | 0 |  | 3LN6 | Q8DXM9 | 2 | 0 |
| 4A3W | O29917 | 1 | 1 |  | 8FF8 | P49841 | 2 | 0 |
| 3WBH | B5BP20 | 1 | 1 |  | 1M2X | O08498 | 2 | 2 |
| 1RM8 | P51512 | 1 | 1 |  | 1Z8L | Q04609 | 2 | 2 |
| 2OVL | Q9RKF7 | 1 | 0 |  | 5WCK | Q9K578 | 2 | 2 |
| 5KQC | Q2G1F1 | 1 | 1 |  | 4L05 | P15453 | 2 | 2 |
| 5BQO | Q97W22 | 1 | 0 |  | 3OWG | P23371 | 2 | 0 |
| 5D4B | P09838 | 1 | 0 |  | 3L9Y | P82205 | 2 | 2 |
| 3U9T | Q9I299 | 1 | 0 |  | 6JU6 | A0A1S9DK56 | 2 | 0 |
| 6HEG | P37024 | 1 | 0 |  | 3L6N | A4GRB2 | 2 | 2 |
| 6O4N | B0BA40 | 1 | 1 |  | 1XEN | P0A6K3 | 2 | 2 |
| 1WUR | Q5SH52 | 1 | 1 |  | 6OHC | P76641 | 2 | 2 |
| 5CFJ | C0H4F3 | 1 | 0 |  | 8DNU | P15104 | 2 | 1 |
| 2XS4 | D0EM77 | 1 | 1 |  | 2XAU | P53131 | 2 | 1 |
| 6OPM | A0A0F8IEL4 | 1 | 0 |  | 4R85 | A0A0E1CHI1 | 2 | 2 |
| 6WNI | U5CJP3 | 1 | 0 |  | 4IP5 | Q1GLV3 | 2 | 2 |
| 7PTJ | O69787 | 1 | 1 |  | 3MPG | Q81WF0 | 2 | 2 |
| 4JKM | Q8XP19 | 1 | 0 |  | 6EZM | P06633 | 2 | 2 |
| 4OA5 | Q2GKC7 | 1 | 0 |  | 3NZT | Q5NHS8 | 2 | 0 |
| 5JIG | O94580 | 1 | 1 |  | 3NQX | A1DRD5 | 2 | 2 |
| 1GTT | P37352 | 1 | 1 |  | 3NQW | Q9VAM9 | 2 | 2 |
| 1R44 | Q06241 | 1 | 1 |  | 5EWA | P52699 | 2 | 2 |
| 7CYI | A0A2U1Q018 | 1 | 1 |  | 3NG0 | P77961 | 2 | 2 |
| 4L6T | Q8GAV4 | 1 | 1 |  | 8W6X | P21852 | 2 | 0 |
| 5JMY | P08473 | 1 | 1 |  | 6FWH | L8H477 | 2 | 2 |
| 5LUZ | Q9BYT8 | 1 | 1 |  | 1JQW | O34667 | 2 | 1 |
| 4LT6 | Q9BWT3 | 1 | 0 |  | 7WI1 | A0A2U8UYM6 | 2 | 2 |
| 1RTU | P00654 | 1 | 0 |  | 3QI3 | O76083 | 2 | 2 |
| 4LRT | D1A3K8 | 1 | 1 |  | 3WID | Q979W2 | 2 | 0 |
| 3I4K | Q8NN12 | 1 | 1 |  | 3MVI | P03958 | 2 | 2 |
| 2UX8 | Q5FYV5 | 1 | 0 |  | 1POX | P37063 | 2 | 0 |
| 2F1D | P34047 | 1 | 1 |  | 7UU5 | M1GSK9 | 2 | 2 |
| 1JKU | P60355 | 1 | 1 |  | 7MBH | P09104 | 2 | 2 |
| 5IG4 | A7T0H5 | 1 | 0 |  | 4XCT | P14780 | 2 | 2 |
| 1T8Q | P09394 | 1 | 1 |  | 3M6I | Q7SI09 | 2 | 2 |
| 3VYL | N0DP12 | 1 | 1 |  | 6ZIJ | A0A0M4FJ81 | 2 | 2 |
| 7LIU | O00571 | 1 | 0 |  | 2YZ3 | Q7BJM5 | 2 | 2 |
| 3ES8 | Q8EMJ9 | 1 | 0 |  | 1A7T | P25910 | 2 | 2 |
| 3I4Q | D0VWZ3 | 1 | 0 |  | 4E4F | C6D9S0 | 2 | 2 |
| 5IWX | Q06756 | 1 | 1 |  | 2OS0 | Q82ZJ0 | 2 | 2 |
| 2EPG | Q5SHE5 | 1 | 0 |  | 4QGE | H2QL32 | 2 | 2 |
| 2DW2 | Q90282 | 1 | 1 |  | 5EGE | Q8BGN3 | 2 | 2 |
| 1NDO | P0A110 | 1 | 1 |  | 2OOD | Q89NG0 | 2 | 2 |
| 5NQA | Q8N4A0 | 1 | 0 |  | 5DJH | P9WKJ1 | 2 | 2 |
| 3JUJ | O25363 | 1 | 0 |  | 1E9I | P0A6P9 | 2 | 2 |
| 4UMP | Q14680 | 1 | 0 |  | 2QJM | A4XF23 | 2 | 2 |
| 5ZY9 | G3FIN0 | 1 | 1 |  | 2W3T | P0A6K3 | 2 | 2 |
| 2A5V | P9WPJ9 | 1 | 1 |  | 6L25 | W8U6D8 | 2 | 2 |
| 3WKS | Q59072 | 1 | 0 |  | 2OKL | Q819K2 | 2 | 2 |
| 3ZFZ | A0A0H3JPA5 | 1 | 0 |  | 2W5Q | Q7A1I3 | 2 | 2 |
| 4A22 | P9WQA3 | 1 | 1 |  | 6L3S | A0A286S0G7 | 2 | 2 |
| 3BOL | Q9WYA5 | 1 | 1 |  | 4Z0U | A7ZHV1 | 2 | 0 |
| 4A5Q | B6A876 | 1 | 0 |  | 6LH7 | Q9KPV1 | 2 | 2 |
| 3GFB | Q5JI69 | 1 | 0 |  | 3MR1 | Q9ZCD3 | 2 | 2 |
| 7LVZ | Q6PDY2 | 1 | 1 |  | 6LL8 | L8AXY8 | 2 | 2 |
| 2ERP | Q9DGB9 | 1 | 1 |  | 7OI1 | P73270 | 2 | 2 |
| 3F9T | Q60358 | 1 | 0 |  | 3ZLG | Q5XD01 | 2 | 0 |
| 3E2D | Q93P54 | 1 | 1 |  | 1ASQ | P37064 | 2 | 2 |
| 1LI5 | P21888 | 1 | 1 |  | 5CWX | Q5H3Z2 | 2 | 2 |
| 7F68 | P01111 | 1 | 1 |  | 5EKW | O23346 | 2 | 0 |
| 5HVG | Q96IY4 | 1 | 1 |  | 2WHG | Q8KRJ3 | 2 | 2 |
| 1EB6 | P46076 | 1 | 1 |  | 5ENV | P00330 | 2 | 2 |
| 4M6R | Q96GX9 | 1 | 1 |  | 3DLJ | Q96KN2 | 2 | 2 |
| 2DVT | Q60GU1 | 1 | 1 |  | 2KFN | P00582 | 2 | 1 |
| 4CQB | O52063 | 1 | 1 |  | 2OS3 | P68771 | 2 | 2 |
| 4IHC | C6CBG9 | 1 | 1 |  | 1RJW | P42328 | 2 | 2 |
| 3W6W | Q2UP46 | 1 | 1 |  | 2WYR | O58255 | 2 | 2 |
| 4GAA | Q6IP81 | 1 | 1 |  | 6KLI | Q2PAJ1 | 2 | 2 |
| 6KTQ | Q4J989 | 1 | 1 |  | 6JEV | B0VNL8 | 2 | 2 |
| 1QQC | Q7SIG7 | 1 | 1 |  | 6JF9 | A0A071LDC0 | 2 | 2 |
| 3B97 | P06733 | 1 | 1 |  | 2Q2L | B2CP37 | 2 | 1 |
| 4DD8 | P78325 | 1 | 1 |  | 5B3R | Q5U807 | 2 | 2 |
| 2QT6 | Q5EBY5 | 1 | 1 |  | 2XAA | Q8KLT9 | 2 | 2 |
| 6JSS | Q5KZU5 | 1 | 1 |  | 6JED | P52699 | 2 | 2 |
| 2Y7F | B0VHH0 | 1 | 1 |  | 5AKR | P25006 | 2 | 2 |
| 2GWC | O23736 | 1 | 1 |  | 1VJ7 | Q54089 | 2 | 1 |
| 1NDR | P81445 | 1 | 1 |  | 3PDK | Q81VN7 | 2 | 0 |
| 4OBX | P49017 | 1 | 0 |  | 5AEB | B5L5V5 | 2 | 2 |
| 2DZD | Q05FZ3 | 1 | 0 |  | 6J2L | P37793 | 2 | 2 |
| 2QKK | O60930 | 1 | 1 |  | 6IUX | P54922 | 2 | 1 |
| 2XJA | P9WJL3 | 1 | 1 |  | 3Q6D | A0A6L7GZS1 | 2 | 2 |
| 1KGN | O69274 | 1 | 1 |  | 3WNQ | A1X808 | 2 | 2 |
| 1SUI | Q40313 | 1 | 1 |  | 2PGF | A5KE01 | 2 | 2 |
| 6FOG | Q6P587 | 1 | 1 |  | 2R8Q | Q6S996 | 2 | 2 |
| 5ZHH | Q8YT10 | 1 | 0 |  | 5CP0 | Q5H3Z2 | 2 | 2 |
| 3QN3 | P42448 | 1 | 1 |  | 6K6T | A0A0H3AJ04 | 2 | 2 |
| 1SMQ | P09938 | 1 | 0 |  | 5CU1 | Q5LT18 | 2 | 2 |
| 4MIT | Q24816 | 1 | 1 |  | 2V3Z | P15034 | 2 | 2 |
| 2VCG | Q70I53 | 1 | 1 |  | 6K3G | Q6V4H0 | 2 | 2 |
| 2DFU | Q5SK43 | 1 | 0 |  | 6IFQ | A0A0H3AJ04 | 2 | 1 |
| 1GUP | P09148 | 1 | 1 |  | 6K4X | G5ELM3 | 2 | 2 |
| 5XOX | P53215 | 1 | 1 |  | 4MM2 | P10363 | 2 | 2 |
| 7KCT | D3DJ42 | 1 | 1 |  | 6KC0 | B6YWB8 | 2 | 1 |
| 3ULK | P05793 | 1 | 1 |  | 3FKY | P32288 | 2 | 0 |
| 1BKH | Q51958 | 1 | 0 |  | 5A87 | Q8GKX2 | 2 | 2 |
| 2FVZ | O14732 | 1 | 0 |  | 6N6A | Q9KV17 | 2 | 0 |
| 4BJU | B0XPI4 | 1 | 1 |  | 1E5D | Q9F0J6 | 2 | 1 |
| 4KVG | P62834 | 1 | 1 |  | 6G1P | H3BCW1 | 2 | 1 |
| 3VTI | Q8RDB0 | 1 | 1 |  | 4IL1 | P0DP29 | 2 | 0 |
| 2B1X | Q9X3R9 | 1 | 1 |  | 6GIT | F6MIW5 | 2 | 2 |
| 7A2G | P07101 | 1 | 1 |  | 3THU | Q1NAJ2 | 2 | 2 |
| 7LLQ | P09960 | 1 | 1 |  | 3GTT | P08228 | 2 | 1 |
| 6ZHT | P52490 | 1 | 0 |  | 6FV4 | A0QU89 | 2 | 2 |
| 4XBH | P08049 | 1 | 1 |  | 2HXT | Q8P3K2 | 2 | 2 |
| 2P0U | Q5I6Y1 | 1 | 0 |  | 2G64 | O02058 | 2 | 2 |
| 4FI4 | B0T0B1 | 1 | 1 |  | 2Y0O | P96578 | 2 | 2 |
| 2FJK | Q703I2 | 1 | 0 |  | 4EWJ | K7N5M7 | 2 | 0 |
| 5AVP | D2S5K0 | 1 | 1 |  | 5H2Q | Q38F42 | 2 | 2 |
| 3TR4 | Q83DR7 | 1 | 1 |  | 4UEJ | P0A9S3 | 2 | 2 |
| 7E2Q | P75189 | 1 | 0 |  | 6FON | O14618 | 2 | 2 |
| 5JK6 | Q54XS1 | 1 | 1 |  | 6LF4 | A0A5Q5ADH9 | 2 | 2 |
| 4YHJ | P32298 | 1 | 0 |  | 4UBQ | Q9KVZ2 | 2 | 2 |
| 1VMG | Q97U11 | 1 | 0 |  | 3LGG | Q9NZK5 | 2 | 2 |
| 3AL5 | A2RUC4 | 1 | 0 |  | 6OH9 | Q07729 | 2 | 2 |
| 2CXI | O73984 | 1 | 0 |  | 7NTM | P00330 | 2 | 2 |
| 1VH2 | Q9RRU8 | 1 | 1 |  | 2GZ5 | P53582 | 2 | 2 |
| 3HQ2 | P50848 | 1 | 1 |  | 6OW7 | Q8DP79 | 2 | 2 |
| 2X98 | B0R9W3 | 1 | 1 |  | 2YB1 | Q7NXD4 | 2 | 2 |
| 4K2B | O07566 | 1 | 0 |  | 5ZON | P95189 | 2 | 2 |
| 5ZL1 | I3IR93 | 1 | 1 |  | 7CPR | P20478 | 2 | 0 |
| 3GV5 | Q9UNA4 | 1 | 0 |  | 5HGW | B1YSH1 | 2 | 2 |
| 6EEM | O82415 | 1 | 0 |  | 2GGE | O06741 | 2 | 2 |
| 6IWQ | Q86SF2 | 1 | 1 |  | 2WRS | Q70E11 | 2 | 1 |
| 4AGU | Q00532 | 1 | 0 |  | 2IFY | Q81X77 | 2 | 2 |
| 8A3N | W8JWW7 | 1 | 1 |  | 3ZXR | P9WN39 | 2 | 2 |
| 8UOY | A3F8V6 | 1 | 0 |  | 2ITB | Q88KV1 | 2 | 2 |
| 7ZRN | A9GK58 | 1 | 1 |  | 6N91 | Q9KNI7 | 2 | 2 |
| 2YAB | Q8VDF3 | 1 | 0 |  | 4WXK | Q4QMV6 | 2 | 2 |
| 2YAY | O15826 | 1 | 1 |  | 2PA6 | Q60173 | 2 | 0 |
| 2YCF | O96017 | 1 | 0 |  | 5HJ9 | Q6TUJ5 | 2 | 2 |
| 2YEX | O14757 | 1 | 0 |  | 6N9M | Q8ZPL9 | 2 | 2 |
| 2YFD | Q9RS96 | 1 | 1 |  | 2WOD | P14300 | 2 | 1 |
| 2Z0M | Q96XQ7 | 1 | 0 |  | 6GIU | P29218 | 2 | 2 |
| 2Y8U | Q5AQQ0 | 1 | 1 |  | 2ZKT | O57742 | 2 | 2 |
| 2Z26 | P05020 | 1 | 1 |  | 1AH7 | P09598 | 3 | 3 |
| 2ZC1 | Q9RVU2 | 1 | 1 |  | 4TW3 | Q7V6D4 | 3 | 2 |
| 2ZC8 | Q5SJX8 | 1 | 0 |  | 6TZ6 | C4YFI3 | 3 | 3 |
| 2ZIU | Q7SXA9 | 1 | 0 |  | 2F6K | F9US96 | 3 | 3 |
| 2ZNC | Q64444 | 1 | 1 |  | 4ZVZ | P53041 | 3 | 3 |
| 2ZNR | Q96FJ0 | 1 | 1 |  | 1K20 | P95765 | 3 | 3 |
| 2ZQB | Q8EE30 | 1 | 0 |  | 7QUL | O87871 | 3 | 3 |
| 2Z3G | P0C2P0 | 1 | 1 |  | 4RPA | P65752 | 3 | 3 |
| 2ZR9 | Q59560 | 1 | 0 |  | 6IKA | D3XFN7 | 3 | 0 |
| 2Y7E | B0VHH0 | 1 | 1 |  | 4XWW | H9CZL7 | 3 | 3 |
| 2XYM | Q99IB8 | 1 | 0 |  | 5AVH | P24300 | 3 | 0 |
| 2WK1 | Q9L9F2 | 1 | 0 |  | 1GQ6 | P0DJQ3 | 3 | 3 |
| 2WQK | O67004 | 1 | 0 |  | 6BRH | Q60710 | 3 | 2 |
| 2WT9 | B0VA03 | 1 | 1 |  | 1BK4 | P00637 | 3 | 3 |
| 2WWO | Q66ED7 | 1 | 1 |  | 2OUI | P35630 | 3 | 3 |
| 2X3C | Q8GMV9 | 1 | 1 |  | 4ZX2 | P53041 | 3 | 3 |
| 2X7M | Q8TXW1 | 1 | 1 |  | 1KHO | Q9RF12 | 3 | 3 |
| 2Y2C | P82974 | 1 | 0 |  | 3S6B | Q8IJP2 | 3 | 2 |
| 2XDV | Q8IUF8 | 1 | 1 |  | 2AMX | Q7RMV2 | 3 | 2 |
| 2XQ0 | Q10740 | 1 | 1 |  | 7WVB | P09467 | 3 | 0 |
| 2XSQ | Q96DE0 | 1 | 1 |  | 7QWJ | P32598 | 3 | 3 |
| 2XU9 | Q72HW2 | 1 | 1 |  | 7UX4 | B0KBL1 | 3 | 3 |
| 2XVY | Q72EC8 | 1 | 1 |  | 8PQ7 | M1C5B8 | 3 | 3 |
| 2XWH | P26660 | 1 | 0 |  | 4XKM | Q8A9M2 | 3 | 3 |
| 2XXZ | O15054 | 1 | 1 |  | 2OGI | Q8DY32 | 3 | 3 |
| 2XKJ | B0V9T6 | 1 | 0 |  | 3NR1 | Q8N4P3 | 3 | 3 |
| 2ZTG | O28029 | 1 | 1 |  | 2IBN | Q9UGB7 | 3 | 3 |
| 2ZTJ | O87198 | 1 | 1 |  | 2O08 | Q9KD90 | 3 | 3 |
| 2ZYL | P71875 | 1 | 1 |  | 7OIW | Q931Q4 | 3 | 3 |
| 3BSO | Q70ET3 | 1 | 1 |  | 7QM2 | P62136 | 3 | 0 |
| 3BWY | P21964 | 1 | 1 |  | 2NXF | Q7T291 | 3 | 3 |
| 3C3Y | Q6YI95 | 1 | 1 |  | 7QUY | O87871 | 3 | 3 |
| 3C4B | Q8R418 | 1 | 0 |  | 6N98 | A0A1K2FZ20 | 3 | 3 |
| 3C4Z | P28327 | 1 | 1 |  | 1G5B | P03772 | 3 | 3 |
| 3CBG | Q55813 | 1 | 1 |  | 1P8Q | P07824 | 3 | 3 |
| 3BON | P0DPI1 | 1 | 1 |  | 2P6B | Q08209 | 3 | 3 |
| 3CCG | Q97JL1 | 1 | 1 |  | 7TU8 | A3XHN1 | 3 | 3 |
| 3CLH | P56081 | 1 | 0 |  | 1FJM | P62139 | 3 | 3 |
| 3CP2 | P0A6U3 | 1 | 0 |  | 7UUT | P77990 | 3 | 3 |
| 3CRA | P0AEY3 | 1 | 0 |  | 3NIQ | Q9I6K2 | 3 | 3 |
| 3CRV | Q4JC68 | 1 | 0 |  | 2PAU | P76491 | 3 | 3 |
| 3CSK | Q08225 | 1 | 1 |  | 6N99 | A0A1K2FKX8 | 3 | 3 |
| 3D0C | Q8EMJ7 | 1 | 0 |  | 4PNC | C3TPN7 | 3 | 3 |
| 3CE2 | Q5L5N2 | 1 | 1 |  | 1YCG | Q9FDN7 | 3 | 2 |
| 3BOE | Q50EL4 | 1 | 1 |  | 2HBV | Q83V25 | 3 | 3 |
| 3BJ7 | P48026 | 1 | 0 |  | 1JGC | Q59738 | 3 | 0 |
| 3BHY | O43293 | 1 | 0 |  | 4LNI | P12425 | 3 | 2 |
| 2ZYQ | P9WNW7 | 1 | 1 |  | 7XY9 | P14941 | 3 | 3 |
| 2ZZF | O58035 | 1 | 1 |  | 6KI3 | P0C9C6 | 3 | 3 |
| 3A14 | Q9WZZ1 | 1 | 1 |  | 6VSS | G7JFU5 | 3 | 3 |
| 3A32 | Q9YDW0 | 1 | 1 |  | 6UFI | O34714 | 3 | 3 |
| 3A4J | Q93LD7 | 1 | 1 |  | 5ZWK | Q2TU34 | 3 | 3 |
| 3A99 | P11309 | 1 | 1 |  | 2HUO | Q9QXN5 | 3 | 3 |
| 3AAM | Q5SK18 | 1 | 1 |  | 7NS5 | P09201 | 3 | 3 |
| 3ALY | F9VN79 | 1 | 0 |  | 5B25 | Q01064 | 3 | 3 |
| 3AW5 | Q8ZWA8 | 1 | 1 |  | 1OLP | Q8GCY3 | 3 | 3 |
| 3B1B | P20507 | 1 | 1 |  | 1I74 | O68579 | 3 | 3 |
| 3B8B | Q8AAQ3 | 1 | 0 |  | 2EB0 | Q58025 | 3 | 3 |
| 3BAL | Q8GNT2 | 1 | 1 |  | 4V0W | P63087 | 3 | 3 |
| 3BC1 | Q9ERI2 | 1 | 1 |  | 1BXB | P26997 | 3 | 0 |
| 3BER | Q9H0S4 | 1 | 0 |  | 2YXO | Q5SLG2 | 3 | 3 |
| 3BHD | Q9BU02 | 1 | 0 |  | 2OHJ | Q50497 | 3 | 2 |
| 2WJE | Q9AHD4 | 1 | 1 |  | 6DKT | P39138 | 3 | 3 |
| 3D1M | Q62226 | 1 | 1 |  | 6KHY | P0C9C6 | 3 | 3 |
| 2WFP | P25081 | 1 | 0 |  | 7OBE | Q84XU2 | 3 | 3 |
| 2WEF | O95861 | 1 | 1 |  | 3CTZ | Q9NQW7 | 3 | 2 |
| 2PA7 | Q6T1W8 | 1 | 0 |  | 4GC3 | Q02150 | 3 | 3 |
| 2PCU | Q9UI42 | 1 | 1 |  | 4HJW | E9F0X0 | 3 | 3 |
| 2PGE | Q6ARP5 | 1 | 0 |  | 1XGS | P56218 | 3 | 3 |
| 2PLA | Q8N335 | 1 | 0 |  | 2WDD | Q72IT0 | 3 | 3 |
| 2PMQ | Q0FPQ4 | 1 | 1 |  | 3DYN | O76083 | 3 | 3 |
| 2PTZ | Q38BV6 | 1 | 1 |  | 5B8I | J3K8M7 | 3 | 3 |
| 2P8E | O75688 | 1 | 1 |  | 3ECM | O60658 | 3 | 3 |
| 2PU1 | Q9NDH8 | 1 | 1 |  | 1QTW | P0A6C1 | 3 | 3 |
| 2PW6 | P24197 | 1 | 1 |  | 4LE6 | Q5W503 | 3 | 3 |
| 2PZ0 | Q8RB32 | 1 | 1 |  | 4F0Z | Q08209 | 3 | 0 |
| 2Q09 | A0KF84 | 1 | 1 |  | 2VHL | O34450 | 3 | 3 |
| 2Q0D | Q381M1 | 1 | 1 |  | 3KNS | P04190 | 3 | 3 |
| 2Q18 | Q97UA0 | 1 | 0 |  | 3FPL | P14941 | 3 | 3 |
| 2Q3F | Q9NQL2 | 1 | 1 |  | 4JUQ | Q9HXY1 | 3 | 3 |
| 2PUZ | Q8U8Z6 | 1 | 1 |  | 1Y2K | Q08499 | 3 | 2 |
| 2Q4A | Q9LIG0 | 1 | 1 |  | 2WM1 | Q8TDX5 | 3 | 3 |
| 2P51 | O74856 | 1 | 1 |  | 1S70 | P62207 | 3 | 3 |
| 2OY2 | P22894 | 1 | 1 |  | 4HE2 | O00757 | 3 | 3 |
| 2NSM | P15169 | 1 | 0 |  | 4HNO | Q9WYJ7 | 3 | 3 |
| 2NX8 | Q5XE14 | 1 | 1 |  | 6FE3 | Q8WQX9 | 3 | 3 |
| 2NYT | Q9Y235 | 1 | 1 |  | 2Z1A | Q5SIP1 | 3 | 3 |
| 2O1C | P0AFC0 | 1 | 0 |  | 2Z72 | Q9S427 | 3 | 3 |
| 2O36 | P52888 | 1 | 1 |  | 6EZF | O00408 | 3 | 3 |
| 2O3E | P42676 | 1 | 1 |  | 2ZO4 | Q5SIE5 | 3 | 3 |
| 2OZT | Q8DJP8 | 1 | 0 |  | 6FV3 | A0QU89 | 3 | 3 |
| 2OB3 | P0A434 | 1 | 1 |  | 4HK7 | G3J531 | 3 | 3 |
| 2OFO | Q5QJ16 | 1 | 0 |  | 5ZT0 | P62137 | 3 | 3 |
| 2OG5 | Q9Z4Z5 | 1 | 0 |  | 5INB | P36873 | 3 | 0 |
| 2OKT | A0A0H2WWB5 | 1 | 0 |  | 5D6E | P50579 | 3 | 3 |
| 2OLA | A0A0H3KH80 | 1 | 0 |  | 3D03 | Q6XBH1 | 3 | 3 |
| 2OPW | Q5SRE7 | 1 | 0 |  | 3AAL | Q5KX27 | 3 | 3 |
| 2OXC | Q9UHI6 | 1 | 0 |  | 2Y1H | Q17R31 | 3 | 3 |
| 2ODN | Q7X416 | 1 | 0 |  | 2O8A | P63088 | 3 | 0 |
| 2Q4Z | Q9R1T5 | 1 | 1 |  | 5JPF | C4YM68 | 3 | 3 |
| 2Q73 | Q2F9Z1 | 1 | 1 |  | 1TCO | P48452 | 3 | 3 |
| 2QJT | Q5NHR1 | 1 | 1 |  | 1TBF | O76074 | 3 | 3 |
| 2V8T | Q72GH6 | 1 | 1 |  | 6DNO | P62136 | 3 | 0 |
| 2V8U | Q84DB4 | 1 | 1 |  | 5KAR | P58242 | 3 | 3 |
| 2VK9 | Q46149 | 1 | 0 |  | 2WXU | Q0TV31 | 3 | 3 |
| 2VO9 | Q37979 | 1 | 1 |  | 2WY6 | Q0TV31 | 3 | 3 |
| 2VQD | P37798 | 1 | 1 |  | 1Y44 | P54548 | 3 | 3 |
| 2VQM | P56524 | 1 | 1 |  | 2GQ1 | P0A993 | 3 | 0 |
| 2V7O | Q13555 | 1 | 0 |  | 1WOH | Q9RZ04 | 3 | 0 |
| 2VQR | Q1M964 | 1 | 1 |  | 1NUY | P00636 | 3 | 3 |
| 2VR2 | Q14117 | 1 | 1 |  | 3ICF | P53043 | 3 | 3 |
| 2W15 | P83512 | 1 | 1 |  | 2QYK | P27815 | 3 | 2 |
| 2W2X | P15153 | 1 | 1 |  | 5CET | P71615 | 3 | 3 |
| 2W3Z | Q8DV82 | 1 | 1 |  | 7K13 | Q83V25 | 3 | 3 |
| 2WAS | P19097 | 1 | 0 |  | 5CI1 | A0A0E1LZC3 | 3 | 1 |
| 2WBQ | Q6WZB0 | 1 | 0 |  | 5VJW | Q9SR62 | 3 | 3 |
| 2VQX | Q5MJ80 | 1 | 0 |  | 1YIX | P0AFQ7 | 3 | 2 |
| 2V4U | Q9NRF8 | 1 | 0 |  | 1K7H | Q9BHT8 | 3 | 3 |
| 2V4B | Q9UHI8 | 1 | 1 |  | 1O12 | Q9WZS1 | 3 | 1 |
| 2V27 | Q47XN7 | 1 | 1 |  | 2QJC | Q57U41 | 3 | 3 |
| 2QKR | Q5CRJ8 | 1 | 0 |  | 6BOJ | Q07343 | 3 | 3 |
| 2QLT | P41277 | 1 | 1 |  | 1M35 | P15034 | 3 | 3 |
| 2QME | P60763 | 1 | 1 |  | 4OFC | Q8TDX5 | 3 | 3 |
| 2QN0 | P18640 | 1 | 1 |  | 7LOL | P60651 | 3 | 3 |
| 2QQ0 | Q9WYN2 | 1 | 1 |  | 2QYM | Q08493 | 3 | 2 |
| 2QTY | Q8CG72 | 1 | 1 |  | 1ZZM | P39408 | 3 | 3 |
| 2QXF | P04995 | 1 | 1 |  | 1Y9A | P35630 | 3 | 3 |
| 2QZ4 | Q9UQ90 | 1 | 0 |  | 5ZHZ | P9WQ13 | 3 | 3 |
| 2R2L | Q04631 | 1 | 0 |  | 5FIB | Q04519 | 3 | 3 |
| 2R5V | O52791 | 1 | 1 |  | 6XIA | P24299 | 3 | 0 |
| 2R8R | Q883V3 | 1 | 0 |  | 3WFS | O67911 | 4 | 0 |
| 2R9V | Q9X1U7 | 1 | 0 |  | 5FC1 | P70158 | 4 | 4 |
| 2RDQ | Q82IZ1 | 1 | 1 |  | 7WNT | P9WGZ9 | 4 | 4 |
| 2UVL | Q13489 | 1 | 1 |  | 1A0E | P45687 | 4 | 4 |
| 2V06 | A0QTQ6 | 1 | 1 |  | 8PPV | Q9V2F3 | 4 | 4 |
| 2WEL | Q13557 | 1 | 0 |  | 6RZ0 | P0AC84 | 4 | 4 |
| 2NSF | Q8NLC1 | 1 | 1 |  | 2GSO | Q8PIS1 | 4 | 4 |
| 3D2Y | P75820 | 1 | 0 |  | 5A0T | O86842 | 4 | 4 |
| 3DG6 | A0QTN8 | 1 | 1 |  | 4MLM | D0E8I5 | 4 | 4 |
| 3N9S | D0IR47 | 1 | 1 |  | 3LHL | Q18A84 | 4 | 4 |
| 3N9T | C6FI44 | 1 | 1 |  | 2QED | Q8ZRM2 | 4 | 4 |
| 3NAS | O06995 | 1 | 0 |  | 1A0C | P19148 | 4 | 4 |
| 3NF3 | Q7B8V4 | 1 | 1 |  | 4XIM | P12851 | 4 | 4 |
| 3NH4 | Q91XE4 | 1 | 1 |  | 2EF4 | Q5SI78 | 4 | 0 |
| 3NKV | Q9H0U4 | 1 | 1 |  | 1EI6 | Q51782 | 4 | 4 |
| 3N9N | Q9GYI0 | 1 | 1 |  | 5VEM | Q9UJA9 | 4 | 4 |
| 3NUR | A0A0H3K164 | 1 | 1 |  | 3AJ3 | Q988B9 | 4 | 4 |
| 3NZK | A1JJJ9 | 1 | 1 |  | 2EIV | Q5SI78 | 4 | 0 |
| 3O2R | Q9PM40 | 1 | 0 |  | 3JYF | A6THC4 | 4 | 4 |
| 3OAJ | P96693 | 1 | 1 |  | 6K6S | Q8CT16 | 4 | 4 |
| 3OCQ | Q57LE3 | 1 | 1 |  | 1MUW | P15587 | 4 | 4 |
| 3OF5 | Q5NGB5 | 1 | 0 |  | 4V0H | A4D2B0 | 4 | 4 |
| 3OGA | Q8ZNF5 | 1 | 0 |  | 7A1F | Q9H816 | 4 | 4 |
| 3NW4 | Q67FT0 | 1 | 1 |  | 5B4B | Q9I2V0 | 4 | 0 |
| 3OJ6 | P0CI02 | 1 | 1 |  | 5I81 | P17405 | 4 | 4 |
| 3N5F | Q53389 | 1 | 0 |  | 6INT | A0A4V8H014 | 4 | 4 |
| 3MXW | Q15465 | 1 | 1 |  | 1DXI | P37031 | 4 | 4 |
| 3LQB | Q1LW01 | 1 | 1 |  | 5Q22 | Q6PJP8 | 4 | 3 |
| 3LV0 | A0A0H3M6W8 | 1 | 0 |  | 4HHL | Q9ZAI3 | 4 | 4 |
| 3LW6 | Q9VBZ9 | 1 | 1 |  | 2P18 | Q2PYN0 | 4 | 4 |
| 3M2W | P49137 | 1 | 0 |  | 3ZQ4 | Q45493 | 4 | 4 |
| 3M4R | Q9HKW2 | 1 | 1 |  | 4AD9 | Q53H82 | 4 | 4 |
| 3MDU | Q9HU77 | 1 | 1 |  | 4HHM | Q9ZAI3 | 4 | 4 |
| 3N1G | O43323 | 1 | 1 |  | 1A0D | P54273 | 4 | 4 |
| 3MF2 | Q89VT8 | 1 | 1 |  | 5TCD | Q6UWV6 | 4 | 4 |
| 3MJM | B1IV40 | 1 | 1 |  | 1QH5 | Q16775 | 4 | 4 |
| 3MK1 | P05187 | 1 | 1 |  | 1QT1 | P50910 | 4 | 4 |
| 3MND | Q8WRF5 | 1 | 1 |  | 6VRS | P24300 | 4 | 4 |
| 3MPZ | A0QT11 | 1 | 1 |  | 4LR2 | Q9Y6X5 | 4 | 4 |
| 3MSR | Q4A724 | 1 | 0 |  | 3BK2 | Q72JJ7 | 4 | 4 |
| 3MWC | C5CFI0 | 1 | 1 |  | 1XM8 | Q9SID3 | 4 | 4 |
| 3MFI | Q04049 | 1 | 1 |  | 4KP6 | Q07343 | 4 | 3 |
| 3OKF | Q9KNV2 | 1 | 0 |  | 1XFK | Q9KSQ2 | 4 | 0 |
| 3OPT | P39956 | 1 | 1 |  | 3GVE | O34313 | 4 | 4 |
| 3OSN | Q9UNA4 | 1 | 1 |  | 2DFJ | Q83SQ2 | 4 | 4 |
| 3R2N | A0A0H3MRL9 | 1 | 1 |  | 4XIA | P12070 | 4 | 2 |
| 3R3H | Q5ZT85 | 1 | 0 |  | 8E5N | P05089 | 4 | 4 |
| 3R9X | O67800 | 1 | 1 |  | 5VEN | Q9EQG7 | 4 | 4 |
| 3RAP | P10114 | 1 | 1 |  | 3SZY | Q92UV8 | 4 | 4 |
| 3REG | P31021 | 1 | 1 |  | 1BXC | P56681 | 4 | 0 |
| 3RL5 | B1WBP0 | 1 | 0 |  | 5EQV | A0A5P8YBY3 | 4 | 4 |
| 3R1J | A0A0H2ZSG9 | 1 | 1 |  | 4G3H | O25949 | 4 | 4 |
| 3RLG | P0CE80 | 1 | 1 |  | 8WMY | A0A4U7IGH6 | 4 | 4 |
| 3RV4 | P24182 | 1 | 1 |  | 1P9E | Q841S6 | 5 | 5 |
| 3RZV | O95630 | 1 | 1 |  | 3IE0 | Q5SLP1 | 5 | 5 |
| 3S4L | Q57829 | 1 | 1 |  | 3IB7 | P9WP65 | 5 | 5 |
| 3S57 | Q6NS38 | 1 | 1 |  | 3ZWF | Q9H777 | 5 | 5 |
| 3S5S | A9GEI3 | 1 | 0 |  | 2R2D | A9CKY2 | 5 | 5 |
| 3S8P | Q4FZB7 | 1 | 0 |  | 2CBN | P0A8V0 | 5 | 5 |
| 3RQ4 | Q86Y97 | 1 | 0 |  | 8EWO | Q9I2T1 | 5 | 5 |
| 3QY1 | Q8ZRS0 | 1 | 1 |  | 2ZO9 | Q6XBH1 | 5 | 5 |
| 3QXC | O24872 | 1 | 1 |  | 8W6P | P70158 | 5 | 5 |
| 3QDF | B2HIH3 | 1 | 1 |  | 5I8R | P17405 | 5 | 5 |
| 3OTD | Q9NWX6 | 1 | 0 |  | 6UNC | I6YEE1 | 5 | 5 |
| 3OVA | O28126 | 1 | 0 |  | 8T1Q | Q9UKF6 | 5 | 5 |
| 3P5J | Q9CWY8 | 1 | 0 |  | 5EHT | A3FJ64 | 5 | 5 |
| 3P5P | Q41594 | 1 | 1 |  | 3DHA | P0CJ63 | 5 | 5 |
| 3PM6 | P0CJ44 | 1 | 1 |  | 6I1D | Q06224 | 5 | 5 |
| 3PN3 | Q9FUZ2 | 1 | 1 |  | 6M8Q | Q9UKF6 | 6 | 6 |
| 3PNU | Q0PBP6 | 1 | 1 |  |  |  |  |  |

**Table S4**. The details of previously neglected mono-metal-binding sites identified by MeSiteIG.

| **PDB** | **UniProt**  **ID** | **Predicted Triplet^a^** | **Most likely metal ion^b^** | **Density^c^** | **PDB** | **UniProt**  **ID** | **Predicted Triplet** | **Most likely metal ion** | **Density^a^** |
| --- | --- | --- | --- | --- | --- | --- | --- | --- | --- |
| 1BG7 | P07229 | GLU23-GLU58-HIS61 | ['FE'] | No | 6C45 | Q15181 | ASP116-ASP121-ASP153 | ['MG'] | Yes |
| 1BQG | P42206 | ASP241-GLU266-ASN295 | ['MG'] | Yes | 8CR2 | O43681 | ASP304-GLU307-ASP308 | ['MG'] | No |
| 1BYI | P13000 | GLU115-THR16-ASP54 | ['MG'] | Yes | 5XVB | A0A3B6UEP7 | CYS546-CYS549-CYS61 | ['NI'] | No |
| 1DCS | P18548 | HIS183-ASP185-HIS243 | ['ZN'] | Yes | 5XVB | A0A3B6UEP7 | CYS549-CYS61-CYS64 | ['NI'] | No |
| 1DI1 | Q03471 | ASN244-SER248-GLU252 | ['MG'] | No | 6FAD | Q96SB4 | GLU217-ASN218-ASP497 | ['MG'] | No |
| 1E5R | O09345 | HIS107-ASP109-HIS158 | ['ZN'] | Yes | 4F7K | E1ACR6 | HIS376-HIS378-HIS421 | ['ZN'] | Yes |
| 1EKE | Q57599 | ASP112-ASP7-GLU8 | ['MG'] | Yes | 5C0Y | Q12149 | ASP238-GLU240-ASP365 | ['MG'] | Yes |
| 1FTH | P0A2W6 | ASP1010-GLU1012-GLU1060 | ['MG'] | Yes | 2C00 | P37798 | GLU276-GLU288-ASN290 | ['CA'] | No |
| 1G71 | Q9P9H1 | HIS151-ASP95-ASP97 | ['CD'] | Yes | 1GTI | P19157 | ASP94-GLU97-ASP98 | ['MG'] | No |
| 1G71 | Q9P9H1 | ASP280-ASP95-ASP97 | ['MN'] | Yes | 1OJ7 | Q46856 | ASP194-HIS267-HIS281 | ['ZN'] | No |
| 1I39 | O29634 | ASP101-ASP6-GLU7 | ['MG'] | Yes | 7E15 | Q5JGL0 | HIS106-ASP36-ASP83 | ['MN'] | Yes |
| 1I88 | P30074 | ASP255-HIS257-HIS266 | ['ZN'] | No | 7E15 | Q5JGL0 | ASP166-ASP36-ASP83 | ['MN'] | Yes |
| 1IK6 | Q8ZUR7 | ASP244-ASP246-THR247 | ['MG'] | Yes | 2OTD | A0A0H2V2B5 | GLU114-GLU39-ASP41 | ['MG'] | No |
| 1IM4 | P96022 | ASP105-GLU106-ASP7 | ['MG'] | No | 1J5S | Q9WXR9 | HIS30-HIS32-ASP397 | ['ZN'] | Yes |
| 1J6O | Q9WZD5 | HIS126-HIS151-GLU90 | ['ZN'] | Yes | 3IEC | Q7KZI7 | GLU179-ASN180-ASP193 | ['MG'] | No |
| 1J6O | Q9WZD5 | ASP201-HIS4-HIS6 | ['ZN'] | Yes | 6O64 | O48661 | ASP247-GLU250-ASP251 | ['MG'] | No |
| 1JC4 | Q8VQN0 | HIS12-GLU141-HIS91 | ['ZN'] | Yes | 7A03 | A0A822ZZF9 | HIS267-HIS271-GLU297 | ['ZN'] | Yes |
| 1JGC | Q59738 | GLU127-GLU18-GLU51 | ['FE'] | No | 1WMW | Q5SMD0 | ASP79-GLU82-ASP83 | ['MG'] | Yes |
| 1JGC | Q59738 | GLU127-GLU51-GLU94 | ['FE'] | No | 2AMJ | P0AEY7 | ASP94-ASP95-THR98 | ['MG'] | No |
| 1JGC | Q59738 | GLU18-GLU51-HIS54 | ['FE'] | No | 6ZS1 | G0S1F8 | HIS121-HIS47-HIS49 | ['ZN'] | Yes |
| 1JLN | Q62132 | ASP462-GLU465-ASP466 | ['MG'] | No | 4AEE | A3DM60 | ASP272-GLU275-ASP276 | ['MG'] | No |
| 1L5X | Q8ZU79 | ASP8-ASN93-ASP9 | ['MG'] | Yes | 1X8G | P26918 | ASN116-HIS118-HIS196 | ['ZN'] | No |
| 1LL3 | P13280 | ASP101-ASP103-HIS211 | ['MN'] | Yes | 7PJC | A0A1D8PSA9 | SER117-ASP285-ASP287 | ['CD'] | No |
| 1LME | P96113 | GLU130-HIS133-GLN49 | ['CD'] | Yes | 7PJC | A0A1D8PSA9 | ASP285-ASP287-ASP289 | ['MG'] | No |
| 1NJG | P06710 | ASP126-GLU92-ASP94 | ['MG'] | No | 7Q74 | Q10295 | ASP108-ASP110-SER97 | ['MG'] | No |
| 1NPC | P05806 | TYR158-GLU167-HIS232 | ['ZN'] | No | 7QX8 | Q84WV0 | ASP297-GLU300-ASP301 | ['MG'] | No |
| 1O0X | Q9X1I7 | ASP100-ASP111-GLU238 | ['MG'] | Yes | 7UM1 | A0A172JIC8 | ASP370-ASP372-ASP374 | ['MG'] | No |
| 1O0X | Q9X1I7 | ASP111-HIS174-GLU238 | ['MN'] | Yes | 7TQQ | Q9NSU2 | ASP18-ASP200-GLU20 | ['MG'] | Yes |
| 1O12 | Q9WZS1 | GLU115-HIS49-HIS51 | ['ZN'] | Yes | 7U6O | A0A090M4C4 | GLU229-HIS358-GLU489 | ['MN'] | No |
| 1O12 | Q9WZS1 | ASP255-HIS49-HIS51 | ['ZN'] | Yes | 7U6O | A0A090M4C4 | GLU231-GLU302-GLU309 | ['MG'] | No |
| 1O1Z | Q9X1V6 | GLU33-ASP35-GLU97 | ['MG'] | Yes | 8DG4 | P69949 | ASP243-GLU292-ASP319 | ['MG'] | No |
| 1OGL | O15923 | GLU49-GLU77-ASP80 | ['MG'] | No | 7V4H | A0A0R0EVM7 | GLU129-HIS249-GLU330 | ['MN'] | No |
| 1P6O | Q12178 | ASP151-GLU154-ASP155 | ['MG'] | No | 7V4H | A0A0R0EVM7 | GLU131-GLU192-GLU199 | ['MG'] | No |
| 1PJR | P56255 | ASP223-GLU224-LYS37 | ['MG'] | No | 7XJG | P23070 | ASP119-ASP197-ASP198 | ['MG'] | No |
| 1Q0H | P45568 | ASP150-GLU152-GLU231 | ['MG'] | No | 7WIK | A0R1E6 | GLU11-ASP162-ASP9 | ['MG'] | No |
| 1R8G | P77213 | HIS136-GLU17-GLU237 | ['MN'] | Yes | 7XTJ | A0A023J5W7 | ASP191-ASP193-SER194 | ['MG'] | No |
| 1RV9 | Q9K0A8 | CYS118-HIS135-HIS82 | ['ZN'] | Yes | 8A6W | P9WPL5 | ASP157-ASP158-THR161 | ['MG'] | Yes |
| 1RW0 | Q8Z4J1 | CYS107-HIS124-HIS71 | ['ZN'] | No | 8AC5 | Q6CGY9 | GLU154-HIS74-HIS78 | ['ZN'] | No |
| 1T5J | Q58588 | ASP253-ASP255-SER256 | ['MG'] | No | 8B3C | Q96562 | ASP313-GLU316-ASP317 | ['MG'] | Yes |
| 1TZP | P0C0T5 | HIS113-ASP120-HIS211 | ['ZN'] | Yes | 8B4M | D5SK09 | ASN225-SER229-GLU233 | ['MG'] | No |
| 1U4B | P52026 | ASP653-ASP830-GLU831 | ['MG'] | Yes | 8BCW | A0A3G1AUL2 | ASP55-ASP57-SER58 | ['MG'] | No |
| 1U8X | P54716 | ASP257-ASP259-THR260 | ['MG'] | Yes | 8DI0 | G8UJW8 | ASP326-HIS327-ASP40 | ['ZN'] | No |
| 1UJN | P83703 | GLU173-HIS231-HIS247 | ['ZN'] | Yes | 8EYO | Q16798 | GLU280-ASP281-ASP304 | ['MG'] | No |
| 1ULZ | O67483 | GLU274-GLU287-ASN289 | ['CA'] | No | 8IFZ | A0A6J0Z472 | HIS374-HIS378-GLU402 | ['ZN'] | No |
| 1V33 | O57934 | HIS151-ASP95-ASP97 | ['CD'] | Yes | 8HZ4 | A9W9X0 | GLU275-GLU287-ASN289 | ['CA'] | No |
| 1V33 | O57934 | ASP280-ASP95-ASP97 | ['MN'] | Yes | 8HZ5 | A9WKH8 | GLU275-GLU288-ASN290 | ['CA'] | No |
| 1V3Y | P43522 | GLU146-HIS149-GLN49 | ['CD'] | Yes | 8SSG | D3DIV8 | ASP140-GLU143-ASP144 | ['MG'] | No |
| 1V4A | P30870 | ASP173-ASP175-ASP223 | ['MG'] | Yes | 8UOY | A3F8V6 | ASP243-GLU292-ASP319 | ['MG'] | No |
| 1VD6 | Q53W25 | GLU100-GLU40-ASP42 | ['MG'] | Yes | 6DLI | Q5SHT7 | ASP85-GLU88-ASP89 | ['MG'] | Yes |
| 1VHE | P94521 | ASP182-GLU215-HIS325 | ['ZN'] | Yes | 5WRU | O77392 | ASP198-ASP203-ASP235 | ['MG'] | No |
| 1VK6 | P32664 | GLU174-GLU178-GLU219 | ['MG'] | No | 3NR9 | P49760 | GLU294-ASN295-ASP327 | ['MG'] | No |
| 1W55 | Q9PM68 | ASP217-HIS219-HIS251 | ['ZN'] | Yes | 6MJP | O30650 | ASP116-GLU119-ASP120 | ['MG'] | Yes |
| 1W6K | P48449 | ASP326-ASP327-THR330 | ['MG'] | Yes | 4FN6 | Q6GEY1 | ASP17-GLU20-ASP21 | ['MG'] | Yes |
| 1W9Y | Q08506 | HIS177-ASP179-HIS234 | ['ZN'] | No | 4C5Z | G3XP38 | HIS111-HIS113-ASP378 | ['ZN'] | Yes |
| 1XFJ | Q9AAV3 | CYS114-HIS131-HIS77 | ['ZN'] | Yes | 4DKI | A0A0H2WXF8 | ASP35-GLU38-ASP39 | ['MG'] | No |
| 1XFK | Q9KSQ2 | HIS127-ASP157-ASP161 | ['MN'] | No | 4W4U | N1P0J5 | ASP119-ASP120-THR123 | ['MG'] | No |
| 1XFK | Q9KSQ2 | HIS127-ASP157-ASP254 | ['MN'] | No | 4XH9 | Q7Z628 | ASP188-GLU191-ASP192 | ['MG'] | Yes |
| 1XFK | Q9KSQ2 | ASP157-HIS159-ASP256 | ['ZN'] | No | 5DM3 | Q1QZR8 | GLU143-HIS262-GLU350 | ['MN'] | No |
| 1XFK | Q9KSQ2 | ASP157-ASP161-ASP254 | ['MN'] | No | 5DM3 | Q1QZR8 | GLU145-GLU206-GLU213 | ['MG'] | No |
| 1XI6 | Q8TZH9 | ASP202-ASP84-ASP87 | ['MG'] | No | 2O8A | P63088 | ASN124-HIS173-ASP92 | ['MN'] | Yes |
| 1XMB | P54970 | CYS137-HIS139-HIS197 | ['ZN'] | Yes | 2O8A | P63088 | HIS173-HIS248-ASP92 | ['ZN'] | Yes |
| 1XR5 | P03303 | ASP233-ASP327-ASP328 | ['MG'] | Yes | 2O8A | P63088 | ASP64-HIS66-ASP92 | ['ZN'] | Yes |
| 1XWY | P27859 | HIS127-HIS152-GLU91 | ['ZN'] | Yes | 6G80 | K5B7F3 | ASP141-HIS169-ASP170 | ['MG'] | No |
| 1Y2K | Q08499 | ASP397-GLU400-ASP401 | ['MG'] | No | 6DKH | P39346 | CYS40-HIS65-GLU66 | ['ZN'] | No |
| 1Y97 | Q9BQ50 | ASP14-GLU16-ASP193 | ['MG'] | No | 6DKH | P39346 | GLU153-HIS65-GLU66 | ['MN'] | No |
| 1YIX | P0AFQ7 | HIS155-CYS156-GLU203 | ['ZN'] | No | 2HK0 | A9CH28 | GLU150-ASP183-HIS209 | ['MN'] | Yes |
| 1YNB | O28840 | ASP124-HIS42-ASP75 | ['MN'] | Yes | 7E2P | A0A7D5V839 | ASP250-GLU312-ASP339 | ['MG'] | No |
| 1YNB | O28840 | ASP124-HIS74-ASP75 | ['ZN'] | Yes | 7E2P | A0A7D5V839 | ASP250-ASP313-ASP339 | ['MG'] | No |
| 1YVF | O93077 | ASP125-GLU128-ASP129 | ['MG'] | No | 3HIY | C7AJA4 | ASP130-ASP65-ASP67 | ['MG'] | Yes |
| 1YVW | Q81G00 | GLU43-GLU62-ASP65 | ['MG'] | No | 3IAC | Q8ZM23 | HIS33-HIS35-ASP414 | ['ZN'] | Yes |
| 1Z1E | Q9SLV5 | ASP92-GLU95-ASP96 | ['MG'] | No | 3JVI | C4LSE7 | ASP137-GLU140-ASP141 | ['MG'] | Yes |
| 1Z9T | P33644 | CYS107-HIS124-HIS71 | ['ZN'] | No | 7KWS | Q0P8H3 | ASP86-ASP88-ASP95 | ['MN'] | No |
| 1ZX5 | O30200 | HIS100-HIS175-HIS98 | ['CU'] | No | 4JN6 | P9WMK5 | ASP260-GLU263-ASP264 | ['MG'] | No |
| 2A8B | Q15256 | ASP570-GLU573-ASP574 | ['MG'] | No | 7NWC | P54687 | ASP314-ASP315-THR318 | ['MG'] | No |
| 2AFW | Q16769 | HIS140-ASP159-ASP248 | ['ZN'] | No | 1U0W | P30074 | ASP255-HIS257-HIS266 | ['ZN'] | Yes |
| 2AMX | Q7RMV2 | ASP116-GLU119-ASP120 | ['MG'] | Yes | 4IL0 | Q46915 | ASP234-GLU259-ASN288 | ['MG'] | Yes |
| 2B4V | Q86MV5 | ASP267-ASP97-ASP99 | ['MG'] | No | 2I5Q | P77215 | ASP226-GLU252-GLU280 | ['MG'] | Yes |
| 2BMX | P9WQB7 | ASP73-GLU76-ASP77 | ['MG'] | No | 4GU7 | Q9FBY9 | ASP307-GLU310-ASP311 | ['MG'] | No |
| 2C07 | Q8I2S7 | ASP162-GLU165-ASP166 | ['MG'] | No | 4CJN | A0A0J9X1X5 | ASP35-GLU38-ASP39 | ['MG'] | No |
| 2CGL | P32171 | ASP52-ASP54-SER55 | ['MG'] | Yes | 3TZQ | B2HKN3 | ASP108-ASP109-THR112 | ['MG'] | Yes |
| 2CMW | Q9HCP0 | GLU168-ASN169-ASP187 | ['MG'] | Yes | 6NIC | G7JT50 | ASP218-ASP220-THR221 | ['MG'] | No |
| 2CWL | Q5SM21 | GLU36-GLU70-HIS73 | ['MN3'] | Yes | 6J36 | A0A223MA21 | ASP250-GLU307-ASP334 | ['MG'] | Yes |
| 2D1F | P9WG59 | ASP332-ASP334-THR335 | ['MG'] | No | 6J36 | A0A223MA21 | ASP250-ASP308-ASP334 | ['MG'] | Yes |
| 2D5R | Q9UIV1 | ASP230-ASP40-GLU42 | ['MG'] | No | 5N1Q | A0A247D6X3 | ASP371-GLU374-ASP375 | ['MG'] | Yes |
| 2DDK | O14732 | ASP101-ASP104-ASP231 | ['MG'] | No | 5N5F | D0LZ73 | GLU31-GLU61-HIS64 | ['FE'] | No |
| 2DI4 | O67077 | HIS418-HIS422-ASP496 | ['ZN'] | No | 8G5X | Q5NEJ8 | GLU214-ASP84-GLU87 | ['MG'] | Yes |
| 2E0L | P07998 | ASP14-ASP16-SER17 | ['MG'] | Yes | 3OTR | B9PH47 | ASP252-GLU303-ASP330 | ['MG'] | Yes |
| 2E6M | O09053 | ASP210-ASP76-GLU78 | ['MG'] | Yes | 3OTR | B9PH47 | ASP252-ASP304-ASP330 | ['MG'] | Yes |
| 2E8V | Q12051 | ASP80-GLU83-ASP84 | ['MG'] | Yes | 5UAM | A0A1W2VMZ5 | HIS334-ASP336-HIS342 | ['MN'] | No |
| 2EER | Q96XE0 | CYS154-CYS38-HIS68 | ['ZN'] | No | 6U8J | A0A2H0ZWN3 | HIS283-ASP346-CYS70 | ['MN'] | No |
| 2EER | Q96XE0 | CYS38-HIS68-GLU69 | ['ZN'] | No | 6AE3 | Q9WV60 | GLN185-ASN186-ASP200 | ['MG'] | Yes |
| 2EF4 | Q5SI78 | ASP119-HIS121-ASP220 | ['ZN'] | Yes | 2PZI | P9WI73 | GLU280-ASN281-ASP293 | ['MG'] | No |
| 2EF4 | Q5SI78 | ASP119-ASP123-ASP218 | ['MN'] | Yes | 2PZI | P9WI73 | ASP551-ASP553-SER554 | ['MG'] | No |
| 2EF4 | Q5SI78 | ASP119-ASP123-HIS97 | ['MN'] | Yes | 3AMI | F2Z284 | GLU157-HIS77-HIS81 | ['ZN'] | Yes |
| 2EF4 | Q5SI78 | ASP119-ASP218-HIS97 | ['MN'] | Yes | 6VLC | A0A0U1RGY0 | ASP80-GLU83-ASP84 | ['MG'] | Yes |
| 2F7V | Q8P8J5 | ASP103-GLU131-HIS340 | ['ZN'] | Yes | 2Q01 | Q9A874 | HIS39-ASP416-HIS41 | ['ZN'] | Yes |
| 2F8H | A0A0H2X6W0 | ASP103-GLU131-HIS340 | ['ZN'] | Yes | 6CSJ | A0A150JSL8 | ASP171-HIS254-HIS271 | ['ZN'] | No |
| 2FFI | Q88M75 | HIS18-HIS20-ASP236 | ['ZN'] | No | 4D28 | Q9LDI3 | GLU138-ASN139-ASP152 | ['MG'] | No |
| 2G8L | O59272 | ASP156-ASN157-ASP191 | ['MG'] | Yes | 5GUE | C9K1X5 | ASP259-GLU262-ASP263 | ['MG'] | No |
| 2GDQ | O06741 | ASP190-GLU217-GLU243 | ['MG'] | Yes | 4D7K | K4RFM2 | ASP168-ASP169-THR172 | ['MG'] | No |
| 2GDQ | O06741 | GLU217-GLU243-HIS293 | ['MG'] | Yes | 1LL0 | P13280 | ASP101-ASP103-HIS211 | ['MN'] | No |
| 2GQ1 | P0A993 | ASP110-ASP113-GLU275 | ['MG'] | Yes | 2F7L | Q976E4 | ASP241-ASP243-ASP245 | ['MG'] | No |
| 2GQ1 | P0A993 | ASP110-ASP113-GLU89 | ['MG'] | Yes | 1BXB | P26997 | GLU180-ASP244-ASP286 | ['MG'] | No |
| 2GQ1 | P0A993 | ASP110-GLU275-GLU89 | ['MG'] | Yes | 1BXB | P26997 | GLU216-HIS219-ASP254 | ['MG'] | Yes |
| 2GSR | P80031 | ASP92-GLU95-ASP96 | ['MG'] | Yes | 1BXB | P26997 | GLU216-ASP254-ASP256 | ['MG'] | Yes |
| 2HAI | Q99AU2 | ASP125-GLU128-ASP129 | ['MG'] | No | 2UU7 | Q8HZM5 | GLU134-HIS253-GLU338 | ['MN'] | No |
| 2HHP | P29468 | ASP100-ASP102-SER89 | ['MG'] | Yes | 3PDK | Q81VN7 | ASP240-ASP242-ASP244 | ['MG'] | No |
| 2HHS | Q45458 | ASP653-ASP830-GLU831 | ['MG'] | Yes | 1VJ7 | Q54089 | ASP219-ASP220-THR223 | ['MG'] | No |
| 2HHV | Q5KWC1 | ASP653-ASP830-GLU831 | ['MG'] | Yes | 4OBX | P49017 | ASP257-ASP259-SER260 | ['MG'] | No |
| 2I5E | Q8PU52 | ASP168-ASP170-ASP94 | ['MG'] | Yes | 2DZD | Q05FZ3 | GLU280-GLU292-ASN294 | ['CA'] | No |
| 2I5I | P59745 | HIS129-ASP12-HIS63 | ['MG'] | Yes | 5ZHH | Q8YT10 | ASP212-ASP84-ASP87 | ['MG'] | Yes |
| 2I87 | Q5HEB7 | GLU220-ASP293-GLU306 | ['MG'] | No | 4A5Q | B6A876 | ASP355-ASP356-THR359 | ['MG'] | No |
| 2IVN | Q9UXT7 | HIS107-HIS111-ASP285 | ['ZN'] | No | 3GFB | Q5JI69 | CYS42-HIS67-GLU68 | ['ZN'] | No |
| 2J43 | Q8KLP1 | ASP102-HIS106-HIS108 | ['ZN'] | Yes | 3F9T | Q60358 | ASP164-GLU167-ASP168 | ['MG'] | No |
| 2JGN | O00571 | ASP454-GLU457-ASP458 | ['MG'] | No | 1YCG | Q9FDN7 | ASP167-HIS81-GLU83 | ['FE'] | No |
| 2KFN | P00582 | ASP705-ASP882-GLU883 | ['MG'] | No | 3FKY | P32288 | GLU131-HIS250-GLU331 | ['MN'] | No |
| 2NSM | P15169 | HIS196-HIS66-GLU69 | ['ZN'] | Yes | 3FKY | P32288 | GLU133-GLU193-GLU200 | ['MG'] | No |
| 2O1C | P0AFC0 | GLU117-GLU56-GLU60 | ['NI'] | Yes | 7E2Q | P75189 | ASP256-GLU310-ASP337 | ['MG'] | Yes |
| 2ODN | Q7X416 | ASP112-ASP114-SER115 | ['MG'] | No | 4YHJ | P32298 | GLU316-ASN317-ASP330 | ['MG'] | No |
| 2OFO | Q5QJ16 | ASP112-ASP114-SER115 | ['MG'] | Yes | 1VMG | Q97U11 | GLU35-GLU54-ASP57 | ['MG'] | Yes |
| 2OG5 | Q9Z4Z5 | HIS155-GLU157-HIS287 | ['FE'] | Yes | 3AL5 | A2RUC4 | HIS160-ASP162-HIS235 | ['ZN'] | No |
| 2OKT | A0A0H2WWB5 | ASP171-GLU196-ASP219 | ['MG'] | Yes | 2CXI | O73984 | ASP337-GLU340-ASP341 | ['MG'] | Yes |
| 2OLA | A0A0H3KH80 | ASP171-GLU196-ASP219 | ['MG'] | Yes | 4K2B | O07566 | ASP79-GLU82-ASP83 | ['MG'] | Yes |
| 2OPW | Q5SRE7 | HIS156-ASP158-HIS246 | ['ZN'] | Yes | 3GV5 | Q9UNA4 | ASP126-GLU127-ASP34 | ['MG'] | Yes |
| 2OXC | Q9UHI6 | LYS112-ASP211-GLU212 | ['MG'] | Yes | 6EEM | O82415 | ASP445-GLU448-ASP449 | ['MG'] | No |
| 2OZT | Q8DJP8 | ASP165-GLU194-ASP219 | ['MG'] | Yes | 4AGU | Q00532 | GLU130-ASN131-ASP144 | ['MG'] | No |
| 2PA7 | Q6T1W8 | HIS49-HIS51-HIS95 | ['CU'] | Yes | 2FJK | Q703I2 | HIS178-HIS208-HIS81 | ['ZN'] | No |
| 2PGE | Q6ARP5 | ASP214-GLU240-ASP265 | ['MG'] | Yes | 2P0U | Q5I6Y1 | ASP273-HIS275-HIS284 | ['ZN'] | No |
| 2PLA | Q8N335 | ASP195-ASP197-THR198 | ['MG'] | No | 2DFU | Q5SK43 | GLU114-GLU116-ASP145 | ['MG'] | Yes |
| 2Q18 | Q97UA0 | GLU143-GLU145-ASP164 | ['MG'] | Yes | 3ZLG | Q5XD01 | ASP243-GLU292-ASP319 | ['MG'] | No |
| 2Q2L | B2CP37 | HIS119-HIS45-HIS47 | ['ZN'] | Yes | 3ZLG | Q5XD01 | ASP243-ASP293-ASP319 | ['MG'] | No |
| 2QKR | Q5CRJ8 | GLN148-ASN149-ASP162 | ['MG'] | No | 1BKH | Q51958 | ASP198-GLU224-ASP249 | ['MG'] | Yes |
| 2QYK | P27815 | ASP609-GLU612-ASP613 | ['MG'] | Yes | 2FVZ | O14732 | ASP101-ASP104-ASP231 | ['MG'] | No |
| 2QYM | Q08493 | ASP519-GLU522-ASP523 | ['MG'] | Yes | 6ZHT | P52490 | ASP124-GLU127-ASP128 | ['MG'] | Yes |
| 2QZ4 | Q9UQ90 | LYS355-ASP408-GLU409 | ['MG'] | No | 1SMQ | P09938 | GLU239-GLU273-HIS276 | ['FE'] | No |
| 2R2L | Q04631 | ASP317-GLU320-ASP321 | ['MG'] | Yes | 3ZFZ | A0A0H3JPA5 | ASP35-GLU38-ASP39 | ['MG'] | Yes |
| 2R8R | Q883V3 | LYS16-ASP88-GLU89 | ['MG'] | Yes | 5O7B | Q55535 | ASP138-GLU141-ASP142 | ['MG'] | No |
| 2R9V | Q9X1U7 | ASP474-ASP475-THR478 | ['MG'] | Yes | 4U7D | P46063 | ASP299-GLU302-ASP303 | ['MG'] | No |
| 2V4U | Q9NRF8 | ASP503-ASP505-ASP507 | ['MG'] | Yes | 4IL1 | P0DP29 | ASP423-HIS425-ASP451 | ['ZN'] | Yes |
| 2V7O | Q13555 | GLU140-ASN141-ASP157 | ['MG'] | No | 4IL1 | P0DP29 | ASP451-ASN483-HIS532 | ['MN'] | No |
| 2VK9 | Q46149 | ASP286-GLU520-SER523 | ['CA'] | No | 5MLK | P96890 | GLU285-GLU298-ASN300 | ['CA'] | No |
| 2VQX | Q5MJ80 | TYR177-GLU186-HIS264 | ['ZN'] | Yes | 2RGW | Q58976 | ASP194-GLU197-ASP198 | ['MG'] | No |
| 2WAS | P19097 | ASP1772-GLU1774-GLU1817 | ['MG'] | Yes | 2OVL | Q9RKF7 | ASP197-GLU223-GLU249 | ['MG'] | No |
| 2WBQ | Q6WZB0 | HIS168-GLU170-HIS316 | ['FE'] | Yes | 5BQO | Q97W22 | ASP10-GLU13-ASP14 | ['MG'] | Yes |
| 2WEL | Q13557 | GLU140-ASN141-ASP157 | ['MG'] | Yes | 5D4B | P09838 | ASP343-ASP345-ASP434 | ['MG'] | No |
| 2WFP | P25081 | GLU134-HIS255-HIS99 | ['ZN'] | No | 7CPR | P20478 | GLU140-HIS259-GLU344 | ['MN'] | No |
| 2WK1 | Q9L9F2 | ASP196-ASP223-ASP224 | ['MG'] | Yes | 7CPR | P20478 | GLU142-GLU202-GLU209 | ['MG'] | No |
| 2WOD | P14300 | ASP245-THR59-ASP60 | ['MG'] | Yes | 3U9T | Q9I299 | GLU322-GLU335-ASN337 | ['CA'] | No |
| 2WQK | O67004 | ASP10-ASN94-ASP9 | ['MG'] | Yes | 6HEG | P37024 | ASP123-GLU124-LYS33 | ['MG'] | No |
| 2WRS | Q70E11 | ASP118-CYS198-HIS240 | ['ZN'] | Yes | 2DVM | O59029 | GLU135-ASP136-ASP161 | ['MG'] | Yes |
| 2XKJ | B0V9T6 | ASP1389-ASP1391-THR1392 | ['MG'] | Yes | 2PA6 | Q60173 | ASP248-GLU289-ASP314 | ['MG'] | Yes |
| 2XWH | P26660 | ASP125-GLU128-ASP129 | ['MG'] | Yes | 2PA6 | Q60173 | ASP248-ASP290-ASP314 | ['MG'] | Yes |
| 2XYM | Q99IB8 | ASP125-GLU128-ASP129 | ['MG'] | Yes | 7CV1 | Q9NWX6 | ASP29-ASP76-GLU77 | ['MG'] | No |
| 2Y2C | P82974 | HIS154-ASP164-HIS34 | ['ZN'] | Yes | 5VJA | O43293 | GLU143-ASN144-ASP161 | ['MG'] | Yes |
| 2YAB | Q8VDF3 | GLU143-ASN144-ASP161 | ['MG'] | Yes | 4QKU | B4ECX4 | ASP141-GLU216-ASP248 | ['MG'] | Yes |
| 2YCF | O96017 | GLU351-ASN352-ASP368 | ['MG'] | Yes | 4QKU | B4ECX4 | GLU214-GLU216-ASP248 | ['MG'] | Yes |
| 2YEX | O14757 | GLU134-ASN135-ASP148 | ['MG'] | Yes | 3NYN | P43250 | GLU315-ASN316-ASP329 | ['MG'] | No |
| 2Z0M | Q96XQ7 | ASP136-GLU137-LYS44 | ['MG'] | No | 7TC8 | P18798 | ASP362-GLU365-ASP366 | ['MG'] | No |
| 2ZC8 | Q5SJX8 | ASP188-GLU213-ASP238 | ['MG'] | Yes | 8D45 | Q93088 | CYS217-CYS299-CYS300 | ['ZN'] | No |
| 2ZIU | Q7SXA9 | ASP326-GLU329-ASP361 | ['MG'] | No | 5CFJ | C0H4F3 | GLU115-GLU64-GLU68 | ['MG'] | Yes |
| 2ZQB | Q8EE30 | ASP12-GLU50-ASP72 | ['MG'] | Yes | 6QRO | G8UMP8 | HIS126-ASP151-ASP224 | ['ZN'] | Yes |
| 2ZR9 | Q59560 | ASP112-ASP114-SER115 | ['MG'] | No | 5IG4 | A7T0H5 | ASP365-ASP367-THR368 | ['MG'] | No |
| 3ALY | F9VN79 | ASP125-ASP76-ASP7 | ['MG'] | Yes | 7LIU | O00571 | ASP455-GLU458-ASP459 | ['MG'] | No |
| 3AV0 | Q58719 | HIS10-ASP49-ASP8 | ['ZN'] | No | 3ES8 | Q8EMJ9 | ASP193-GLU221-HIS246 | ['MG'] | Yes |
| 3AV0 | Q58719 | HIS158-HIS186-ASP49 | ['ZN'] | Yes | 3I4Q | D0VWZ3 | ASP103-ASP66-ASP71 | ['MG'] | Yes |
| 3B8B | Q8AAQ3 | GLU61-ASP81-ASP84 | ['MG'] | Yes | 2EPG | Q5SHE5 | HIS204-ASP95-CYS98 | ['MN'] | Yes |
| 3BER | Q9H0S4 | ASP174-GLU175-LYS74 | ['MG'] | Yes | 3RR1 | B2UCA8 | ASP183-GLU209-GLU235 | ['MG'] | No |
| 3BHD | Q9BU02 | GLU159-GLU7-GLU9 | ['MN'] | Yes | 3RR1 | B2UCA8 | GLU209-GLU235-HIS285 | ['MG'] | Yes |
| 3BHY | O43293 | GLU143-ASN144-ASP161 | ['MG'] | No | 5NQA | Q8N4A0 | ASP227-HIS229-HIS362 | ['MN'] | Yes |
| 3BJ7 | P48026 | ASP40-GLU43-ASP44 | ['MG'] | No | 3SY8 | Q9HX69 | ASP295-ASP296-GLU352 | ['MG'] | No |
| 3C4B | Q8R418 | GLU1689-ASP1794-GLU1797 | ['MG'] | Yes | 4ZNG | A8WBX8 | ASP232-HIS296-GLU339 | ['MN'] | No |
| 3CLH | P56081 | GLU174-HIS231-HIS248 | ['ZN'] | No | 3JUJ | O25363 | ASP130-ASP131-ASP254 | ['MG'] | Yes |
| 3CP2 | P0A6U3 | ASP415-ASP416-THR419 | ['MG'] | No | 4UMP | Q14680 | GLU136-ASN137-ASP150 | ['MG'] | Yes |
| 3CRA | P0AEY3 | GLU39-GLU58-ASP61 | ['MG'] | No | 2UX8 | Q5FYV5 | ASP133-ASP134-ASP255 | ['MG'] | No |
| 3CRV | Q4JC68 | ASP253-GLU256-ASP257 | ['MG'] | Yes | 6OPM | A0A0F8IEL4 | HIS14-ASP16-HIS21 | ['ZN'] | Yes |
| 3CTZ | Q9NQW7 | ASP573-ASP575-SER576 | ['MG'] | No | 6WNI | U5CJP3 | ASP328-GLU331-ASP332 | ['MG'] | No |
| 3D0C | Q8EMJ7 | ASP238-GLU241-ASP242 | ['MG'] | Yes | 4JKM | Q8XP19 | ASP414-ASP416-SER417 | ['MG'] | No |
| 3D2Y | P75820 | HIS151-ASP161-HIS35 | ['ZN'] | No | 4OA5 | Q2GKC7 | ASP136-ASP162-ASN163 | ['MG'] | Yes |
| 3DTE | C1CZ84 | GLU113-HIS82-HIS86 | ['ZN'] | No | 2P3N | O33832 | ASP201-ASP79-ASP82 | ['MG'] | Yes |
| 3DUW | Q739U3 | ASP140-ASP166-ASN167 | ['MG'] | Yes | 4LT6 | Q9BWT3 | ASP112-ASP114-ASP166 | ['MG'] | No |
| 3DZB | Q5M554 | ASP248-GLU251-ASP252 | ['MG'] | No | 1RTU | P00654 | ASP17-ASP18-THR21 | ['MG'] | Yes |
| 3FE2 | P17844 | LYS144-ASP248-GLU249 | ['MG'] | Yes | 5ZLP | P94845 | GLU139-HIS279-GLU367 | ['MN'] | No |
| 3FHR | Q16644 | GLU170-ASN171-ASP187 | ['MG'] | Yes | 5ZLP | P94845 | GLU141-GLU223-GLU230 | ['MG'] | Yes |
| 3GB0 | Q731F0 | ASP107-GLU141-HIS345 | ['ZN'] | Yes | 4BPT | Q5ZS72 | HIS122-HIS127-GLU167 | ['FE'] | Yes |
| 3GB0 | Q731F0 | ASP107-ASP164-HIS77 | ['MN'] | Yes | 6Q2D | A5UMY5 | ASP136-GLU139-ASP140 | ['MG'] | No |
| 3GC2 | P58486 | ASP161-GLU190-ASP213 | ['MG'] | Yes | 4KIK | O14920 | GLU149-ASN150-ASP166 | ['MG'] | No |
| 3GD6 | Q8EMJ9 | ASP193-GLU221-HIS246 | ['MG'] | Yes | 6ZJB | Q9UIJ7 | ASP68-ASP69-THR72 | ['MG'] | Yes |
| 3GQM | Q63KH5 | ASP208-ASP209-THR212 | ['MG'] | No | 5CUU | Q4JH30 | ASP291-ASP296-ASP328 | ['MG'] | No |
| 3GRN | Q8PYE2 | GLU104-GLU58-GLU62 | ['MG'] | No | 1N8P | P31373 | ASP379-GLU382-ASP383 | ['MG'] | No |
| 3GVF | Q3JUV5 | ASP103-ASP66-ASP71 | ['MG'] | Yes | 6BRH | Q60710 | ASP116-GLU119-ASP120 | ['MG'] | No |
| 3GVY | Q3J696 | GLU127-GLU51-GLU94 | ['FE'] | No | 6IKA | D3XFN7 | ASP110-ASP185-ASP186 | ['MG'] | No |
| 3H4X | B3A043 | GLU129-GLU60-ASP62 | ['MG'] | Yes | 6IKA | D3XFN7 | ASP443-GLU478-ASP498 | ['MG'] | No |
| 3H7C | Q8GWW7 | ASP218-ASP220-THR221 | ['MG'] | Yes | 6IKA | D3XFN7 | ASP443-ASP498-ASP549 | ['MG'] | No |
| 3HJ4 | C7AJA4 | ASP130-ASP65-ASP67 | ['MG'] | Yes | 2HWT | O39828 | GLU33-HIS41-GLN43 | ['MN'] | No |
| 3HPO | D9N168 | ASP653-ASP830-GLU831 | ['MG'] | Yes | 4HN8 | A4XRL3 | ASP252-GLU277-ASN306 | ['MG'] | Yes |
| 3HYH | P06782 | GLU181-ASN182-ASP195 | ['MG'] | No | 5C05 | A0A0M3Q1Q3 | ASP493-ASP494-THR497 | ['MG'] | No |
| 3IUZ | Q46PQ5 | HIS237-GLU303-HIS81 | ['ZN'] | Yes | 4IOB | Q9I4L5 | ASP287-ASP330-GLU331 | ['MG'] | No |
| 3JRQ | P49597 | ASP177-ASP347-ASP413 | ['MG'] | Yes | 6UOK | P06746 | ASP190-ASP192-ASP256 | ['MG'] | No |
| 3KZN | Q8P8J2 | ASP238-ASP240-SER241 | ['MG'] | No | 5K52 | A0A193CDY9 | GLU32-GLU60-HIS63 | ['FE'] | No |
| 3L5K | Q08623 | ASP14-GLU175-ASP176 | ['MG'] | No | 3TO3 | Q81RQ8 | GLN438-ASN439-ASP456 | ['MG'] | No |
| 3LD3 | Q2GJ02 | ASP103-ASP66-ASP71 | ['MG'] | Yes | 7SKB | V5VGT0 | ASP289-GLU292-ASP293 | ['MG'] | Yes |
| 3LFU | P03018 | ASP220-GLU221-LYS35 | ['MG'] | No | 3V15 | Q88RA3 | HIS253-HIS97-ASP99 | ['ZN'] | No |
| 3LLT | Q8IL19 | GLU681-ASN682-ASP720 | ['MG'] | Yes | 2Y7J | P15735 | GLU157-ASN158-ASP171 | ['MG'] | Yes |
| 3LN6 | Q8DXM9 | HIS144-GLU24-GLU280 | ['MN'] | No | 1CF2 | P10618 | ASP238-GLU241-ASP242 | ['MG'] | No |
| 3LN6 | Q8DXM9 | GLU26-ASP58-GLU65 | ['MG'] | No | 2PK9 | P17157 | GLN137-ASN138-ASP151 | ['MG'] | No |
| 3LV0 | A0A0H3M6W8 | ASP214-ASP86-ASP89 | ['MG'] | Yes | 2WOE | P14300 | ASP245-THR59-ASP60 | ['MG'] | Yes |
| 3M2W | P49137 | GLU190-ASN191-ASP207 | ['MG'] | Yes | 6PK4 | Q9NRF8 | ASP503-ASP505-ASP507 | ['MG'] | No |
| 3MSR | Q4A724 | HIS24-HIS26-ASP272 | ['ZN'] | Yes | 2IVO | Q9UXT7 | HIS107-HIS111-ASP285 | ['ZN'] | No |
| 3N5F | Q53389 | GLU125-HIS380-ASP90 | ['ZN'] | No | 1OZ9 | O67367 | HIS115-HIS119-HIS125 | ['ZN'] | No |
| 3NAS | O06995 | GLU169-ASP170-ASP7 | ['MG'] | No | 5DMX | B2I1J3 | GLU183-ASP255-GLU268 | ['MG'] | No |
| 3NZT | Q5NHS8 | HIS137-GLU14-GLU309 | ['MN'] | Yes | 7CVU | G8T6H8 | ASP139-ASP165-ASN166 | ['MG'] | No |
| 3NZT | Q5NHS8 | GLU16-ASP47-GLU54 | ['MG'] | Yes | 4S17 | A1A1Z1 | GLU134-HIS277-GLU367 | ['MN'] | No |
| 3O2R | Q9PM40 | ASP113-GLU116-GLU40 | ['MG'] | Yes | 4C3O | Q8ZPH0 | CYS579-CYS582-CYS76 | ['NI'] | No |
| 3OF5 | Q5NGB5 | GLU114-THR16-ASP52 | ['MG'] | Yes | 6DRH | A8GG79 | THR116-ASP117-ASP317 | ['MG'] | Yes |
| 3OGA | Q8ZNF5 | GLU110-GLU53-GLU57 | ['MG'] | Yes | 6OJM | A0A077EJG6 | ASP49-GLU52-ASP53 | ['MG'] | Yes |
| 3OKF | Q9KNV2 | GLU188-HIS251-HIS268 | ['ZN'] | No | 1J1W | P16100 | ASP350-ASP548-ASP552 | ['MG'] | No |
| 3OTD | Q9NWX6 | ASP29-ASP76-GLU77 | ['MG'] | No | 5OEZ | O97193 | ASP118-ASP121-GLU284 | ['MG'] | No |
| 3OVA | O28126 | ASP110-GLU59-ASP61 | ['MG'] | Yes | 5OEZ | O97193 | ASP118-GLU284-GLU97 | ['MG'] | No |
| 3P5J | Q9CWY8 | ASP142-ASP34-GLU35 | ['MG'] | No | 7PK5 | P21514 | GLU141-ASN200-ASP262 | ['MG'] | No |
| 3PB6 | Q9NXS2 | HIS168-ASP186-ASP269 | ['ZN'] | No | 7PK5 | P21514 | ASP262-ASP263-GLU319 | ['MG'] | No |
| 3QBE | P9WPX9 | ASP28-GLU31-ASP32 | ['MG'] | No | 2JLE | Q72547 | ASP443-GLU478-ASP498 | ['MG'] | No |
| 3R3H | Q5ZT85 | ASP143-ASP169-ASN170 | ['MG'] | No | 6VGR | Q9H4B8 | GLU196-HIS97-ASP99 | ['ZN'] | No |
| 3RCM | Q88KH9 | HIS158-CYS159-GLU207 | ['ZN'] | Yes | 7OGP | L7T138 | ASP417-ASP419-ASP421 | ['MG'] | No |
| 3RL5 | B1WBP0 | ASN117-HIS213-ASP86 | ['MN'] | Yes | 3A2C | P49137 | GLU190-ASN191-ASP207 | ['MG'] | No |
| 3RQ4 | Q86Y97 | ASP15-ASP16-THR19 | ['MG'] | Yes | 8DNU | P15104 | GLU136-GLU196-GLU203 | ['MG'] | No |
| 3S5S | A9GEI3 | ASP194-GLU222-ASP247 | ['MG'] | No | 3CP8 | Q8KA85 | ASP411-ASP412-THR415 | ['MG'] | No |
| 3S6B | Q8IJP2 | ASP193-ASP204-GLU334 | ['MG'] | Yes | 4E80 | O13833 | ASP101-ASP103-ASP160 | ['MG'] | No |
| 3S8P | Q4FZB7 | ASP81-ASP82-THR85 | ['MG'] | Yes | 7BMM | Q9RY20 | ASP208-GLU211-ASP212 | ['MG'] | Yes |
| 3SDB | P9WJJ3 | ASP71-GLU74-ASP75 | ['MG'] | Yes | 6KI7 | A0A0A7XPH7 | ASP102-ASP65-ASP70 | ['CA'] | No |
| 3SI2 | Q9CYK2 | HIS141-ASP160-ASP249 | ['ZN'] | No | 2OHJ | Q50497 | ASP170-HIS83-GLU85 | ['FE'] | No |
| 3TC3 | Q4J9T1 | HIS100-HIS141-GLU174 | ['ZN'] | Yes | 2DFV | O58389 | CYS42-HIS67-GLU68 | ['ZN'] | Yes |
| 3TX8 | Q59284 | ASP37-GLU40-ASP41 | ['MG'] | No | 6ODB | Q9BY41 | ASP183-GLU186-ASP187 | ['MG'] | No |
| 3TY2 | Q9KI21 | ASN102-ASP19-ASP20 | ['MG'] | Yes | 5EQA | G7J7Q5 | GLU128-ASP146-ASP149 | ['MG'] | Yes |
| 3TYP | Q82UI9 | GLU116-GLU4-GLU6 | ['MG'] | Yes | 5EQA | G7J7Q5 | ASP146-ASP149-ASP270 | ['MG'] | No |
| 3U9I | A5UXJ3 | ASP203-GLU231-ASP256 | ['MG'] | Yes | 3PVT | P76077 | GLU42-GLU72-HIS75 | ['FE'] | Yes |
| 3UAR | Q60CN1 | ASP134-GLU137-ASP138 | ['MG'] | No | 4BAX | Q9X958 | GLU119-HIS237-GLU318 | ['MN'] | No |
| 3VJ8 | P37268 | ASP80-GLU83-ASP84 | ['MG'] | No | 4BAX | Q9X958 | GLU121-GLU180-GLU187 | ['MG'] | Yes |
| 3VN5 | O67644 | ASP179-ASP77-GLU78 | ['MG'] | No | 5LP3 | P39377 | ASP285-HIS68-HIS70 | ['ZN'] | No |
| 3ZDU | Q8IVW4 | GLU129-ASN130-ASP143 | ['MG'] | Yes | 4IS4 | O04998 | GLU129-HIS249-GLU330 | ['MN'] | Yes |
| 4A15 | Q9HM14 | ASP211-GLU212-LYS35 | ['MG'] | No | 4IS4 | O04998 | GLU131-GLU192-GLU199 | ['MG'] | No |
| 5X0J | Q5JD03 | ASP184-GLU187-ASP188 | ['MG'] | No | 3WCE | Q4CWB4 | ASP71-GLU74-ASP75 | ['MG'] | No |
| 5XZG | Q8C6L5 | GLU211-ASP213-ASP307 | ['MG'] | No | 4KJD | P15693 | ASP357-HIS358-ASP42 | ['ZN'] | Yes |
| 5Y86 | O43781 | GLU339-ASN340-ASP355 | ['MG'] | Yes | 7CQL | A0A369R1N0 | GLU122-HIS235-GLU325 | ['MN'] | No |
| 5Z4A | Q9VI58 | ASP278-ASP280-ASP343 | ['MG'] | No | 7CQL | A0A369R1N0 | GLU124-GLU179-GLU186 | ['MG'] | No |
| 5ZFS | A0A1L7NQ96 | ASP220-ASP222-THR223 | ['MG'] | Yes | 3NIP | Q9I6K2 | HIS126-ASP148-ASP152 | ['MN'] | No |
| 5ZTP | A0A384E115 | CYS38-HIS90-CYS93 | ['ZN'] | No | 6LVE | I6NT79 | ASP529-GLU532-ASP533 | ['MG'] | No |
| 5ZW4 | O32036 | ASP133-ASP159-ASN160 | ['MG'] | Yes | 1XW4 | Q9BYN0 | ASP72-ASP74-SER75 | ['MG'] | No |
| 6AGS | P26616 | GLU246-ASP247-ASP270 | ['MG'] | No | 7K74 | B2FU10 | ASP107-ASP110-GLU85 | ['MG'] | Yes |
| 6AW6 | A0A452CSQ2 | ASP140-ASP168-ASN169 | ['MG'] | Yes | 1OQ7 | P22337 | GLU196-GLU229-HIS232 | ['FE'] | No |
| 6BRK | Q60710 | ASP116-GLU119-ASP120 | ['MG'] | No | 6G33 | P49759 | GLU292-ASN293-ASP325 | ['MG'] | Yes |
| 6BU2 | L7N6B1 | GLU146-HIS23-GLN96 | ['ZN'] | No | 7ZSC | O15460 | HIS430-ASP432-HIS501 | ['ZN'] | No |
| 6BU2 | L7N6B1 | GLU50-ASN52-GLU59 | ['CA'] | No | 5DT5 | K0A8J9 | ASP418-ASP420-THR421 | ['MG'] | No |
| 6BWG | P9WP83 | ASP116-ASP188-ASP190 | ['MG'] | Yes | 4L0O | P56069 | ASP367-GLU370-ASP371 | ['MG'] | No |
| 6C56 | O95749 | ASP64-GLU67-ASP68 | ['MG'] | No | 6G3U | Q9I0L4 | ASP350-ASP548-ASP552 | ['MG'] | Yes |
| 6C5C | Q5AME2 | GLU191-HIS264-HIS280 | ['ZN'] | No | 5IUF | O34600 | HIS20-ASP24-ASP80 | ['MN'] | Yes |
| 6C5C | Q5AME2 | ASP56-ASP57-THR60 | ['MG'] | No | 4O6H | P09992 | ASP382-GLU384-ASP522 | ['MG'] | No |
| 6D5A | P9WKV3 | ASP233-ASP235-HIS256 | ['CD'] | No | 2P0I | D0VX14 | ASP221-GLU247-GLU274 | ['MG'] | No |
| 6DA7 | C6ZCR8 | ASN164-HIS187-GLU228 | ['MN'] | Yes | 8ECC | Q8C6L5 | GLU211-ASP213-ASP307 | ['MG'] | No |
| 6DNO | P62136 | ASN124-HIS173-ASP92 | ['MN'] | Yes | 5DUL | Q8ZH62 | ASP150-GLU152-GLU231 | ['MG'] | No |
| 6DNO | P62136 | HIS173-HIS248-ASP92 | ['ZN'] | Yes | 3FPI | Q8ZBP7 | HIS10-HIS44-ASP8 | ['ZN'] | Yes |
| 6DNO | P62136 | ASP64-HIS66-ASP92 | ['ZN'] | Yes | 3GRI | P65907 | ASP379-ASP381-SER382 | ['MG'] | Yes |
| 6DRE | A8GG79 | ASP315-ASP317-SER318 | ['MG'] | Yes | 7B6N | P22188 | THR121-THR143-GLU183 | ['MG'] | No |
| 6DSW | E1C9K5 | ASP653-ASP830-GLU831 | ['MG'] | Yes | 1OIH | Q9WWU5 | HIS108-ASP110-HIS264 | ['ZN'] | No |
| 6DX9 | Q9MBB1 | ASP260-HIS262-HIS271 | ['ZN'] | No | 6WC8 | F2WR52 | CYS65-HIS67-GLU92 | ['CD'] | No |
| 6DXA | P30079 | ASP260-HIS262-HIS271 | ['ZN'] | No | 2PCR | O67791 | ASP214-ASP86-ASP89 | ['MG'] | Yes |
| 6DXE | P13114 | ASP261-HIS263-HIS272 | ['ZN'] | Yes | 3MWP | P13699 | ASP389-GLU391-ASP533 | ['MG'] | Yes |
| 6DZD | A5A677 | CYS132-HIS149-HIS87 | ['ZN'] | Yes | 5GWO | Q6L5H6 | ASP161-ASP162-THR165 | ['MG'] | No |
| 6E0K | G2SLH8 | ASP280-ASP281-THR284 | ['MG'] | Yes | 3AEU | P26164 | ASP181-GLU184-ASP185 | ['MG'] | No |
| 6E0M | A0A4V8GZR7 | ASP136-ASP61-ASP63 | ['MG'] | Yes | 6STY | Q9Y3B8 | ASP199-ASP47-GLU49 | ['MG'] | Yes |
| 6E0N | A0A4V8GZR7 | ASP136-ASP61-ASP63 | ['MG'] | Yes | 1IS7 | P22288 | CYS132-HIS135-CYS203 | ['ZN'] | No |
| 6EVG | A6X5N0 | HIS437-HIS439-HIS489 | ['MG'] | No | 3LN7 | Q9CM00 | GLU578-ASP702-GLU723 | ['MG'] | No |
| 6FJW | A0A0A7HF73 | ASP66-GLU69-ASP70 | ['MG'] | No | 3LN7 | Q9CM00 | HIS144-GLU24-GLU285 | ['MN'] | No |
| 6FT8 | P49759 | GLU292-ASN293-ASP325 | ['MG'] | Yes | 1BXC | P56681 | GLU180-GLU216-ASP286 | ['MN'] | No |
| 6FYL | P49760 | GLU294-ASN295-ASP327 | ['MG'] | Yes | 1BXC | P56681 | GLU180-ASP244-ASP286 | ['MG'] | No |
| 6FYR | P49761 | GLU287-ASN288-ASP320 | ['MG'] | Yes | 1BXC | P56681 | GLU216-HIS219-ASP254 | ['MG'] | Yes |
| 6FYV | Q9HAZ1 | GLU292-ASN293-ASP325 | ['MG'] | No | 1BXC | P56681 | GLU216-ASP254-ASP256 | ['MG'] | Yes |
| 6G1P | H3BCW1 | ASP303-ASP305-THR306 | ['MG'] | Yes | 8FEC | P17612 | GLU225-ASN226-ASP239 | ['MG'] | No |
| 6GU2 | P06493 | GLN132-ASN133-ASP146 | ['MG'] | No | 4KYI | Q5ZRP9 | ASP475-GLU478-ASP479 | ['MG'] | No |
| 6H8O | O39828 | GLU33-HIS41-GLN43 | ['MN'] | Yes | 5T8Y | P97030 | ASP114-GLU117-ASP118 | ['MG'] | No |
| 6HEI | Q96RU2 | ASP265-GLU268-ASP269 | ['MG'] | Yes | 3HOA | Q72GY3 | HIS276-HIS280-GLU306 | ['ZN'] | Yes |
| 6IB8 | P0ADG4 | ASP212-ASP84-ASP87 | ['MG'] | Yes | 5OU5 | P16243 | GLU327-ASP328-ASP351 | ['MG'] | No |
| 6IFQ | A0A0H3AJ04 | ASP158-ASP159-GLU215 | ['MG'] | No | 1MWU | Q93IC2 | ASP35-GLU38-ASP39 | ['MG'] | No |
| 6IG4 | O74339 | ASP164-GLU167-ASP168 | ['MG'] | Yes | 1YDO | O34873 | ASP16-HIS207-HIS209 | ['ZN'] | No |
| 6IUX | P54922 | ASP302-ASP304-SER305 | ['MG'] | No | 4PXD | P77425 | HIS190-HIS81-ASP92 | ['ZN'] | No |
| 6JTZ | P46063 | ASP299-GLU302-ASP303 | ['MG'] | No | 5OET | A0A183C5H8 | ASP168-GLU185-GLU474 | ['MG'] | No |
| 6JY1 | Q60358 | ASP164-GLU167-ASP168 | ['MG'] | Yes | 3CF4 | Q46G04 | ASP260-GLU263-ASP264 | ['MG'] | Yes |
| 6JYV | Q9HWJ0 | HIS201-ASP203-HIS257 | ['ZN'] | Yes | 3T6B | Q9NY33 | HIS450-HIS455-GLU508 | ['ZN'] | No |
| 6KC0 | B6YWB8 | ASP101-ASP15-HIS201 | ['MN'] | No | 2P76 | A0A0H3JTK7 | GLU169-GLU87-ASP89 | ['MG'] | No |
| 6KPL | G3JPF7 | ASP152-ASP154-ASP191 | ['MG'] | No | 5O6Y | Q81EK9 | HIS131-HIS135-ASP81 | ['ZN'] | No |
| 6KSF | P0CB42 | HIS231-ASP233-HIS287 | ['ZN'] | No | 3GT7 | Q2LQE8 | GLU18-ASP19-ASP62 | ['MG'] | Yes |
| 6KUN | Q01IX6 | HIS173-ASP175-HIS232 | ['ZN'] | No | 6AR1 | E2GM63 | ASP138-ASP223-ASP224 | ['MG'] | No |
| 6L08 | O65896 | CYS104-CYS107-HIS77 | ['ZN'] | No | 5ZIG | E0RU15 | ASP150-GLU153-ASP154 | ['MG'] | Yes |
| 6LBK | Q5U315 | SER202-ASP213-ASP215 | ['MG'] | No | 6JCM | O07431 | ASP139-ASP165-ASN166 | ['MG'] | No |
| 6M37 | P17904 | ASP38-GLU41-ASP42 | ['MG'] | No | 2UZ3 | Q9PPP5 | LYS25-ASP96-GLU97 | ['MG'] | No |
| 6MRF | V5VCW7 | ASP100-ASP111-GLU239 | ['MG'] | Yes | 1IAX | P18485 | ASP61-GLU64-ASP65 | ['MG'] | No |
| 6N6A | Q9KV17 | ASP12-GLU14-ASP163 | ['MG'] | Yes | 7DWQ | B0C474 | ASP58-ASP60-THR61 | ['MG'] | No |
| 6N6A | Q9KV17 | GLU14-HIS158-ASP163 | ['MG'] | Yes | 4TUI | Q58719 | HIS10-HIS188-ASP8 | ['ZN'] | No |
| 6N6J | Q9Y3B8 | ASP15-ASP167-GLU17 | ['MG'] | Yes | 4TUI | Q58719 | HIS10-ASP49-ASP8 | ['ZN'] | No |
| 6NIB | G7JT50 | ASP218-ASP220-THR221 | ['MG'] | Yes | 3WFS | O67911 | ASP112-ASP58-ASP60 | ['MG'] | No |
| 6NOZ | W8QLX4 | ASP206-ASP208-THR209 | ['MG'] | Yes | 3WFS | O67911 | HIS276-HIS325-ASP418 | ['ZN'] | No |
| 6P5S | Q9H2X6 | GLU328-ASN329-ASP346 | ['MG'] | No | 3WFS | O67911 | HIS276-ASP326-ASP418 | ['MN'] | No |
| 6P80 | D7Y2H2 | ASP130-ASP72-ASP74 | ['MG'] | Yes | 3WFS | O67911 | HIS325-ASP326-ASP418 | ['ZN'] | No |
| 6RA7 | Q9UKE5 | GLN157-ASN158-ASP171 | ['MG'] | Yes | 5FHH | Q9H611 | LYS234-ASP306-GLU307 | ['MG'] | No |
| 6RB4 | P49642 | ASP109-ASP111-HIS166 | ['CD'] | Yes | 5ET6 | O00757 | ASP118-ASP121-GLU280 | ['MG'] | Yes |
| 6S1Y | A0NFU8 | ASP320-ASP321-THR324 | ['MG'] | No | 5OUW | Q0I9X8 | GLU33-GLU66-HIS69 | ['FE'] | Yes |
| 6STX | E3Q9X3 | TYR533-HIS534-HIS632 | ['CU'] | No | 4EWJ | K7N5M7 | ASP243-GLU292-ASP319 | ['MG'] | Yes |
| 6VDC | A0QYZ2 | ASP684-ASP861-GLU862 | ['MG'] | No | 4EWJ | K7N5M7 | ASP243-ASP293-ASP319 | ['MG'] | Yes |
| 6VGO | Q9H4B8 | GLU196-HIS97-ASP99 | ['ZN'] | Yes | 3GTT | P08228 | HIS120-HIS46-HIS48 | ['ZN'] | Yes |
| 6VOP | Q46890 | HIS100-HIS165-HIS98 | ['ZN'] | Yes | 6MM7 | P05132 | GLU170-ASN171-ASP184 | ['MG'] | Yes |
| 6VPM | O14965 | GLU260-ASN261-ASP274 | ['MG'] | No | 5M4X | Q9HK01 | ASP47-GLU50-ASP51 | ['MG'] | No |
| 6VVC | Q5ZSB6 | GLN212-ASN213-ASP225 | ['MG'] | Yes | 5KVU | O53611 | ASP352-ASP551-ASP555 | ['MG'] | No |
| 6WE5 | O84777 | ASP130-ASP92-ASP97 | ['MG'] | Yes | 3I12 | P0A1F0 | GLU228-ASP302-GLU315 | ['MG'] | Yes |
| 6WKC | Q55012 | ASN219-SER223-GLU227 | ['MG'] | Yes | 3OID | P71079 | ASP200-GLU203-ASP204 | ['MG'] | No |
| 6WNU | U5CJP3 | ASP328-GLU331-ASP332 | ['MG'] | Yes | 1O0S | P27443 | GLU271-ASP272-ASP295 | ['MG'] | Yes |
| 6X5G | Q9UQM7 | GLU139-ASN140-ASP156 | ['MG'] | Yes | 3NV9 | Q9NH04 | GLU168-ASP169-ASP194 | ['MG'] | Yes |
| 6XBU | O75417 | ASP2330-ASP2540-GLU2541 | ['MG'] | No | 3DLA | P9WJJ3 | ASP71-GLU74-ASP75 | ['MG'] | Yes |
| 6XHK | Q2G1B9 | GLU144-HIS64-GLU65 | ['MN'] | No | 1E5D | Q9F0J6 | ASP165-HIS79-GLU81 | ['FE'] | No |
| 6XIA | P24299 | GLU180-ASP244-ASP286 | ['MG'] | Yes | 4CZT | Q93VD3 | GLU158-ASN159-ASP172 | ['MG'] | Yes |
| 6XIA | P24299 | GLU216-HIS219-ASP254 | ['MG'] | Yes | 1QPR | P9WJJ7 | GLU201-ASP203-ASP222 | ['MG'] | Yes |
| 6XIA | P24299 | GLU216-ASP254-ASP256 | ['MG'] | Yes | 3NH2 | P30014 | ASP186-ASP23-GLU25 | ['MG'] | Yes |
| 6YWN | G2R014 | ASP339-ASP341-ASP392 | ['MG'] | Yes | 2IS1 | P03018 | ASP220-GLU221-LYS35 | ['MG'] | No |
| 6ZD1 | C5MCQ7 | ASP515-ASP678-ASP679 | ['MG'] | No | 7YBU | P05165 | GLU336-GLU349-ASN351 | ['CA'] | No |
| 6ZJE | Q9UIJ7 | ASP68-ASP69-THR72 | ['MG'] | Yes | 3RAW | P49761 | GLU287-ASN288-ASP320 | ['MG'] | Yes |
| 6ZJF | O94768 | GLN162-ASN163-ASP179 | ['MG'] | Yes | 3GM5 | Q8RCQ6 | GLN13-GLU74-HIS98 | ['ZN'] | No |
| 7A7E | Q9I4L5 | ASP287-ASP330-GLU331 | ['MG'] | Yes | 1WOH | Q9RZ04 | HIS121-ASP143-ASP147 | ['MN'] | No |
| 7AFU | Q96SD1 | ASP136-ASP37-HIS38 | ['ZN'] | No | 1WOH | Q9RZ04 | HIS121-ASP143-ASP229 | ['MN'] | No |
| 7AO3 | C9K1X5 | ASP259-GLU262-ASP263 | ['MG'] | Yes | 1WOH | Q9RZ04 | ASP143-ASP147-ASP229 | ['MN'] | No |
| 7D17 | A0A267GXB9 | ASP335-GLU338-ASP339 | ['MG'] | No | 3KYH | O13297 | GLU305-GLU307-GLU496 | ['MN'] | No |
| 7D7O | Q818A3 | ASP367-GLU370-ASP371 | ['MG'] | No | 3ANM | P45568 | ASP149-GLU151-GLU230 | ['MG'] | No |
| 7DA2 | E1BSW7 | ASP33-GLU36-ASP37 | ['MG'] | Yes | 6KU3 | Q8S0S6 | HIS202-ASP204-HIS259 | ['ZN'] | No |
| 7E8J | A0A218P4J1 | ASP140-GLU143-ASP144 | ['MG'] | No | 3QYR | Q73VV7 | ASP208-ASP209-THR212 | ['MG'] | Yes |
| 7EKD | Q9FU53 | HIS227-ASP229-HIS285 | ['ZN'] | Yes | 3WKS | Q59072 | ASP81-GLU84-ASP85 | ['MG'] | No |
| 7FC3 | F6V9L3 | HIS374-HIS378-GLU402 | ['ZN'] | No | 4HCB | P04995 | ASP15-GLU17-ASP186 | ['MG'] | No |
| 7KSF | A0A1Q1N9V8 | ASP254-ASP316-ASP317 | ['MG'] | No | 2EIV | Q5SI78 | ASP119-HIS121-ASP220 | ['ZN'] | Yes |
| 7KVY | J3KJC6 | ASP53-ASP55-THR56 | ['MG'] | No | 2EIV | Q5SI78 | ASP119-ASP123-ASP218 | ['MN'] | Yes |
| 7LGP | P0AED8 | GLU134-HIS348-ASP99 | ['ZN'] | Yes | 2EIV | Q5SI78 | ASP119-ASP123-HIS97 | ['MN'] | Yes |
| 7LPZ | O43414 | ASP150-GLU152-ASP312 | ['MG'] | No | 2EIV | Q5SI78 | ASP119-ASP218-HIS97 | ['MN'] | Yes |
| 7LT2 | D6WI29 | ASP193-GLU70-ASP72 | ['MG'] | Yes | 3PDK | Q81VN7 | SER100-ASP240-ASP242 | ['CD'] | No |
| 7NWA | P54687 | ASP314-ASP315-THR318 | ['MG'] | Yes | 5QHH | P0C024 | GLU141-GLU93-GLU97 | ['MG'] | Yes |
| 7O7I | Q9H422 | GLU326-ASN327-ASP344 | ['MG'] | No | 5QI5 | Q460N5 | ASP1349-GLU1352-ASP1353 | ['MG'] | Yes |
| 7P5T | P9WPL5 | ASP157-ASP158-THR161 | ['MG'] | No | 5TC9 | G0RVK1 | ASP328-ASP329-THR332 | ['MG'] | Yes |
| 7PCR | P56185 | ASP212-HIS213-HIS526 | ['ZN'] | No | 5U4N | Q5FAI4 | HIS198-HIS232-HIS84 | ['ZN'] | No |
| 7Q73 | Q10295 | ASP108-ASP110-SER97 | ['MG'] | Yes | 5UH7 | P9WGY7 | ASP115-GLU118-ASP119 | ['MG'] | No |
| 7QUE | Q9UEE5 | GLN190-ASN191-ASP207 | ['MG'] | No | 5UWZ | Q7NGM3 | GLU33-GLU61-HIS64 | ['FE'] | Yes |
| 7RSF | P23908 | ASP112-GLU145-HIS355 | ['ZN'] | Yes | 5UXI | B2J1M1 | GLU33-GLU61-HIS64 | ['FE'] | No |
| 7SEZ | P04311 | GLU126-GLU130-GLU183 | ['MG'] | Yes | 5VI6 | Q9BY41 | ASP183-GLU186-ASP187 | ['MG'] | Yes |
| 7SJ3 | P11802 | GLU144-ASN145-ASP158 | ['MG'] | No | 5VJE | P0AB71 | HIS110-HIS226-HIS264 | ['ZN'] | No |
| 7SKL | P81177 | TYR367-GLU376-HIS436 | ['ZN'] | Yes | 1HFB | P32449 | HIS282-ASP342-CYS76 | ['MN'] | Yes |
| 7SXF | P49840 | GLN248-ASN249-ASP263 | ['MG'] | No | 6JU6 | A0A1S9DK56 | HIS103-HIS67-HIS94 | ['ZN'] | Yes |
| 7SXJ | P49841 | GLN185-ASN186-ASP200 | ['MG'] | Yes | 6JU6 | A0A1S9DK56 | HIS328-HIS332-HIS372 | ['ZN'] | Yes |
| 7SXJ | P49841 | ASP190-ASP192-THR193 | ['MG'] | No | 3OWG | P23371 | SER188-ASP202-ASP204 | ['MG'] | No |
| 7T7N | O94725 | ASP252-ASN253-ASP290 | ['MG'] | Yes | 3OWG | P23371 | ASP293-GLU296-ASP297 | ['MG'] | No |
| 7TA5 | B7KEP8 | ASP321-GLU324-ASP325 | ['MG'] | No | 8FF8 | P49841 | GLN185-ASN186-ASP200 | ['MG'] | No |
| 7TQO | Q9NSU2 | ASP18-ASP200-GLU20 | ['MG'] | Yes | 8FF8 | P49841 | ASP190-ASP192-THR193 | ['MG'] | Yes |
| 7UOI | A0A1S8KJG1 | ASP104-GLU139-HIS352 | ['ZN'] | Yes | 4PN1 | Q9P6Q6 | GLU262-GLU78-GLU80 | ['MG'] | Yes |
| 7V4Q | D7RF80 | LYS205-ASP290-GLU291 | ['MG'] | No | 8D9D | P49642 | ASP109-ASP111-HIS166 | ['CD'] | No |
| 7WGH | A0A364LX79 | ASP81-GLU84-ASP85 | ['MG'] | No | 7S77 | P36871 | ASP288-ASP290-ASP292 | ['MG'] | No |
| 7XS4 | O64642 | SER480-ASP491-ASP493 | ['MG'] | Yes | 3NZP | Q0PAC6 | ASP558-GLU561-ASP562 | ['MG'] | No |
| 7XWY | O42861 | LYS418-ASP518-GLU519 | ['MG'] | Yes | 1POX | P37063 | ASP100-ASP96-GLU99 | ['MG'] | Yes |
| 7XZ4 | K3ZJM0 | ASP172-GLU175-ASP176 | ['MG'] | No | 1POX | P37063 | ASP482-GLU485-ASP486 | ['MG'] | No |
| 7Y1U | A0A7M1LFL5 | ASP343-ASP541-ASP545 | ['MG'] | Yes | 4FIP | P50102 | ASP119-ASP120-THR123 | ['MG'] | No |
| 7YLL | P58965 | ASP103-GLU104-ASP9 | ['MG'] | Yes | 7MYJ | P54646 | GLU143-ASN144-ASP157 | ['MG'] | No |
| 7ZKX | P78362 | GLU229-ASN230-ASP541 | ['MG'] | Yes | 5KX6 | Q9SWH5 | ASP107-ASP109-SER110 | ['MG'] | No |
| 8A5X | P00720 | ASP394-GLU397-ASP398 | ['MG'] | No | 8W6X | P21852 | CYS546-CYS549-CYS81 | ['NI'] | No |
| 8ARV | Q9HW35 | ASP578-ASP579-GLU635 | ['MG'] | Yes | 8W6X | P21852 | CYS549-CYS81-CYS84 | ['NI'] | No |
| 8B31 | A0A2W4LV58 | ASP140-ASP141-ASP263 | ['MN'] | Yes | 4XZE | P27318 | ASP274-GLU277-ASP278 | ['MG'] | No |
| 8B3Y | A0A286RCT9 | ASP306-GLU309-ASP310 | ['MG'] | No | 7QM2 | P62136 | ASN124-HIS173-ASP92 | ['MN'] | No |
| 8C2Z | Q9Y463 | GLU243-ASN244-ASP259 | ['MG'] | Yes | 7QM2 | P62136 | HIS173-HIS248-ASP92 | ['MN'] | No |
| 8C5D | P19157 | ASP94-GLU97-ASP98 | ['MG'] | Yes | 7QM2 | P62136 | ASP64-HIS66-ASP92 | ['ZN'] | No |
| 8CWP | Q06282 | GLU157-GLU49-ASP51 | ['MG'] | Yes | 1MZC | P49354 | ASP317-GLU320-ASP321 | ['MG'] | Yes |
| 8F5D | A0A0T7CQ89 | THR123-THR145-GLU186 | ['MG'] | No | 4YU9 | Q16769 | HIS140-ASP159-ASP248 | ['ZN'] | Yes |
| 8GY1 | A0A8F4Y4C2 | GLU23-GLU58-HIS61 | ['FE'] | Yes | 5MYV | P78362 | GLU229-ASN230-ASP541 | ['MG'] | No |
| 8HCE | Q91XB0 | ASP18-ASP200-GLU20 | ['MG'] | Yes | 1WX5 | Q83WS2 | HIS190-HIS194-HIS216 | ['ZN'] | Yes |
| 8I82 | D2B3F1 | GLU193-ASN194-ASP211 | ['MG'] | Yes | 1WX5 | Q83WS2 | HIS38-HIS54-HIS63 | ['ZN'] | Yes |
| 8P0S | Q13464 | ASP104-GLU107-ASP108 | ['MG'] | No | 1SJD | Q44244 | ASP189-GLU214-ASP239 | ['MG'] | Yes |
| 8PNK | P06634 | LYS192-ASP306-GLU307 | ['MG'] | No | 3V11 | Q980A5 | THR23-THR46-ASP93 | ['MG'] | No |
| 8SSF | D3DIV8 | ASP140-GLU143-ASP144 | ['MG'] | No | 1KRQ | Q46106 | GLU17-GLU50-HIS53 | ['FE'] | Yes |
| 8TXY | Q7KZI7 | GLU179-ASN180-ASP193 | ['MG'] | No | 6NBA | P32929 | ASP385-GLU388-ASP389 | ['MG'] | Yes |
| 4AU8 | Q00535 | GLN130-ASN131-ASP144 | ['MG'] | Yes | 8E0Z | P0AB91 | HIS268-ASP326-CYS61 | ['MN'] | Yes |
| 4B3F | P38935 | LYS220-ASP375-GLU376 | ['MG'] | No | 2C47 | P78368 | GLU169-ASN170-ASP188 | ['MG'] | No |
| 4BFM | Q61846 | GLU136-ASN137-ASP150 | ['MG'] | No | 4M2E | G9MBV2 | HIS154-GLU156-HIS303 | ['FE'] | Yes |
| 4BJ4 | Q9HT86 | HIS153-ASP163-HIS42 | ['ZN'] | Yes | 5QP3 | Q8IU60 | GLU144-GLU148-GLU193 | ['MG'] | Yes |
| 4BUP | Q3U8K7 | ASP73-ASP74-THR77 | ['MG'] | Yes | 5YGR | P40817 | ASP392-GLU395-ASP396 | ['MG'] | No |
| 4C9B | P38919 | ASP187-GLU188-LYS88 | ['MG'] | Yes | 3C66 | P29468 | ASP100-ASP102-ASP154 | ['MG'] | No |
| 4CA7 | Q10714 | ASP304-ASP305-THR308 | ['MG'] | No | 7WVB | P09467 | ASP119-ASP122-GLU281 | ['MG'] | No |
| 4CE5 | Q0C8G1 | ASP74-ASP75-THR78 | ['MG'] | Yes | 7WVB | P09467 | ASP119-ASP122-GLU98 | ['MG'] | No |
| 4DEZ | A0QR77 | ASP107-GLU108-ASP9 | ['MG'] | Yes | 7WVB | P09467 | ASP119-GLU281-GLU98 | ['MG'] | No |
| 4DXK | Q7D1T6 | GLU216-GLU242-GLU268 | ['MG'] | Yes | 2IHM | Q9JIW4 | ASP330-ASP332-ASP420 | ['MG'] | No |
| 4EJ6 | Q92PZ3 | GLU143-HIS58-GLU59 | ['MN'] | No | 4EIW | Q5SI82 | HIS418-HIS422-ASP493 | ['ZN'] | No |
| 4EJ6 | Q92PZ3 | CYS37-HIS58-GLU59 | ['ZN'] | No | 4LNI | P12425 | ASP209-ASP210-THR213 | ['MG'] | No |
| 4EKN | Q58976 | ASP194-GLU197-ASP198 | ['MG'] | No | 1DV1 | P24182 | GLU276-GLU288-ASN290 | ['CA'] | Yes |
| 4F0Z | Q08209 | ASP118-ASN150-HIS199 | ['MN'] | Yes | 1JC5 | Q8VQN0 | HIS12-GLU141-HIS91 | ['ZN'] | Yes |
| 4F0Z | Q08209 | ASP118-HIS199-HIS281 | ['ZN'] | Yes | 6IG2 | O74339 | ASP164-GLU167-ASP168 | ['MG'] | Yes |
| 4F0Z | Q08209 | ASP118-ASP90-HIS92 | ['ZN'] | Yes | 5W16 | A0A1J1ER74 | ASP85-GLU88-ASP89 | ['MG'] | Yes |
| 4F4R | Q1QT89 | ASP211-GLU237-GLU263 | ['MG'] | Yes | 7A8R | A0JN36 | ASP299-GLU302-ASP303 | ['MG'] | No |
| 4F50 | Q4JB80 | ASP105-GLU106-ASP7 | ['MG'] | Yes | 8GSQ | P00441 | HIS120-HIS46-HIS48 | ['ZN'] | Yes |
| 4FAI | Q86PD7 | HIS137-ASP153-ASP228 | ['ZN'] | No | 8E9E | Q04631 | ASP317-GLU320-ASP321 | ['MG'] | No |
| 4FK9 | G2NHM6 | ASP285-ASP287-THR288 | ['MG'] | Yes | 1WZO | Q5SJQ0 | ASP128-GLU97-GLU99 | ['MG'] | Yes |
| 4FU0 | Q6WRY5 | GLU226-ASP299-GLU312 | ['MG'] | Yes | 3C4E | P11309 | GLU171-ASN172-ASP186 | ['MG'] | No |
| 4FZX | P0AEK0 | HIS134-ASP139-GLU8 | ['MG'] | Yes | 3WCL | P37268 | ASP80-GLU83-ASP84 | ['MG'] | Yes |
| 4FZX | P0AEK0 | ASP139-ASP6-GLU8 | ['MG'] | Yes | 5XVD | A0A3B6UEQ1 | CYS549-CYS61-CYS64 | ['NI'] | No |
| 4GV1 | P31749 | GLU278-ASN279-ASP292 | ['MG'] | Yes | 8HCH | Q91XB0 | HIS195-ASP200-GLU20 | ['MG'] | Yes |
| 4I2A | P09838 | ASP343-ASP345-ASP434 | ['MG'] | No | 2PGW | Q92YR6 | ASP196-GLU222-ASP247 | ['MG'] | Yes |
| 4IIK | Q5ZSQ2 | ASP110-ASP91-ASP92 | ['MG'] | Yes | 1UVN | P11124 | ASP327-ASP329-THR330 | ['MG'] | No |
| 4IIT | A6T8I0 | GLU42-GLU72-HIS75 | ['FE'] | No | 4YFU | E1C9K5 | ASP653-ASP830-GLU831 | ['MG'] | Yes |
| 4IR8 | B9PW60 | ASP107-ASP110-GLU274 | ['MG'] | Yes | 2ZE8 | P58758 | ASP173-GLU213-HIS214 | ['ZN'] | No |
| 4IR8 | B9PW60 | ASP107-GLU274-GLU88 | ['MG'] | Yes | 3MP4 | P35914 | HIS233-HIS235-ASP42 | ['ZN'] | No |
| 4J6O | A3DJ38 | ASP233-ASN263-HIS323 | ['MN'] | No | 2YWB | Q5SI28 | ASP406-ASP407-THR410 | ['MG'] | Yes |
| 4J6V | B2ZB02 | HIS204-HIS208-HIS231 | ['ZN'] | Yes | 1U2D | Q2RSB2 | ASP47-ASP48-THR51 | ['MG'] | No |
| 4JD0 | G4FFF4 | ASP107-GLU217-ASP219 | ['MG'] | Yes | 5CU9 | Q5AD07 | HIS153-HIS75-HIS77 | ['ZN'] | No |
| 4JID | A0A6L8PDI9 | HIS160-ASP167-HIS219 | ['ZN'] | Yes | 4RVP | A0A0A0QA66 | HIS132-HIS58-HIS60 | ['ZN'] | No |
| 4JZU | O35013 | GLU115-GLU68-GLU72 | ['MG'] | Yes | 7CBB | Q2G1N1 | GLN426-ASN427-ASP444 | ['MG'] | No |
| 4K25 | P43122 | ASP43-ASP44-ASP67 | ['MG'] | No | 3EWS | Q9UMR2 | LYS144-ASP242-GLU243 | ['MG'] | No |
| 4K3Z | Q8YCV0 | GLU160-ASP196-HIS227 | ['MN'] | Yes | 1JQW | O34667 | ASP103-GLU106-ASP107 | ['MG'] | No |
| 4K7E | Q6IVU2 | ASP380-GLU382-ASP529 | ['ZN'] | No | 1Z9X | Q9UIK4 | GLU143-ASN144-ASP161 | ['MG'] | No |
| 4K89 | A7LI11 | TYR155-GLU164-HIS223 | ['ZN'] | No | 5G5T | Q58717 | ASP504-ASP570-ASP688 | ['MN'] | No |
| 4KLI | P06746 | ASP190-ASP192-ASP256 | ['MG'] | No | 3WID | Q979W2 | GLU156-HIS68-GLU69 | ['MN'] | Yes |
| 4KM3 | B2IQ22 | ASP136-GLU264-ASP98 | ['MG'] | No | 3WID | Q979W2 | CYS41-HIS68-GLU69 | ['ZN'] | Yes |
| 4KP6 | Q07343 | ASP471-GLU474-ASP475 | ['MG'] | Yes | 3B5Q | Q8A7C8 | ASP438-ASP439-THR442 | ['MG'] | No |
| 4KRD | P17157 | GLN137-ASN138-ASP151 | ['MG'] | Yes | 6PQH | X5KA67 | ASP262-GLU265-ASP266 | ['MG'] | Yes |
| 4LIM | P10363 | ASP111-ASP113-HIS168 | ['CD'] | Yes | 2XAU | P53131 | ASP314-GLU317-ASP318 | ['MG'] | No |
| 4LIM | P10363 | ASP111-ASP113-ASP314 | ['MN'] | No | 3OVB | O28126 | ASP110-GLU59-ASP61 | ['MG'] | No |
| 4MHP | B7QK46 | HIS128-ASP144-ASP238 | ['ZN'] | Yes | 1AQW | P09211 | ASP94-GLU97-ASP98 | ['MG'] | Yes |
| 4MHP | B7QK46 | ASP144-GLU184-HIS322 | ['ZN'] | Yes | 2OZ3 | C1DMY1 | ASP214-GLU240-GLU268 | ['MG'] | Yes |
| 4MHR | Q1GNW5 | HIS144-ASP146-HIS245 | ['ZN'] | Yes | 6E4R | Q8MRC9 | ASP301-HIS303-HIS437 | ['MN'] | No |
| 4N81 | Q5NMB8 | ASP210-ASP86-ASP89 | ['MG'] | No | 5ES1 | Q96L34 | GLU182-ASN183-ASP196 | ['MG'] | No |
| 4NMI | Q2TDY4 | HIS146-ASP148-HIS248 | ['ZN'] | Yes | 5ESR | Q9A919 | HIS22-HIS38-ASP40 | ['ZN'] | Yes |
| 4NU1 | Q9WV60 | ASP190-ASP192-THR193 | ['MG'] | No | 5FJ6 | P11124 | ASP327-ASP329-THR330 | ['MG'] | No |
| 4O7I | C4M4T9 | ASP118-ASP121-ASP257 | ['MG'] | Yes | 5G2U | Q8A7C8 | ASP291-HIS292-ASP32 | ['ZN'] | Yes |
| 4OA8 | Q2GKC7 | ASP136-ASP162-ASN163 | ['MG'] | No | 5G5S | Q58717 | ASP504-ASP570-ASP688 | ['MG'] | No |
| 4OKE | P9WJ73 | ASP145-ASP6-GLU8 | ['MG'] | No | 5GGB | A0QUZ2 | GLU127-GLU81-GLU85 | ['MG'] | Yes |
| 4ONW | Q9KQ52 | ASP101-GLU136-HIS239 | ['ZN'] | Yes | 5GJB | A0A0U4DG08 | LYS200-ASP285-GLU286 | ['MG'] | No |
| 4P4M | Q9U6N3 | ASP194-ASP196-ASP271 | ['MG'] | No | 5GJU | P0A9P6 | ASP156-GLU157-LYS56 | ['MG'] | No |
| 4P4M | Q9U6N3 | ASP345-GLU348-ASP349 | ['MG'] | Yes | 5HRB | P42494 | SER39-ASP49-ASP51 | ['MG'] | Yes |
| 4PF4 | P53355 | GLU143-ASN144-ASP161 | ['MG'] | Yes | 5HVN | Q5NFS1 | GLU184-HIS247-HIS264 | ['ZN'] | No |
| 4PP4 | P03134 | GLU119-HIS127-HIS129 | ['ZN'] | Yes | 5HX4 | Q8IUX4 | HIS249-CYS280-CYS283 | ['ZN'] | No |
| 4TW3 | Q7V6D4 | ASP210-GLU213-ASP214 | ['MG'] | No | 5HZX | Q7ZWC3 | GLU100-GLU52-GLU56 | ['MG'] | No |
| 4U4I | G5CS11 | HIS128-HIS54-HIS56 | ['ZN'] | No | 5I0N | D9IEF7 | ASP396-GLU399-ASP400 | ['MG'] | Yes |
| 4UD4 | O13833 | ASP101-ASP103-ASP160 | ['MG'] | No | 5IG1 | F2UPG5 | GLU144-ASN145-ASP161 | ['MG'] | Yes |
| 4W9W | Q9NSY1 | GLU184-ASN185-ASP198 | ['MG'] | Yes | 5INB | P36873 | ASN124-HIS173-ASP92 | ['MN'] | Yes |
| 4WNO | O75385 | GLN142-ASN143-ASP165 | ['MG'] | Yes | 5INB | P36873 | HIS173-HIS248-ASP92 | ['ZN'] | Yes |
| 4XAE | Q9LHN8 | HIS235-ASP237-HIS293 | ['ZN'] | No | 5INB | P36873 | ASP64-HIS66-ASP92 | ['ZN'] | Yes |
| 4XIA | P12070 | GLU216-HIS219-ASP254 | ['MG'] | Yes | 5J41 | P09211 | ASP94-GLU97-ASP98 | ['MG'] | Yes |
| 4XIA | P12070 | GLU216-ASP254-ASP256 | ['MG'] | Yes | 5JM9 | P14904 | ASP259-GLU296-HIS435 | ['ZN'] | No |
| 4Y7E | F5HR99 | ASP285-ASP287-THR288 | ['MG'] | Yes | 5KIA | Q2T9E1 | CYS38-HIS63-GLU64 | ['ZN'] | Yes |
| 4YLM | Q9I1L4 | HIS110-ASP112-HIS259 | ['ZN'] | No | 5KRY | Q8DBF5 | ASP150-GLU152-GLU231 | ['MG'] | Yes |
| 4Z0U | A7ZHV1 | ASP10-ASP134-ASP70 | ['MG'] | No | 5LF8 | P0C025 | GLU142-GLU146-GLU207 | ['MG'] | Yes |
| 4Z0U | A7ZHV1 | ASP10-GLU48-ASP70 | ['MG'] | Yes | 5MZG | P53368 | GLU100-GLU52-GLU56 | ['MG'] | No |
| 4Z1O | D0KMY9 | ASP10-GLU13-ASP14 | ['MG'] | No | 5N3J | P25321 | GLU170-ASN171-ASP184 | ['MG'] | Yes |
| 4Z5S | Q55688 | GLU32-GLU60-HIS63 | ['FE'] | Yes | 5NG0 | O43353 | GLN150-ASN151-ASP164 | ['MG'] | Yes |
| 4Z84 | P00517 | GLU170-ASN171-ASP184 | ['MG'] | Yes | 5O9X | A0A0S7E9S6 | ASP284-ASP286-ASP288 | ['MG'] | Yes |
| 4ZFQ | P9WKV2 | ASP233-ASP235-HIS256 | ['CD'] | No | 5O9X | A0A0S7E9S6 | ASP526-ASP527-THR530 | ['MG'] | No |
| 4ZN6 | B7H1U5 | ASP159-GLU161-GLU239 | ['MG'] | Yes | 5OVO | A7XNI2 | ASP245-THR59-ASP60 | ['MG'] | No |
| 4ZRL | O17087 | ASP606-ASP608-ASP668 | ['MG'] | Yes | 5Q22 | Q6PJP8 | ASP736-HIS737-ASP815 | ['ZN'] | Yes |
| 5A67 | Q9SIY3 | GLU171-GLU2-GLU4 | ['MG'] | Yes | 5BXX | Q1GNW6 | GLU57-TYR85-HIS93 | ['FE'] | Yes |
| 5AJO | Q10471 | ASP224-HIS226-HIS359 | ['MN'] | No | 5C7Q | Q6MIH8 | GLU140-GLU92-GLU96 | ['MG'] | Yes |
| 5AVH | P24300 | GLU217-HIS220-ASP255 | ['MG'] | Yes | 5CA8 | Q9C0L9 | ASP365-GLU368-ASP369 | ['MG'] | Yes |
| 5AVH | P24300 | GLU217-HIS220-ASP257 | ['NI'] | No | 5CI1 | A0A0E1LZC3 | GLU115-HIS118-ASP84 | ['MN3'] | Yes |
| 5AVH | P24300 | GLU217-ASP255-ASP257 | ['MG'] | No | 5CI1 | A0A0E1LZC3 | ASP138-ASP139-THR142 | ['MG'] | Yes |
| 5B4B | Q9I2V0 | HIS10-HIS197-ASP8 | ['ZN'] | Yes | 5CI3 | P69925 | GLU115-HIS118-ASP84 | ['MN3'] | Yes |
| 5B4B | Q9I2V0 | HIS10-ASP41-ASP8 | ['ZN'] | Yes | 5CUX | Q4JH30 | ASP291-ASP296-ASP328 | ['MG'] | No |
| 5B4B | Q9I2V0 | HIS114-HIS195-ASP41 | ['ZN'] | No | 5DKT | Q8ILY1 | ASP410-ASP579-GLU580 | ['MG'] | No |
| 5B4B | Q9I2V0 | HIS114-ASP41-ASN79 | ['MN'] | No | 5EKW | O23346 | HIS145-HIS73-GLU77 | ['MN'] | Yes |
| 5BQP | Q97W22 | ASP10-GLU13-ASP14 | ['MG'] | No | 5EKW | O23346 | HIS169-GLU173-HIS47 | ['MN'] | No |

^a^ Predicted residue triplet that may bind with a metal ion.

^b^ The most likely metal ion for the predicted residue triplet binding.

^c^ In the PDB structure, we used the Win-Coot program to view whether there is electron density for predicted metal ion.

**Table S5**. The details of potential bi-metal-binding sites identified by MeSiteIG.

| **PDB** | **UniProt**  **ID** | **Predicted Triplet^a^** | **Most likely metal ions^b^** | **Annotated Triplet^c^** | **Annotated metal ion^d^** | **Distance^e^** |
| --- | --- | --- | --- | --- | --- | --- |
| 1T5J | Q58588 | ASP253-ASP255-SER256 | ['MG'] | ASP255-THR60-ASP61 | ['MG'] | 3.9 |
| 1XWY | P27859 | HIS127-HIS152-GLU91 | ['ZN'] | HIS152-CYS153-GLU201 | ['ZN'] | 5.8 |
| 2AFW | Q16769 | HIS140-ASP159-ASP248 | ['ZN'] | ASP159-GLU202-HIS330 | ['ZN'] | 3.9 |
| 2Q2L | B2CP37 | HIS119-HIS45-HIS47 | ['ZN'] | HIS62-HIS70-HIS79 | ['ZN'] | 6.2 |
| 3PB6 | Q9NXS2 | HIS168-ASP186-ASP269 | ['ZN'] | ASP186-GLU226-HIS351 | ['ZN'] | 4 |
| 3SI2 | Q9CYK2 | HIS141-ASP160-ASP249 | ['ZN'] | ASP160-GLU203-HIS331 | ['ZN'] | 3.9 |
| 6G1P | H3BCW1 | ASP303-ASP305-THR306 | ['MG'] | ASP305-THR62-ASP63 | ['MG'] | 3.4 |
| 6IFQ | A0A0H3AJ04 | ASP158-ASP159-GLU215 | ['MG'] | ASP158-GLU45-ASN95 | ['MG'] | 5 |
| 6IUX | P54922 | ASP302-ASP304-SER305 | ['MG'] | ASP304-SER54-ASP55 | ['MG'] | 3.7 |
| 4FAI | Q86PD7 | HIS137-ASP153-ASP228 | ['ZN'] | ASP153-GLU191-HIS318 | ['ZN'] | 3.9 |
| 4J6V | B2ZB02 | HIS204-HIS208-HIS231 | ['ZN'] | HIS42-HIS60-HIS69 | ['ZN'] | 3.2 |
| 5CI1 | A0A0E1LZC3 | GLU115-HIS118-ASP84 | ['MN3'] | GLU204-GLU238-HIS241 | ['MN3'] | 2.9 |
| 4YU9 | Q16769 | HIS140-ASP159-ASP248 | ['ZN'] | ASP159-GLU202-HIS330 | ['ZN'] | 3.9 |
| 8GSQ | P00441 | HIS120-HIS46-HIS48 | ['ZN'] | HIS63-HIS71-HIS80 | ['ZN'] | 6.2 |
| 3GTT | P08228 | HIS120-HIS46-HIS48 | ['ZN'] | HIS63-HIS71-HIS80 | ['ZN'] | 6.2 |
| 6QRO | G8UMP8 | HIS126-ASP151-ASP224 | ['ZN'] | ASP151-ASP184-HIS305 | ['ZN'] | 4.2 |
| 4S17 | A1A1Z1 | GLU134-HIS277-GLU367 | ['MN'] | GLU136-GLU220-GLU228 | ['MG'] | 5.6 |
| 2UU7 | Q8HZM5 | GLU134-HIS253-GLU338 | ['MN'] | GLU136-GLU196-GLU203 | ['MG'] | 4.8 |
| 6ZS1 | G0S1F8 | HIS121-HIS47-HIS49 | ['ZN'] | HIS64-HIS72-HIS81 | ['ZN'] | 6.3 |
| 1X8G | P26918 | ASN116-HIS118-HIS196 | ['ZN'] | ASP120-CYS221-HIS263 | ['ZN'] | 3.7 |

^a^ Predicted residue triplet that may bind with a metal ion.

^b^ The most likely metal ion for the predicted residue triplet binding.

^c^ Annotated residue triplet.

^d^ Annotated metal ion in known metal site.

^e^ The distance between predicted metal ion and known metal ion.
